# A Cavity-Shaped Gold(I) Fragment Enables CO_2_ Insertion into Au–OH and Au–NH Bonds

**DOI:** 10.1021/acs.inorgchem.3c00751

**Published:** 2023-06-27

**Authors:** Miquel Navarro, Markus Holzapfel, Jesús Campos

**Affiliations:** Instituto de Investigaciones Químicas (IIQ), Departamento de Química Inorgánica and Centro de Innovación en Química Avanzada (ORFEO-CINQA), Consejo Superior de Investigaciones Científicas (CSIC) and University of Sevilla, Sevilla 41092, Spain

## Abstract

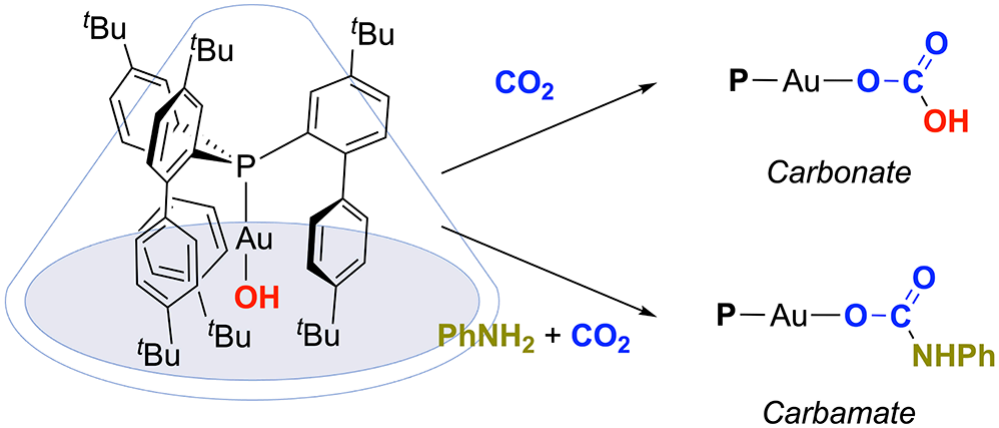

A cavity-shaped linear gold(I) hydroxide complex acts as a platform to access unusual gold monomeric species. Notably, this sterically crowded gold fragment enables the trapping of CO_2_ via insertion into Au–OH and Au–NH bonds to form unprecedented monomeric gold(I) carbonate and carbamate complexes. In addition, we succeeded in the identification of the first gold(I) terminal hydride bearing a phosphine ligand. The basic nature of the Au(I)-hydroxide moiety is also explored through the reactivity toward other molecules containing acidic protons such as trifluoromethanesulfonic acid and terminal alkynes.

## Abstract 2

We have explored the insertion of the chemistry of CO_2_ into Au−OH and Au−NH bonds within the confining environment of a cavity-shaped phosphine. This steric protection enables to access a variety of unusual species, such as monomeric gold(I) carbonate, carbamate, urea, or hydride complexes.

## Introduction

For a long time, gold was considered a noble metal too chemically inert to be used in catalysis.^1^ However, during the last decades, gold catalysis has evolved into a powerful tool for the synthesis of complex organic compounds, and the number of novel gold-mediated reactions has increased exponentially.^2^ In this regard, the rational design of ligands has played a crucial role in the development of new transformations at gold, such as oxidative addition, migratory insertion, or β-hydrogen elimination,^3^ which has opened the door for the discovery of new catalytic transformations. Additionally, the development of new ligands has permitted the isolation of unusual gold species that had been elusive for years and that have been considered as catalytic intermediates in the aforementioned transformations.^4^ Our group has contributed to this area by using sterically demanding phosphine ligands to access unusual Au(I) structures such as hydrocarbyl-bridged cationic digold complexes^5^ and dicoordinate gold(I) ethylene species,^6^ together with their use as FLP constituents.^7^

In the organometallic chemistry of gold, the choice of a suitable precursor to access more sophisticated structures is crucial. In particular, gold hydroxide complexes, which have been isolated both in their Au(I) and Au(III) forms,^8^ have been identified as versatile synthetic reagents and have given access to multiple transformations involving hydroxide intermediates. More specifically, gold(I) hydroxides have been prepared and isolated as stable species using N-heterocyclic carbenes (NHCs) as supporting ligands,^9,10^ mainly due to their strong donor ability and their wingtip structure. However, the use of phosphine ligands to prepare this type of species has been scarcely described, most likely due to their relatively weaker donor capacity and their conical structure with lesser protection on the metal center.^11^ In previous studies, we have shown that the use of the exceptionally bulky tris-2-(4,4′-di-*tert*-butylbiphenylyl)phosphine^12^ generates a protective cavity around the gold center that provides a remarkable kinetic stability to otherwise unstable compounds^13^ Herein, we describe the preparation of the corresponding gold hydroxide synthon and explore its reactivity toward different substrates, showing the ability of the extremely bulky phosphine to stabilize unusual monomeric gold species.

## Results and Discussion

**Scheme 1:**
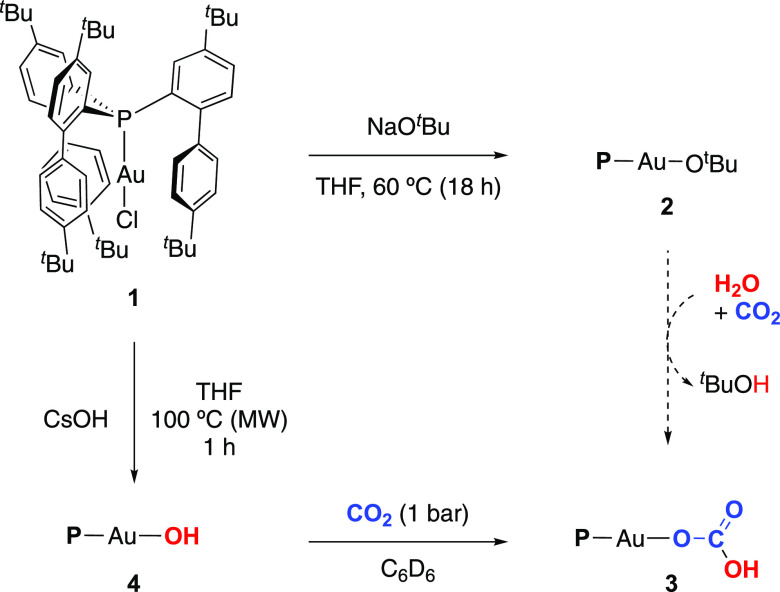
Synthesis of Gold Complexes **2** and **4**, and Formation of the Gold(I) Hydrogen Carbonate Complex **3**

Initially, we examined the reaction of complex (P-ligand)AuCl **1** (P-ligand = tris-2-(4,4′-di-*tert*-butylbiphenylyl)phosphine, defined in Figures and Schemes as P for simplicity) with NaO^*t*^Bu in tetrahydrofuran (THF) to generate the desired gold compound (P-ligand)AuO^*t*^Bu **2** through a metathesis reaction (Scheme 1). Although the reaction did not proceed to any extent at room temperature, an increase to 50 °C was sufficient to afford complete conversion toward **2** after 18 h. This supports the robustness of complex **1** since prior related gold systems readily react with NaO^*t*^Bu under milder conditions.^9a,10^ Remarkably, the stability of complex **2** was limited under air, slowly leading to a new gold(I) species that was successfully identified by X-ray crystallographic means as the first terminal gold hydrogen carbonate compound **3**. These species, which are known for other transition metals,^14^ have been previously postulated as intermediates in gold chemistry, but they had so far escaped detection due to the rapid formation of trigold carbonate compounds in all cases.^15^ Herein, we presume that those trimetallic species are not accessible due to the extreme steric constraints imposed by the cavity-shaped phosphine, demonstrating once more the peculiar chemistry imparted by this ligand.

**Figure 1:**
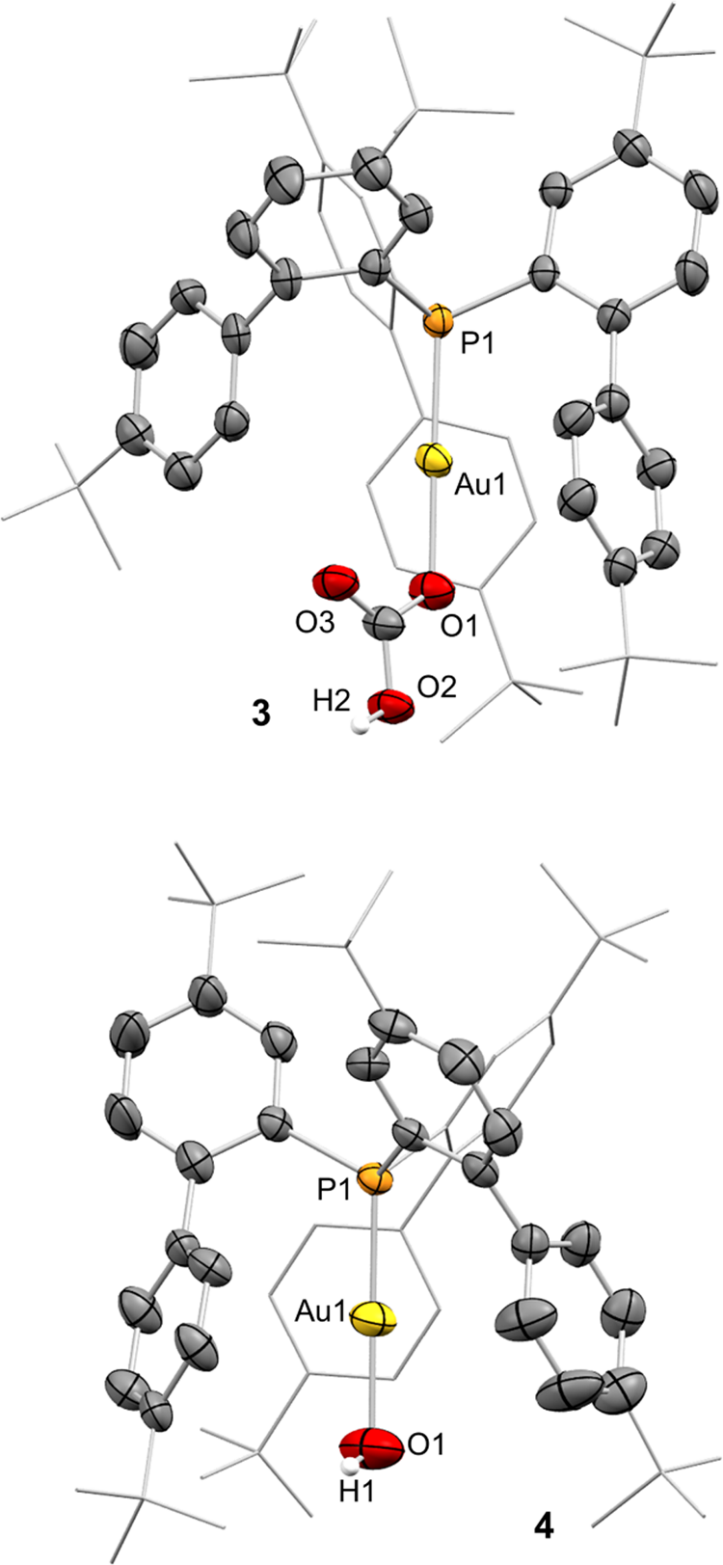
Oak ridge thermal ellipsoid plot (ORTEP) representation of complexes **3** and **4**. Only one of the two independent molecules in **3** is represented. Thermal ellipsoids are set at 50% probability. Most hydrogen atoms and solvent molecules are excluded for clarity, while *tert*-butyl groups and one biaryl fragment are represented in wireframe format. Selected bond length (Å) and angles (°): compound **3**, P1–Au1, 2.2043(16); Au1–O1, 2.055(5); P1–Au1–O1, 179.00(13); compound **4**, P1–Au1, 2.2034(17); Au1–O1, 2.045(6); P1–Au1–O1, 178.9(2).

We managed to grow crystals of complex **3** by slow diffusion of pentane into its dichloromethane solution. Complex **3** crystallized with two independent molecules per unit cell that are linked through hydrogen-bonding interactions through the hydrogen carbonate moieties (*d*_O_2_H_2_···O_5__ = 1.827(7), *d*_O_6_H_6_···O_3__ = 1.709(7) Å; O2–H2···O5 = 165.6(9), O6–H6···O3 = 175.3(9)°; see Figure S38). Each molecule presents a linear geometry with P–Au–O angles of 177.0(1) and 179.0(1)° and covalent Au–O bonds for the two independent molecules of 2.076(4) and 2.054(4) Å (Figure 1). Compound **3** is characterized by a resonance at 3.6 ppm by ^31^P{^1^H} NMR spectroscopy. In turn, its ^1^H NMR spectrum showed a characteristic COOH broad signal at 12.28 ppm, while the carbonyl carbon was identified as a singlet at 163.4 ppm in the ^13^C{^1^H} NMR spectrum. In addition, the IR spectrum of complex **3** shows three different bands at 1618, 1453, and 1330 cm^–1^ corresponding to three different C–O stretching frequencies of the nonsymmetric OCOOH unit (Figure S37), supporting the terminal coordination mode observed by X-ray diffraction studies.^16^

We hypothesize that complex **2** hydrolyzes in the presence of moisture forming a gold(I) hydroxide complex and ^*t*^BuOH, followed by trapping atmospheric CO_2_ by insertion into the Au–OH bond (Scheme 1). For this reason, we envisioned the synthesis of the purported gold(I) hydroxide complex as a versatile gold(I) synthon.^9^ Reactions of complex **1** with different alkali metal (M = Li, Na, K or Cs) hydroxides were attempted; however, CsOH·H_2_O resulted to be the only one capable of generating the desired gold hydroxide complex **4**. Thus, **4** was best prepared by reacting **1** with an excess of CsOH·H_2_O (10 equiv) at 100 °C under microwave irradiation for 1 h, affording the complex as a white powder in excellent yields (ca. 90%) (Scheme 1). Spectroscopic characterization of **4** showed a doublet at −1.50 ppm (^3^*J*_HP_ = 5.9 Hz) in the ^1^H NMR spectrum corresponding to the Au–OH proton signal. In addition, ^31^P{^1^H} NMR spectroscopy revealed a singlet at 9.1 ppm, which is only 0.4 and 3.1 ppm upfield shifted in comparison to complexes **1** and **2**, respectively. Single-crystal X-ray diffraction analysis confirmed as well the linear geometry of complex **4**, with a P1–Au1–O1 angle of 178.9(2)° and a covalent Au–O bond of 2.045(9) Å, slightly shorter than in complex **3** (Figure 1).

We then monitored by ^31^P{^1^H} NMR spectroscopy the reaction of **4** with CO_2_ (1 bar) in C_6_D_6_, revealing the foreseen and immediate appearance (full conversion within less than 5 min at room temperature) of the aforementioned singlet at 3.6 ppm corresponding to the hydrogen carbonate compound **3** (Scheme 1). This result is fully consistent with our hypothesized CO_2_ insertion into the Au–OH bond. To further support this path, we have also studied the CO_2_ insertion reaction by means of density functional theory (DFT) calculations, which revealed an exergonic single-step 1,2-addition of the CO_2_ molecule into the Au–OH bond with a small energetic barrier of 3.9 kcal/mol that perfectly fits with the spontaneous formation of **3** under air atmosphere at room temperature (Figure S39).

**Figure 2:**
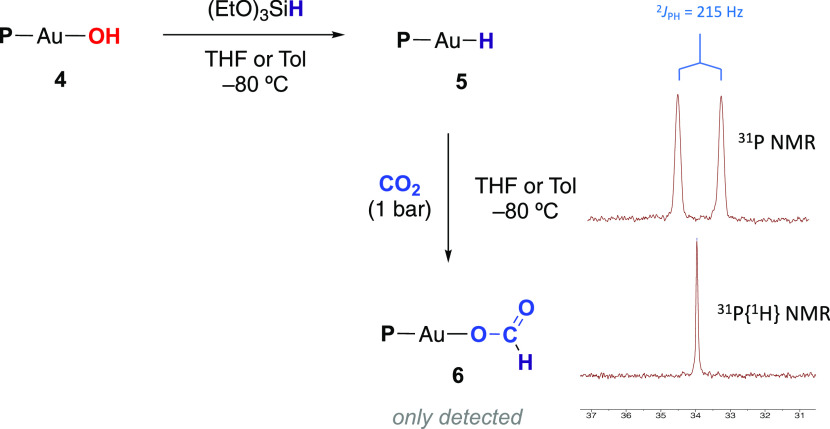
Synthesis of gold(I) hydride complex **5**, CO_2_ insertion into the Au–H bond forming the gold(I) formate complex **6** and ^31^P{^1^H} and ^31^P NMR spectra of complex **5**.

In previous studies, we have demonstrated that the cavity-shaped protection enforced by the exceptionally bulky tris-2-(4,4′-di-*tert*-butylbiphenylyl)phosphine provides a remarkable kinetic stabilization to otherwise unstable compounds, such as the corresponding gold(I)–ethylene and gold(I)-carbonyl complexes of type [(P-ligand)AuL]^+^ (L = C_2_H_4_, CO).^13^ On this bases, we now sought to utilize this ligand to access a monomeric Au(I) hydride species,^17^ examples of which have been isolated only using strong donor N-heterocyclic carbenes (NHCs)^9a,18^ and NHC-coordinated diphosphene ligands.^19^ Gold hydrides have been considered as intermediates in numerous gold-catalyzed organic transformations.^20^ Having in mind that phosphines are the most common and widely used ligands in gold catalysis, it is obvious that the isolation of a monomeric phosphine-based gold(I) hydride complex is of notable importance within the organometallic chemistry of gold. With this synthetic aim, we attempted the independent reactions of complexes **1**, **2**, and **4** with LiBEt_3_H and *N*-selectride, which did not lead to any apparent transformation. In contrast, the reaction of complexes **2** or **4** with (EtO)_3_SiH at −78 °C in either THF or toluene did result in the formation of the target gold(I) hydride complex **5** (Figure 2). Nonetheless, compound **5** only showed relative stability in solution at low temperatures, decomposing above −30 °C to generate elemental Au and free phosphine.

The ^31^P NMR spectrum of complex **5** exhibits a doublet at 34.0 ppm, with an associated scalar coupling of ^2^*J*_PH_ = 215 Hz, a considerably higher value than in previous reports on the terminal Au-hydride compound based on the NHC-coordinated diphosphene ligand (^2^*J*_PH_ = 138 Hz) (Figure 2).^19^ In the ^1^H NMR spectrum, a doublet at 3.51 ppm (^2^*J*_PH_ = 215 Hz), which becomes a singlet by ^31^P decoupling, can be unambiguously assigned to the Au–H resonance. Interestingly, it is significantly upfield shifted in comparison to that of the NHC-coordinated diphosphene^19a^ (4.60 ppm) and NHC-stabilized^9a,18a^ Au(I)-hydride (5.11 ppm). Unfortunately, the isolation of complex **5** proved unsuccessful due to its limited stability even at temperatures below −40 °C and therefore, its spectroscopic characterization was carried out *in situ* at −80 °C in THF-*d*_8_ (see the Experimental Section for further details). We have also studied the hydride formation reaction by means of density functional theory (DFT) calculations, which revealed an exergonic process with a small energetic barrier of 3.7 kcal/mol that perfectly fits with the instantaneous formation of **5** at low temperature (Figure S41). Similarly, to the reactivity of complex **4** toward CO_2_, we investigated the hydridic character of the Au–H moiety by exposing a solution of **5** to CO_2_ at −30 °C. Although we could indeed detect the insertion of CO_2_ into the Au–H bond to yield what we assume is the anticipated gold(I) formate complex **6**,^21^ this could only be detected as a minor product since both complexes **5** and **6** decomposed under reaction conditions (−30 °C) even before the insertion reaction is complete, generating once more elemental Au and free phosphine. In any case, compound **6** was identified by ^31^P{^1^H} (singlet at 2.71 ppm) and ^1^H NMR (characteristic doublet at 8.71 ppm, ^4^*J*_PH_ = 13 Hz) (Figures 2 and S16–S18).

**Figure 3:**
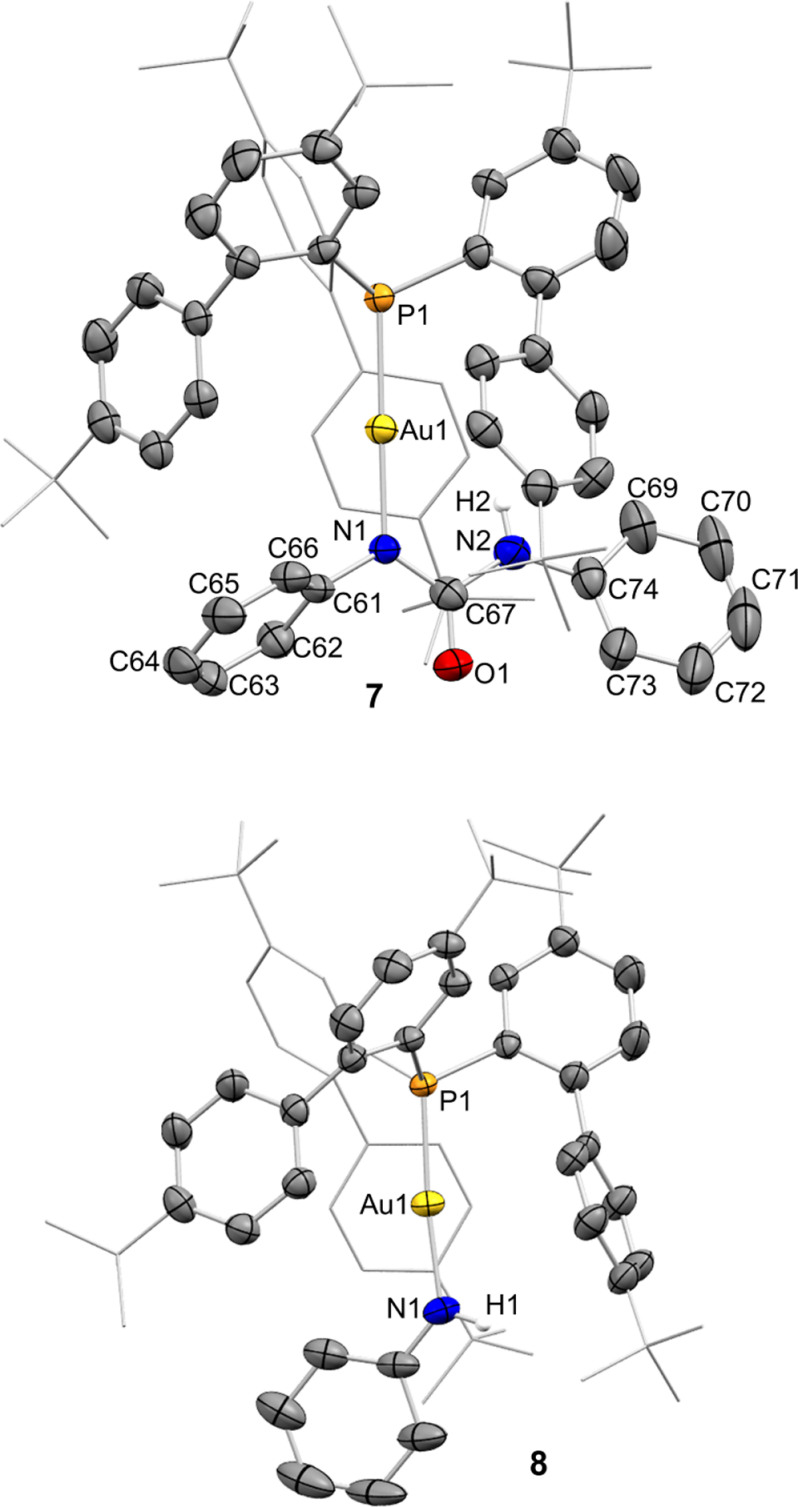
ORTEP representation of complexes **7** and **8**. Only one of the two independent molecules in **7** is represented. Thermal ellipsoids are set at 50% probability. Most hydrogen atoms and solvent molecules are excluded for clarity, while *tert*-butyl groups and one biaryl fragment are represented in wireframe format. Selected bond length (Å) and angles (°): compound **7**, P1–Au1, 2.2343(13); Au1–N1, 2.077(4); P1–Au1–N1, 176.93(13); compound **8**, P1–Au1, 2.2310(7); Au1–N1, 2.026(2); P1–Au1–N1, 174.67(8).

**Scheme 2:**
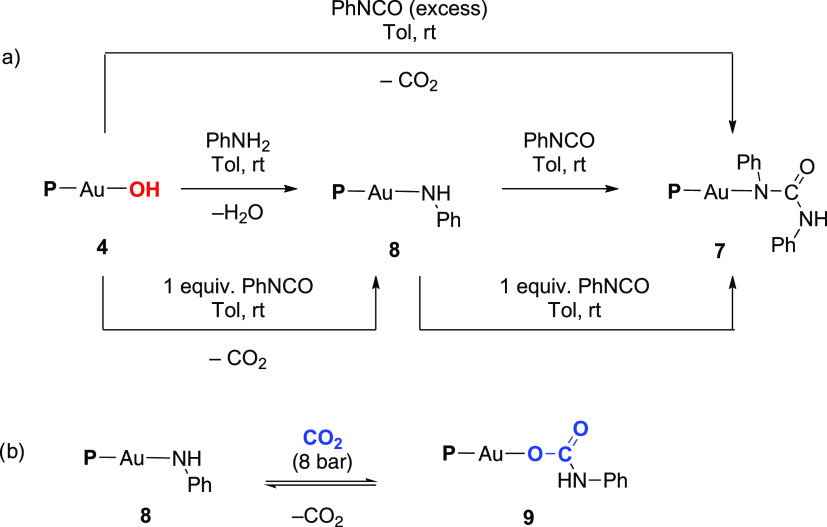
(a) Synthesis of Gold Complexes **7** and **8**, and (b) Reversible CO_2_ Insertion into a Au–NH Bond Forming Gold(I) Carbamate Complex **9**

We next decided to investigate whether other small molecules would also insert into the Au–OH bond of complex **4** to access unusual Au(I) terminal species. Although the reaction of **4** with CS_2_, CO, and isocyanides leads to decomposition through the formation of elemental gold and free phosphine, the addition of the heterocumulene phenyl isocyanate resulted in clean formation of a new species that exhibited a sole singlet in the ^31^P{^1^H} NMR spectrum at 11.2 ppm (Scheme 2a). The new gold(I) complex **7** presented a complicated ^1^H NMR spectrum in the aromatic region that implied the presence of more than one phenyl ring derived from the phenyl isocyanate substrate, and a characteristic singlet at 5.58 ppm that we attributed to an NH proton. The structure of complex **7** was unequivocally confirmed by single-crystal X-ray diffraction, showing a linear neutral gold(I) complex (P1–Au1–N1, 178.8(1)°) coordinated to a deprotonated diphenyl urea with the carbonyl group pointing opposite to the gold cavity (Figure 3).

The unexpected formation of diphenyl urea in this transformation prompted us to study the mechanism of this reaction. The chemistry of cationic gold(I) species has been associated in many cases to sophisticated protons by means of the isolobal analogy,^22^ and therefore we hypothesize that complex **4** could be acting similarly to a water molecule. For this reason, we examined the reactivity of phenyl isocyanate with water in our hands,^23^ which lead after 18 h at 25 °C to the formation of aniline and release of CO_2_. Then, a second addition of phenyl isocyanate afforded diphenyl urea. Independently, the addition of an equimolar amount of phenyl isocyanate to aniline also leads to the formation of diphenyl urea. Then, we examined the reaction of complex **4** with aniline, which yielded the gold(I) amido complex **8** quantitatively through a typical protonolysis reaction generating a molecule of water (Scheme 2a). ^31^P{^1^H} NMR spectroscopy revealed the appearance of a singlet at 16.1 ppm, and the ^1^H NMR spectrum showed a characteristic resonance at 2.78 ppm corresponding to the NH unit together with the appearance of a broad singlet at 0.4 ppm that corresponds to the formation of water. Single-crystal X-ray diffraction analysis confirmed once more the expected linear geometry of complex **8** with a P1–Au1–N1 angle of 174.66(8)° and a covalent Au–N1 bond of 2.026(3) Å (Figure 3). As foreseen, the equimolar reaction between complex **8** and phenyl isocyanate afforded the diphenyl urea derivative **7** (Scheme 2a). In view of these results, we propose a two-step reaction in which the hydroxy gold complex **4** reacts with phenyl isocyanate to generate in a first step the amido complex **8** with concomitant release of CO_2_, which reacts further with a second molecule of phenyl isocyanate to produce the diphenyl urea complex **7**.

In view of the ability of complex **4** to trap CO_2_ through insertion into the Au–OH bond, we envisioned the possibility of trapping CO_2_ into the Au–NH bond of complex **8**. The reaction of amines with carbon dioxide to generate carbamic acid is a well-studied industrial process;^24^ however, the reaction of CO_2_ with transition-metal amide complexes has been comparatively less studied and could represent one potential application in the synthesis of ureas and carbamates directly from CO_2_ and amines.^25^ This process has been already described using transition-metal complexes of groups 9 and 10,^14^ and the assumed mechanism is analogous to that proposed for metal hydroxides.^26^ However, to the best of our knowledge, there has not been evidence for the isolation of a gold(I) carbamate complex obtained through the insertion of CO_2_ into a Au–NH bond. To test this possibility, we exposed complex **8** to 1 bar of CO_2_ and monitored the solution by ^31^P{^1^H} NMR spectroscopy, which revealed the immediate appearance of a new singlet at 4.2 ppm (ca. 40% conversion). Based on the spectroscopic characterization of the related hydrogen carbonate gold(I) complex **3** (^31^P{^1^H} δ 3.6), we attribute this new species to the targeted gold(I) carbamate complex **9**. The conversion toward **9** remained unchanged even after 18 h under CO_2_ atmosphere, suggesting an equilibrium between species **8** and **9**. In accordance, we observed that compound **9** readily releases CO_2_ either by exposing the reaction mixture to air or by applying vacuum, leading back to the quantitative formation of complex **8** and demonstrating the reversibility of the reaction. This is unusual in the context of CO_2_ insertion chemistry in transition-metal systems, which tend to proceed in most cases through irreversible reactions.^27^

In order to reach full conversion, a solution of complex **8** in an NMR pressure tube in C_6_D_6_ was charged with 8 bar of CO_2_ affording complex **9** as the only discernible species (Scheme 2b), which was characterized *in situ*. ^1^H NMR spectrum showed the characteristic NH singlet at 5.58 ppm and the carbonyl signal was easily identified in the ^13^C{^1^H} NMR spectrum at 157.8 ppm. Unfortunately, all attempts to obtain single crystals for X-ray analysis were unsuccessful due to CO_2_ extrusion and formation of crystals of compound **8**.

**Figure 4:**
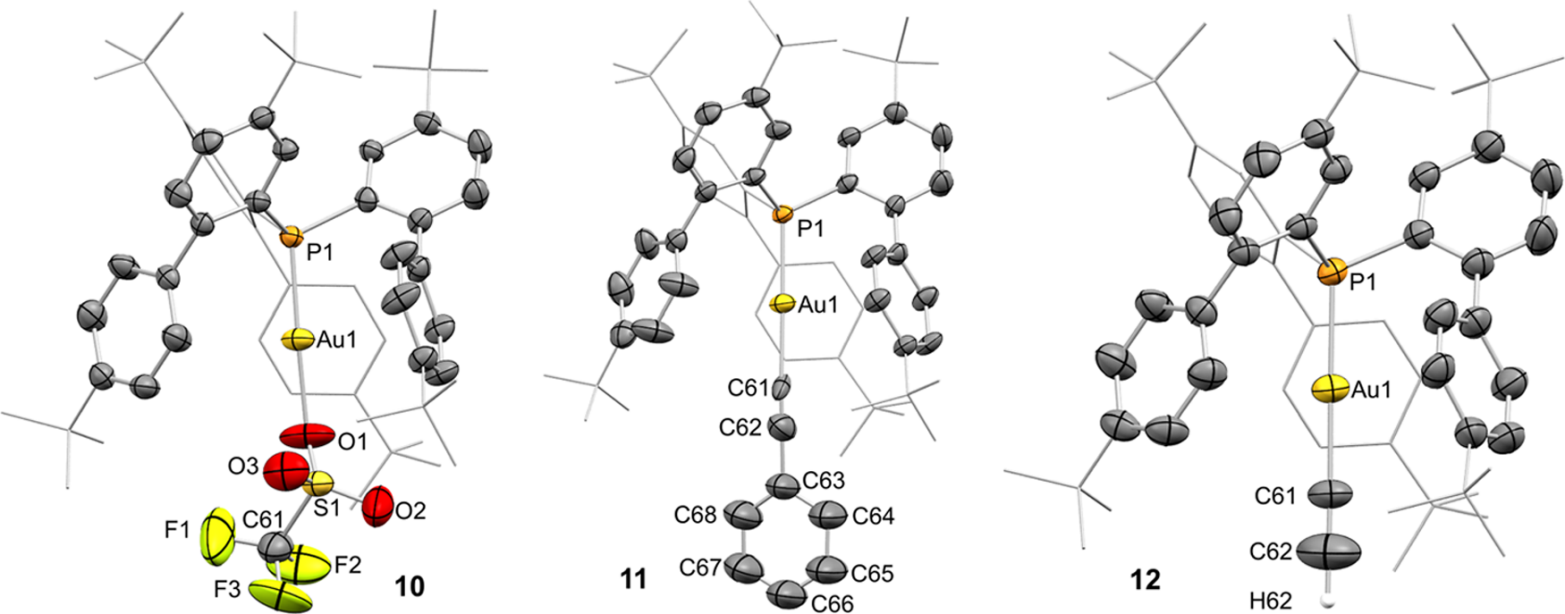
ORTEP representation of complexes **10**, **11**, and **12**. Thermal ellipsoids are set at 50% probability. Solvent molecules and most hydrogen atoms are excluded for clarity, while *tert*-butyl groups and one biaryl fragment are represented in wireframe format. Selected bond lengths (Å) and angles (°): compound **10**, P1–Au1, 2.1119(15); Au1–O1, 2.102(6); P1–Au1–O1, 178.6(2); compound **11**, P1–Au1, 2.2806(16); Au1–C61, 2.123(9); P1–Au1–C1, 174.67(8); compound **12**, P1–Au1, 2.2732(11); Au1–C61, 2.044(5); P1–Au1–C1, 179.3(2).

**Scheme 3:**
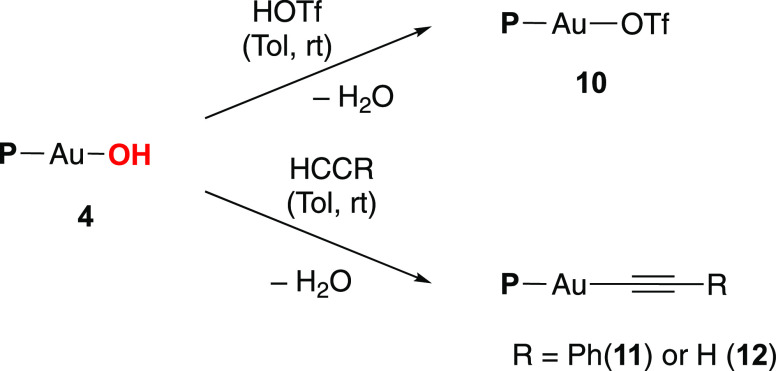
Synthesis of Gold Compounds **10**–**12** by Protonolysis Reactions from Complex **4**

We have demonstrated the basic nature of the OH unit in complex **4**, which facilitates the protonolysis with aniline to generate the gold(I) amide complex **8**. In this regard, we have also seen that complex **4** reacts with traces of acid in chlorinated deuterated solvents such as dichloromethane and chloroform leading to partial regeneration of complex **1**. To conclude this work, and based on this basic nature of complex **4**, we decided to study some additional reactivity toward other molecules containing acidic protons. First, considering that most gold-centered catalysis relies on the generation of a cationic gold(I) center with a vacant site and that this is usually achieved by using silver-based salts with weakly coordinating anions (e.g., trifluoromethanesulfonate, [OTf]^−^),^28^ we sought of interest to access such a complex without the need of using silver salts. This is particularly important because in not few cases the presence of silver has a direct influence on the catalytic outcome, the so-called “silver effect.”^29^ Therefore, we examined the protonolysis reaction of complex **4** with HOTf at room temperature, which readily generates complex (P-ligand)Au(OTf) **10** as an air-stable complex (Scheme 3). Thus, this silver-free route to generate complex **10** represents a valuable approach to prepare phosphine-ligated gold(I) catalysts.^30^ Single-crystal X-ray diffraction analysis for complex **10** showed a neutral linear gold(I) complex with a P1–Au1–O1 angle of 175.0(2)° and a Au–O bond length of 2.112(2) Å, clearly elongated compared to compounds **3** and **4**, as anticipated for a weakly coordinating triflic anion (Figure 4).

Finally, we investigated the protonolysis reaction of complex **4** with two alkynes. Treating **4** with phenylacetylene in toluene at room temperature afforded the gold alkynyl complex **11** (Scheme 3), proving a valuable mild route synthesis for gold acetylide complexes, as already demonstrated by Nolan for NHC-gold(I) alkynyl complexes.^31^ Similarly, the reaction of complex **4** with 1 bar of acetylene generated the acetylide complex **12** (Scheme 3). Interestingly, the synthesis of the terminal acetylide complex **12** is performed directly from acetylene without any external base, in contrast to previously reported terminal acetylide complexes in which external bases^32^ or Grignard reagents^5,33^ are needed. The ^13^C{^1^H} NMR spectrum of complexes **11** and **12** showed two characteristic doublets for the Au–CCR moiety (R = Ph(**11**) or H(**12**)): one at 135.3 and 126.2 with a coupling constant of ^2^*J*_CP_ ∼ 60 Hz for the *C*CR carbon signal and another at 99.8 and 86.1 ppm with a coupling constant of ^3^*J*_CP_ = 25 Hz corresponding to the C*C*R, respectively. Single-crystal X-ray diffraction analysis for both complexes **11** and **12** revealed equivalent linear geometries with P1–Au1–C61 angles of 178.6(2) and 179.3(2)° (Figure 4), respectively, and with the carbon of the terminal acetylide group coordinated to the gold center in the expected σ fashion (Au–C61 bond of 2.123(8) and 2.044(5) Å, respectively).

## Conclusions

In summary, we have synthesized in high yield a monomeric linear gold(I) hydroxide complex bearing an extremely bulky phosphine and demonstrated its ability to act as a gold synthon to access a variety of unusual gold monomeric species. Notably, complex **4** is able to trap atmospheric CO_2_ through insertion into the Au–OH bond affording the unprecedented monomeric hydrogen carbonate gold complex **3**, in which the steric hindrance of the phosphine prevents the formation of dimeric or trimeric species containing bridging OCOOH^–^ or CO_3_^2–^ moieties, as observed for all prior reported examples. The gold synthon **4** has allowed us to identify the first phosphine-based monomeric gold(I) hydride complex **5**, thus filling a long-sought gap in the organometallic chemistry of Au(I).

We have also demonstrated that complex **4** has a marked basic character, reacting with very weakly acidic protons such as terminal alkynes forming monomeric gold(I) terminal acetylides **11** and **12**. In addition, complex **4** reacts with aniline to form the amido complex **8**. Complex **8** can also trap CO_2_ through reversible insertion into the Au–NH bond forming the unusual terminal gold(I) carbamate complex **9**, and also react with phenyl isocyanate to yield the gold(I) urea complex **7**.

## Experimental Section

### General Considerations

**Figure 5:**
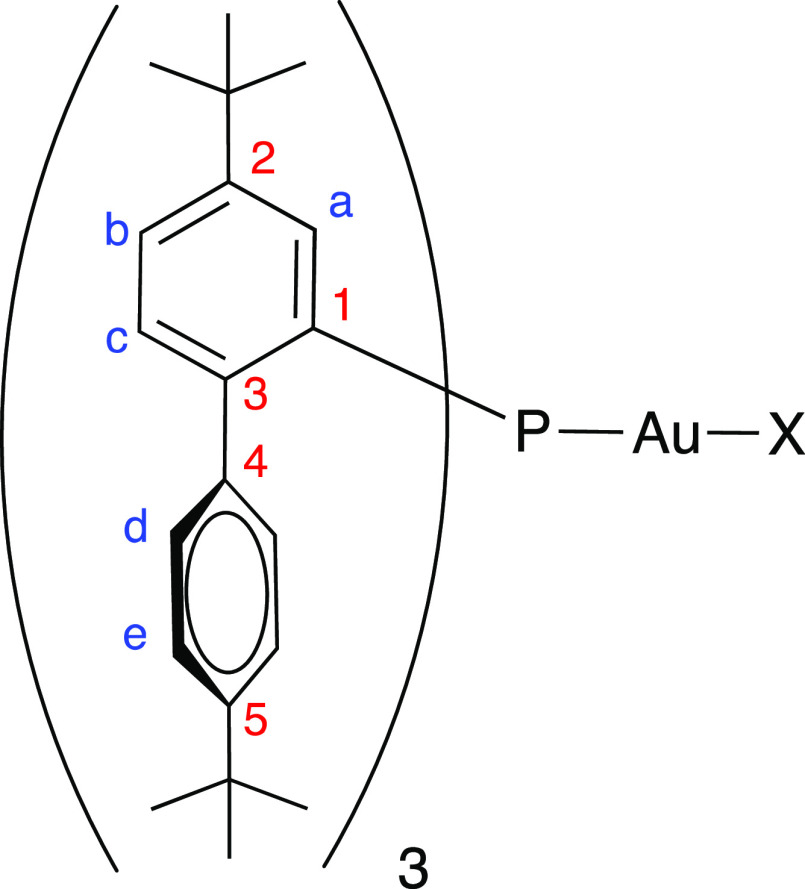
Labeling scheme used for ^1^H and ^13^C{^1^H} NMR assignments.

Unless otherwise stated, all reactions and manipulations were carried out under an atmosphere of dry argon or nitrogen using standard Schlenk techniques or in a nitrogen glovebox. Solvents were distilled under an inert atmosphere prior to use. Solution ^1^H, ^13^C, and ^31^P NMR spectra were recorded on Bruker AMX-300, DRX-400, and DRX-500 spectrometers at 298 K unless otherwise stated. Chemical shifts (δ) are expressed with a positive sign, in parts per million. ^1^H and ^13^C chemical shifts reported are referenced internally to residual protio (^1^H) or deutero (^13^C) solvent, while ^31^P chemical shifts are relative to 85% H_3_PO_4_. The following abbreviations and their combinations are used: br, broad; s, singlet; d, doublet; t, triplet; m, multiplet. The ^1^H and ^13^C resonance signals were attributed by means of two-dimensional (2D) HSQC and HMBC experiments (Figure 5). Infrared spectra were recorded with a Bruker Vector 22 spectrometer, and sample preparation was carried out in dichloromethane solution. For elemental analyses, a LECO TruSpec CHN elementary analyzer was utilized. Complex **1** was prepared according to a literature procedure.^13^ All other reagents were used as received from commercial suppliers. The yields obtained for complexes **3**, **4**, **7**, **8**, and **12** have been calculated taking into account the amount of residual pentane observed by ^1^H and ^13^C NMR spectroscopy in the samples.

#### Synthesis of Gold Complex **2**

A solution of complex **1** (51 mg, 0.05 mmol) in THF (5 mL) was heated at 50 °C in the presence of NaO^*t*^Bu (5 mL, 0.03 mmol) for 18 h. After this time, the reaction was cooled down to rt and filtered through a short pad of Celite. The solvent was removed under vacuum affording complex **2** as a colorless solid (45 mg, 82%). **Anal. calcd** for C_64_H_84_AuOP: C, 70.05; H, 7.72. Found: C, 70.24; H, 7.49. **^1^H NMR** (400 MHz, CD_2_Cl_2_, 25 °C) δ: 7.53 (d, 3H, ^3^*J*_HH_ = 8.0 Hz, H_b_), 7.40–7.22 (m, 6H, H_a_, H_c_), 7.14 (d, 6H, ^3^*J*_HH_ = 8.0 Hz, H_e_), 6.90 (d, 6H, ^3^*J*_HH_ = 8.0 Hz, H_d_), 1.28 (s, 27H, CH_3(*t*Bu3)_), 1.18 (s, 27H, CH_3(*t*Bu3)_), 0.52 (s, 9H, CH_3(O*t*Bu)_). **^13^C{^1^H} NMR** (100 MHz, CD_2_Cl_2_, 25 °C) δ: 150.4 (s, C_5_), 149.9 (d, ^3^*J*_CP_ = 8 Hz, C_2_), 145.0 (d, ^2^*J*_CP_ = 15 Hz, C_3_), 138.6 (d, ^3^*J*_CP_ = 5 Hz, C_4_), 133.4 (d, ^2^*J*_CP_ = 7 Hz, CH_a_), 130.7 (d, ^1^*J*_CP_ = 57 Hz, C_1_), 129.8 (s, CH_d_), 128.9 (s, CH_c_), 127.6 (s, CH_b_), 125.3 (s, CH_e_), 70.4 (s, C_(O*t*Bu)_), 36.7 (s, CH_3(O*t*Bu)_), 35.1 (s, C_(*t*Bu3)_), 34.9 (s, C_(*t*Bu3)_), 31.7 (s, CH_3(*t*Bu3)_), 31.4 (s, CH_3(*t*Bu3)_). **^31^P{^1^H} NMR** (162 MHz, CD_2_Cl_2_, 25 °C) δ: 12.2.

#### Synthesis of Gold Complex **3**

In a J. Young NMR tube, complex **4** (21 mg, 0.02 mmol) was dissolved in C_6_D_6_ (0.5 mL), freeze-pumped to remove the nitrogen gas, and filled with 1 bar of CO_2_. After 30 min of shaking, the solvent was removed under reduced pressure yielding complex **3** as a colorless solid (16 mg, 74%). Crystals suitable for X-ray diffraction were grown by slow evaporation of pentane into a dichloromethane solution of complex **3**. **Anal. Calcd**. for C_61_H_76_AuO_3_P: C, 67.51; H, 7.06. Found: C, 67.52; H, 7.29. ^**1**^**H NMR** (500 MHz, C_6_D_6_, 25 °C) δ: 12.28 (bs, 1H, CO_3_H), 7.50–7.28 (m, 9H, H_a_, H_e_), 7.20–7.04 (m, 12H, H_b_, H_c_, H_d_) 1.43 (s, 27H, CH_3(*t*Bu3)_), 1.09 (s, 27H, CH_3(*t*Bu3)_). ^**13**^**C{**^**1**^**H} NMR** (125 MHz, C_6_D_6_, 25 °C) δ: 163.4 (s, CO_3_H), 151.3 (s, C_5_), 149.4 (d, ^3^*J*_CP_ = 8 Hz, C_2_), 146.2 (d, ^2^*J*_CP_ = 15 Hz, C_3_), 137.6 (d, ^3^*J*_CP_ = 7 Hz, C_4_), 132.4 (d, ^3^*J*_CP_ = 9 Hz, CH_a_), 132.2 (d, ^3^*J*_CP_ = 6 Hz, CH_c_), 129.7 (d, ^1^*J*_CP_ = 62 Hz, C_1_), 129.7 (s, CH_d_), 125.9 (s, CH_c_), 124.8 (s, CH_e_), 35.0 (s, C_(*t*Bu3)_), 34.8 (s, C_(*t*Bu3)_), 31.8 (s, CH_3(*t*Bu3)_), 31.3 (s, CH_3(*t*Bu3)_). ^**31**^**P{**^**1**^**H} NMR** (121 MHz, C_6_D_6_, 25 °C) δ: 3.6. **IR cm**^–**1**^ (CH_2_Cl_2_ solution): 1618, 1453, 1330 v(CO_3_H).

#### Synthesis of Gold Complex **4**

A solution of complex **1** (102 mg, 0.10 mmol) in THF (5 mL) was subjected to microwave irradiation at 100 °C in the presence of excess CsOH·H_2_O (150 mg, 1.00 mmol) for 1 h. After this time, the solvent was removed under vacuum affording a white solid, which was then dissolved in pentane (10 mL). The suspension was filtered through a short pad of Celite, and the solvent was removed under reduced pressure yielding complex **4** as a colorless solid (85 mg, 87%). Crystals suitable for X-ray diffraction were grown by slow evaporation of a saturated pentane solution of complex **4** at −30 °C. **Anal. Calcd**. for C_60_H_76_AuOP: C, 69.21; H, 7.36. Found: C, 69.16; H, 7.41. ^**1**^**H NMR** (300 MHz, C_6_D_6_, 25 °C) δ: 7.55 (dd, 3H, ^3^*J*_HP_ = 11.0, ^4^*J*_HH_ = 2.0 Hz, H_a_), 7.40–7.20 (m, 6H, H_b_, H_c_), 7.26 (d, 6H, ^3^*J*_HH_ = 8.0 Hz, H_e_), 7.02 (d, 6H, ^3^*J*_HH_ = 8.2 Hz, H_d_), 1.34 (s, 27H, CH_3(*t*Bu3)_), 1.19 (s, 27H, CH_3(*t*Bu3)_), −1.50 (d, 1H, ^3^*J*_HP_ = 5.9 Hz, OH). ^**13**^**C{**^**1**^**H} NMR** (75 MHz, C_6_D_6_, 25 °C) δ: 151.1 (s, C_5_), 149.5 (d, ^3^*J*_CP_ = 7 Hz, C_2_), 146.0 (d, ^2^*J*_CP_ = 16 Hz, C_3_), 138.4 (d, ^3^*J*_CP_ = 6 Hz, C_4_), 132.7 (d, ^3^*J*_CP_ = 5 Hz, CH_c_), 132.2 (d, ^2^*J*_CP_ = 9 Hz, CH_a_), 131.8 (d, ^1^*J*_CP_ = 40 Hz, C_1_), 129.7 (s, CH_d_), 127.2 (d, ^4^*J*_CP_ = 2 Hz, CH_b_), 125.3 (s, CH_e_), 34.8 (s, C_(*t*Bu3)_), 34.7 (s, C_(*t*Bu3)_), 31.6 (s, CH_3(*t*Bu3)_), 31.3 (s, CH_3(*t*Bu3)_). ^**31**^**P{**^**1**^**H} NMR** (121 MHz, C_6_D_6_, 25 °C) δ: 9.1.

#### Synthesis of Gold Complex **5**

In a J. Young NMR tube, complex **4** (10 mg, 0.01 mmol) was dissolved in THF-*d*_8_, (0.4 mL) and placed at −80 °C. In parallel, a solution of HSi(EtO)_3_ (4 μL, 0.02 mmol) in THF-*d*_8_, (0.2 mL) was also placed at −80 °C. The HSi(EtO)_3_ solution was then cannulated into the gold solution affording the gold complex **5** quantitatively, which was characterized *in situ*. ^**1**^**H NMR** (500 MHz, THF-*d*_8_, −80 °C) δ: 7.57 (d, 3H, ^3^*J*_HH_ = 8.1 Hz, H_b_), 7.33 (d, 3H, ^3^*J*_HP_ = 9.7 Hz, H_a_), 7.24 (dd, 3H, ^3^*J*_HH_ = 8.1, ^4^*J*_HP_ = 5.2 Hz, H_c_), 7.13 (d, 6H, ^3^*J*_HH_ = 8.1 Hz, H_e_), 6.92 (bs, 6H, H_d_), 3.51 (d, 1H, ^2^*J*_HP_ = 214.8 Hz, Au–H),1.25 (s, 27H, CH_3(*t*Bu3)_), 1.21 (s, 27H, CH_3(*t*Bu3)_), ^**13**^**C{**^**1**^**H} NMR** (125 MHz, THF-*d*_8_, −80 °C) δ: 149.8 (s, C_5_), 149.6 (s, C_2_), 145.4 (d, ^2^*J*_CP_ = 18 Hz, C_3_), 139.1 (s, C_4_), 132.6 (s, CH_c_), 132.3 (s, CH_a_), 131.6 (d, ^1^*J*_CP_ = 40 Hz, C_1_), 129.9 (s, CH_d_), 128.6 (s, CH_b_), 126.0 (bs, CH_e_), 34.8 (s, C_(*t*Bu3)_), 34.7 (s, C_(*t*Bu3)_), 31.7 (s, CH_3(*t*Bu3)_), 31.4 (s, CH_3(*t*Bu3)_). ^**31**^**P{**^**1**^**H} NMR** (161 MHz, THF-*d*_8_, −80 °C) δ: 34.0. ^**31**^**P NMR** (161 MHz, THF-*d*_8_, −80 °C) δ: 34.0 (d, ^2^*J*_PH_ = 215 Hz)

#### Synthesis of Gold Complex **7**

##### Method A

In a Schlenk tube, complex **4** (21 mg, 0.02 mmol) was dissolved in toluene (2 mL) in the presence of phenyl isocyanate (4 μL, 0.04 mmol) and stirred at rt for 10 min. The solvent was removed under vacuum affording a white solid that was washed with pentane (3 × 5 mL) yielding complex **7** as a white solid (19 mg, 92%).

##### Method B

In a Schlenk tube, complex **8** (22 mg, 0.02 mmol) was dissolved in toluene (2 mL) in the presence of phenyl isocyanate (2 μL, 0.02 mmol) and stirred at rt until the color of the solution changed from bright yellow to clear (10 min approx.) The solvent was removed under vacuum affording a white solid that was washed with pentane (3 × 5 mL) yielding complex **7** as a colorless solid (14 mg, 69%). Crystals suitable for X-ray diffraction were grown by slow evaporation of a saturated dichloromethane solution of complex **8**. **Anal. Calcd**. for C_73_H_86_AuN_2_OP: C, 70.97; H, 7.02; N, 2.27. Found: C, 70.82; H, 7.10; N, 2.12. ^**1**^**H NMR** (500 MHz, CD_2_Cl_2_, 25 °C) δ: 7.58 (d, 3H, ^3^*J*_HH_ = 8.1, H_b_), 7.24 (d, 3H, ^3^*J*_HH_ = 11.9, H_a_), 7.21–7.12 (m, 3H, H_c_ + 8H, CH_(Ph)_), 6.96 (t, 1H, ^3^*J*_HH_ = 7.5, CH_(Ph)_), 6.83–6.81 (m, 1H, CH_(Ph)_), 6.80 (d, 6H, ^3^*J*_HH_ = 8.0 Hz, H_e_), 6.68 (d, 6H, ^3^*J*_HH_ = 8.0 Hz, H_d_), 5.58 (s, 1H, NH), 1.21 (s, 27H, CH_3(*t*Bu3)_), 1.09 (s, 27H, CH_3(*t*Bu3)_). ^**13**^**C{**^**1**^**H} NMR** (125 MHz, CD_2_Cl_2_, 25 °C) δ: 158.7 (s, CO), 151.3 (s, C_5_), 150.9 (s, C_(Ph)_), 150.6 (d, ^3^*J*_CP_ = 8 Hz, C_2_), 145.5 (d, ^2^*J*_CP_ = 17 Hz, C_3_), 142.3 (s, C_(Ph)_),138.0 (d, ^3^*J*_CP_ = 6 Hz, C_4_), 133.1 (d, ^2^*J*_CP_ = 9 Hz, CH_a_), 133.0 (d, ^3^*J*_CP_ = 6 Hz, CH_c_), 129.8 (s, CH_d_), 129.4 (d, ^1^*J*_CP_ = 60 Hz, C_1_), 128.9 (s, CH_(Ph)_), 128.6 (s, CH_(Ph)_), 128.2 (s, CH_b_), 128.2 (s, CH_(Ph)_), 125.3 (s, CH_e_), 122.6 (s, CH_(Ph)_), 119.8 (s, CH_(Ph)_), 117.3 (s, CH_(Ph)_), 35.2 (s, C_(*t*Bu3)_), 34.9 (s, C_(*t*Bu3)_), 31.5 (s, CH_3(*t*Bu3)_), 31.4 (s, CH_3(*t*Bu3)_). ^**31**^**P{**^**1**^**H} NMR** (162 MHz, CD_2_Cl_2_, 25 °C) δ: 11.2.

#### Synthesis of Gold Complex **8**

In a Schlenk tube, complex **4** (21 mg, 0.02 mmol) was dissolved in toluene (2 mL) in the presence of aniline (3 μL, 0.03 mmol) and stirred at rt until the solution became bright yellow (10 min). The solvent was removed under vacuum affording a pale yellow solid that was washed with pentane (3 × 5 mL) yielding complex **8** as a yellow solid (12 mg, 59%). Crystals suitable for X-ray diffraction were grown by slow evaporation of pentane into a benzene solution of complex **8**. **Anal. Calcd**. for C_66_H_81_AuNP: C, 71.01; H, 7.33; N, 1.25. Found: C, 70.90; H, 7.56; N, 1.08. ^**1**^**H NMR** (400 MHz, C_6_D_6_, 25 °C) δ: 7.58 (dd, 3H, ^3^*J*_HH_ = 11.1, ^4^*J*_HP_ = 1.9 Hz, H_a_), 7.34–7.25 (m, 6H, H_b_, H_c_), 7.24–7.22 (m, 2H, CH_(Ph)_), 7.20 (d, 6H, ^3^*J*_HH_ = 8.1 Hz, H_e_), 7.07 (d, 6H, ^3^*J*_HH_ = 8.1 Hz, H_d_), 6.60 (tt, 1H, ^3^*J*_HH_ = 7.3, ^4^*J*_HH_ = 1.1 Hz, CH_(Ph)_), 6.36 (d, 2H, ^3^*J*_HH_ = 7.3 Hz, CH_(Ph)_), 2.78 (s, 1H, NH), 1.23 (s, 27H, CH_3(*t*Bu3)_), 1.18 (s, 27H, CH_3(*t*Bu3)_). ^**13**^**C{**^**1**^**H} NMR** (100 MHz, C_6_D_6_, 25 °C) δ: 159.5 (s, C_(Ph)_), 150.8 (s, C_5_), 149.7 (d, ^3^*J*_CP_ = 7 Hz, C_2_), 145.9 (d, ^2^*J*_CP_ = 17 Hz, C_3_), 138.4 (d, ^3^*J*_CP_ = 6 Hz, C_4_), 133.0 (d, ^3^*J*_CP_ = 4 Hz, CH_c_), 132.7 (d, ^2^*J*_CP_ = 9 Hz, CH_a_), 131.4 (d, ^1^*J*_CP_ = 58 Hz, C_1_), 129.6 (s, CH_d_), 128.7 (s, CH_(Ph)_), 127.5 (s, CH_b_), 125.4 (s, CH_e_),116.4 (s, CH_(Ph)_), 110.9 (s, CH_(Ph)_), 34.8 (s, C_(*t*Bu3)_), 34.6 (s, C_(*t*Bu3)_), 31.4 (s, CH_3(*t*Bu3)_), 31.3 (s, CH_3(*t*Bu3)_). ^**31**^**P{**^**1**^**H} NMR** (162 MHz, C_6_D_6_, 25 °C) δ: 16.1.

#### Synthesis of Gold Complex **9**

In a J. Young NMR tube, complex **8** (22 mg, 0.02 mmol) was dissolved in C_6_D_6_ (0.5 mL), freeze-pumped to remove the nitrogen gas, and filled with 8 bar of CO_2_, affording the gold complex **9** after 10 min of shaking, which was characterized *in situ*. ^**1**^**H NMR** (400 MHz, C_6_D_6_, 25 °C) δ: 7.66 (dd, 2H, ^3^*J*_HH_ = 8.56, ^4^*J*_HH_ = 1.2, CH_(Ph)_), 7.49 (d, 3H, ^3^*J*_HH_ = 12.1, H_b_), 7.36 (d, 6H, ^3^*J*_HH_ = 7.9 Hz, H_e_), 7.31–7.24 (m, 6H, H_a_ + H_c_ + 1H, CH_(Ph)_), 7.12 (d, 6H, ^3^*J*_HH_ = 7.9 Hz, H_d_), 6.87 (tt, 1H, ^3^*J*_HH_ = 7.4, ^4^*J*_HH_ = 1.1, CH_(Ph)_), 6.35 (d, 1H, ^3^*J*_HH_ = 8.0, CH_(Ph)_), 5.58 (s, 1H, NH), 1.32 (s, 27H, CH_3(*t*Bu3)_), 1.16 (s, 27H, CH_3(*t*Bu3)_). ^**13**^**C{**^**1**^**H} NMR** (100 MHz, C_6_D_6_,, 25 °C) δ: 157.8 (s, CO), 151.0 (s, C_5_), 149.6 (d, ^3^*J*_CP_ = 8 Hz, C_2_), 146.2 (d, ^2^*J*_CP_ = 16 Hz, C_3_), 143.1 (s, C_(Ph)_), 137.7 (d, ^3^*J*_CP_ = 7 Hz, C_4_), 132.5 (s, CH_a_), 132.4 (s, CH_c_), 130.0 (d, ^1^*J*_CP_ = 62 Hz, C_1_), 129.7 (s, CH_d_), 128.9 (s, CH_b_), 128.0 (s, C_(Ph)_), 125.7 (s, CH_e_), 120.3 (s, CH_(Ph)_), 117.6 (s, CH_(Ph)_), 115.0 (s, CH_(Ph)_), 34.8 (s, C_(*t*Bu3)_), 34.7 (s, C_(*t*Bu3)_), 31.4 (s, CH_3(*t*Bu3)_), 31.2 (s, CH_3(*t*Bu3)_). ^**31**^**P{**^**1**^**H} NMR** (162 MHz, C_6_D_6_, 25 °C) δ: 4.2.

#### Synthesis of Gold Complex **10**

In a Schlenk tube, complex **4** (20 mg, 0.02 mmol) was dissolved in toluene (2 mL). Trifluoromethanesulfonic acid (3 μL, 0.03 mmol) was added, and the reaction was stirred for 30 min at rt. The solvent was removed under reduced pressure affording a white crude solid that was washed with pentane (3 × 2 mL) to yield complex **10** as a colorless solid (20 mg, 90%). Crystals suitable for X-ray diffraction were grown by slow evaporation of pentane into a dichloromethane solution of complex **10**. **Anal. Calcd**. for C_61_H_75_AuF_3_O_3_PS: C, 62.45; H, 6.44; S 2,73. Found: C, 62.54; H 6, 45; S, 2.67. ^**1**^**H NMR** (500 MHz, CD_2_Cl_2_, 25 °C) δ: 7.60 (dt, 3H, ^3^*J*_HH_ = 8.0, ^4^*J*_HH_ = 1.8, Hz, H_b_), 7.28–7.21 (m, 6H, H_a_, H_c_), 7.18 (d, 6H, ^3^*J*_HH_ = 7.9 Hz, H_e_), 6.78 (d, 6H, ^3^*J*_HH_ = 7.9 Hz, H_d_), 1.27 (s, 27H, CH_3(*t*Bu3)_), 1.22 (s, 27H, CH_3(*t*Bu3)_). ^**13**^**C{**^**1**^**H} NMR** (125 MHz, CD_2_Cl_2_, 25 °C) δ: 151.6 (s, C_5_), 150.4 (d, ^3^*J*_CP_ = 8 Hz, C_2_), 145.4 (d, ^2^*J*_CP_ = 14 Hz, C_3_), 137.6 (d, ^3^*J*_CP_ = 14 Hz, C_4_), 132.8 (d, ^3^*J*_CP_ = 9 Hz, CH_c_),, 132.5 (d, ^2^*J*_CP_ = 7 Hz, CH_a_), 129.5 (s, CH_d_), 128.9 (s, CH_b_), 127.4 (d, ^1^*J*_CP_ = 64 Hz, C_1_), 125.7 (s, CH_e_), 125.0 (s CH_(Ph)_), 35.2 (s, C_(*t*Bu3)_), 35.2 (s, C_(*t*Bu3)_), 31.4 (s, 2 × CH_3(*t*Bu3)_). ^**31**^**P{**^**1**^**H} NMR** (202 MHz, C_6_D_6_, 25 °C) δ: 0.1.

#### Synthesis of Gold Complex **11**

In a Schlenk tube, complex **4** (21 mg, 0.02 mmol) was dissolved in toluene (2 mL). Phenylacetylene (3 μL, 0.03 mmol) was added and the reaction was stirred for 30 min at rt. The solvent was removed under reduced pressure affording a white crude solid that was washed with pentane (3 × 2 mL) at 0 °C yielding complex **11** as a colorless solid (16 mg, 80%). Crystals suitable for X-ray diffraction were grown by slow evaporation of pentane into a dichloromethane solution of complex **11**. **Anal. Calcd**. for C_68_H_80_AuP: C, 72.58; H, 7.17. Found: C, 72.41; H, 7.44. ^**1**^**H NMR** (300 MHz, CD_2_Cl_2_, 25 °C) δ: 7.55 (dt, 3H, ^3^*J*_HH_ = 8.0, ^4^*J*_HH_ = 1.6, Hz, H_b_), 7.36–7.23 (m, 6H, H_a_, H_c_), 7.16–7.12 (m, 4H, CH_(Ph)_), 7.12 (d, 6H, ^3^*J*_HH_ = 8.2 Hz, H_e_), 7.06 (dd, 1H,, ^3^*J*_HH_ = 5.7, ^4^*J*_HH_ = 2.9, Hz, CH_(Ph)_), 6.71 (d, 6H, ^3^*J*_HH_ = 8.2 Hz, H_d_), 1.25 (s, 27H, CH_3(*t*Bu3)_), 1.22 (s, 27H, CH_3(*t*Bu3)_). ^**13**^**C{**^**1**^**H} NMR** (125 MHz, CD_2_Cl_2_, 25 °C) δ: 151.0 (s, C_5_), 150.3 (d, ^3^*J*_CP_ = 7 Hz, C_2_), 145.4 (d, ^2^*J*_CP_ = 17 Hz, C_3_), 138.2 (d, ^3^*J*_CP_ = 6 Hz, C_4_), 133.4 (d, ^3^*J*_CP_ = 4 Hz, CH_c_), 132.2 (s, CH_(Ph)_),132.1 (d, ^2^*J*_CP_ = 8 Hz, CH_a_), 132.0 (s, CH_(Ph)_), 131.6 (d, ^1^*J*_CP_ = 52 Hz, C_1_), 129.7 (s, CH_d_), 128.9 (d, ^4^*J*_CP_ = 3 Hz, C_(Ph)_), 128.0 (s, CH_(Ph)_), 127.6 (d, ^4^*J*_CP_ = 2 Hz, CH_b_), 125.6 (s, CH_e_), 125.0 (s CH_(Ph)_), 135.3 (d, ^2^*J*_CP_ = 54 Hz, *C*CPh), 99.8 (d, ^3^*J*_CP_ = 25 Hz, C*C*Ph), 35.1 (s, C_(*t*Bu3)_), 34.9 (s, C_(*t*Bu3)_), 31.6 (s, CH_3(*t*Bu3)_), 31.5 (s, CH_3(*t*Bu3)_). ^**31**^**P{**^**1**^**H} NMR** (202 MHz, C_6_D_6_, 25 °C) δ: 24.4.

#### Synthesis of Gold Complex **12**

In a J. Young NMR tube, complex **4** (21 mg, 0.02 mmol) was dissolved in C_6_D_6_ (0.5 mL), freeze-pumped to remove the nitrogen gas, and filled with 1 bar of acetylene. After 30 min of shaking, the solvent was removed under reduced pressure yielding complex **12** as a colorless solid (15 mg, 67%). Crystals suitable for X-ray diffraction were grown by slow evaporation of pentane into a dichloromethane solution of complex **12**. ^**1**^**H NMR** (500 MHz, CD_2_Cl_2_, 25 °C) δ: 7.53 (d, 3H, ^3^*J*_HH_ = 8.1 Hz, H_b_), 7.30–7.24 (m, 6H, H_a_, H_c_), 7.11 (d, 6H, ^3^*J*_HH_ = 7.8 Hz, H_e_), 6.71 (d, 6H, ^3^*J*_HH_ = 7.8 Hz, H_d_), 1.29 (s, 27H, CH_3(*t*Bu3)_), 1.20 (s, 27H, CH_3(*t*Bu3)_), 0.83 (d, 1H, ^4^*J*_HP_ = 5.8 Hz, C≡CH). ^**13**^**C{**^**1**^**H} NMR** (125 MHz, CD_2_Cl_2_, 25 °C) δ: 150.9 (s, C_5_), 150.2 (d, ^3^*J*_CP_ = 7 Hz, C_2_), 145.3 (d, ^2^*J*_CP_ = 17 Hz, C_3_), 138.2 (d, ^3^*J*_CP_ = 6 Hz, C_4_), 133.3 (d, ^3^*J*_CP_ = 4 Hz, CH_c_), 132.1 (d, ^2^*J*_CP_ = 8 Hz, CH_a_), 131.4 (d, ^1^*J*_CP_ = 52 Hz, C_1_), 129.6 (s, CH_d_), 127.6 (d, ^4^*J*_CP_ = 2 Hz, CH_b_), 126.2 (d, ^2^*J*_CP_ = 62 Hz, *C*CH), 125.6 (s, CH_e_), 86.1 (d, ^3^*J*_CP_ = 26 Hz, C*C*H), 35.1 (s, C_(*t*Bu3)_), 34.9 (s, C_(*t*Bu3)_), 31.7 (s, CH_3(*t*Bu3)_), 31.5 (s, CH_3(*t*Bu3)_). ^**31**^**P{**^**1**^**H} NMR** (202 MHz, C_6_D_6_, 25 °C) δ: 24.3.

## Supporting Information Available

NMR spectra, X-ray structural data, and computational details (PDF)
