# Supplementary material for: A Cavity-Shaped Gold(I) Fragment Enables CO2 Insertion into Au–OH and Au–NH Bonds
Source: Inorg Chem. 2023 Jun 27;62(27):10582–91. doi: 10.1021/acs.inorgchem.3c00751 (PMC10336967; doi:10.1021/acs.inorgchem.3c00751)
Supplement: Supplementary file 1 — ic3c00751_si_001.pdf [file ic3c00751_si_001.pdf]

A Cavity-Shaped Gold(I) Fragment Enables CO_2 Insertion into Au–OH and Au–NH Bonds

Miquel Navarro*, Markus Holzapfel,[‡] Jesús Campos*

[†] Instituto de Investigaciones Químicas (IIQ), Departamento de Química Inorgánica and Centro de Innovación en Química Avanzada (ORFEO−CINQA). Consejo Superior de Investigaciones Científicas (CSIC) and University of Sevilla, 41092 Sevilla, Spain

[‡] *Permanent Address* – Institute of Applied Chemistry, Department of Science and Technology. IMC University of Applied Sciences Krems. 3500, Austria.

E-mail: miquel.navarro@iiq.csic.es; jesus.campos@iiq.csic.es

1. Summary of reactivity S2

2. NMR spectroscopic experiments.......... S3

3. IR spectroscopic experiments.......... S21

4. Crystal structure determinations.......... S22

5. Computational details.......... S27

6. References S89

1. Summary of reactivity

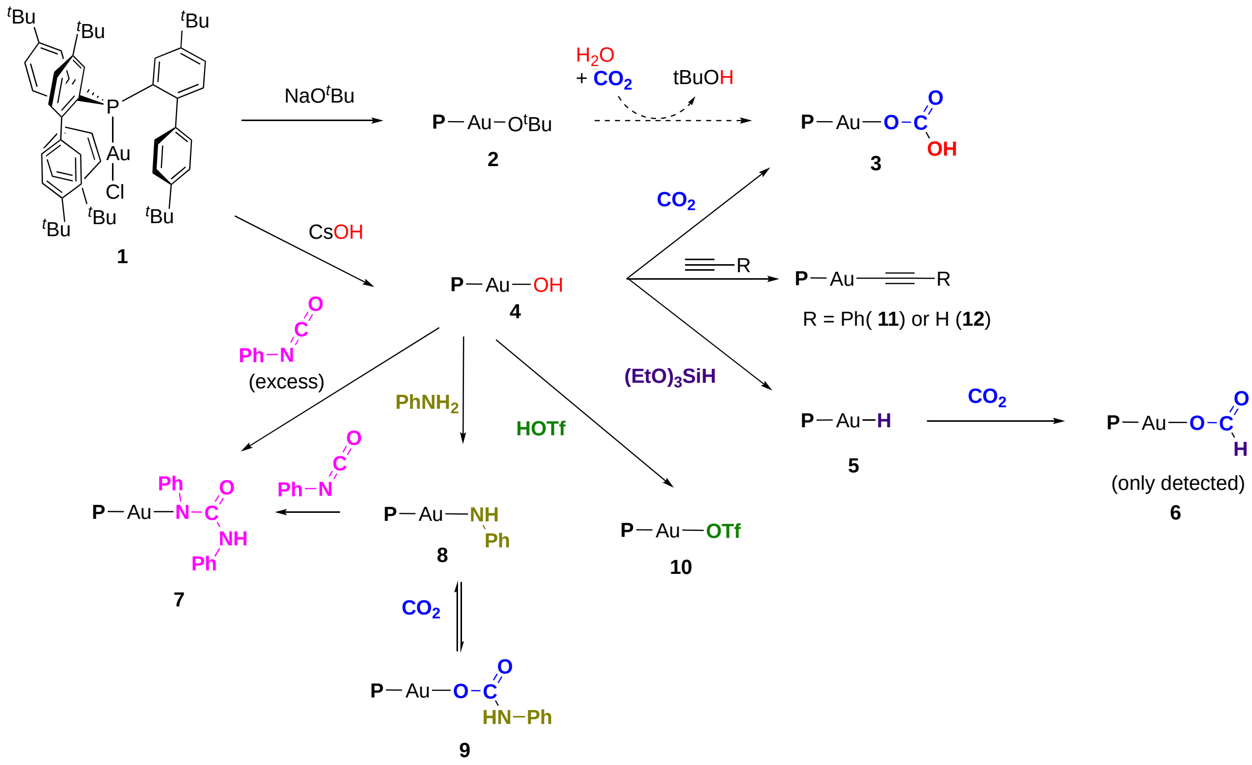

Scheme S1. Reactivity derived from gold(I) hydroxide synthon **4**.

2. NMR spectroscopic experiments

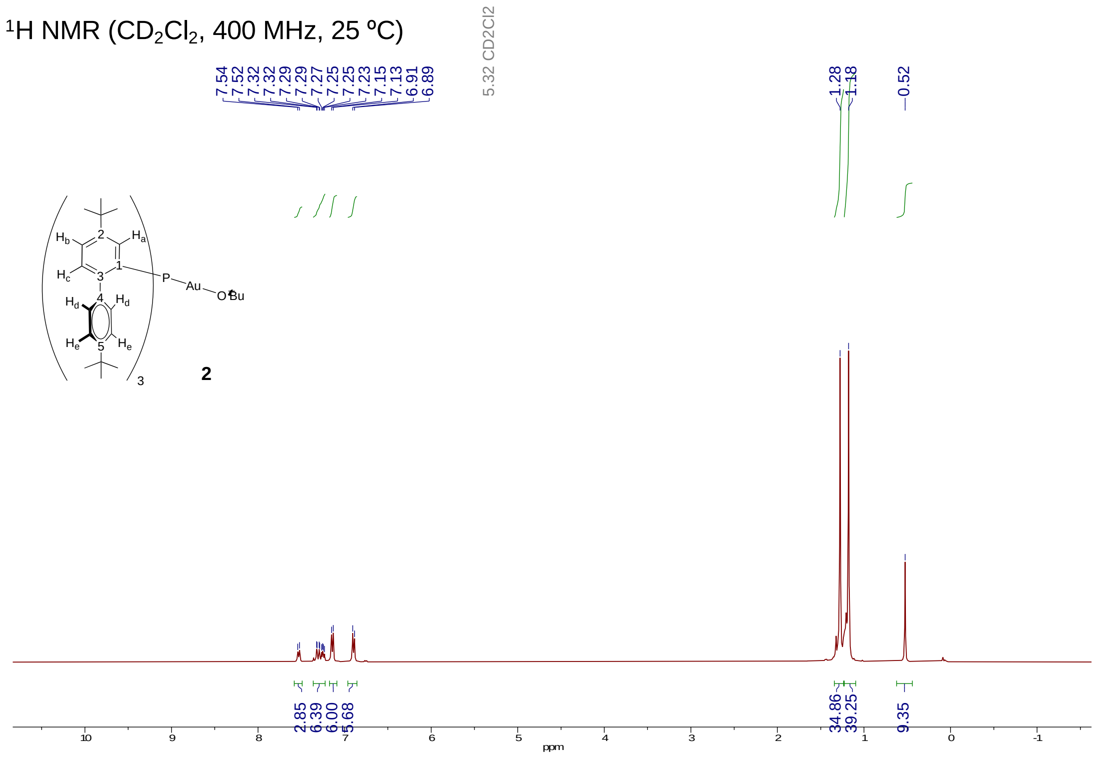

Figure S1. ^{1}H NMR of complex **2**.

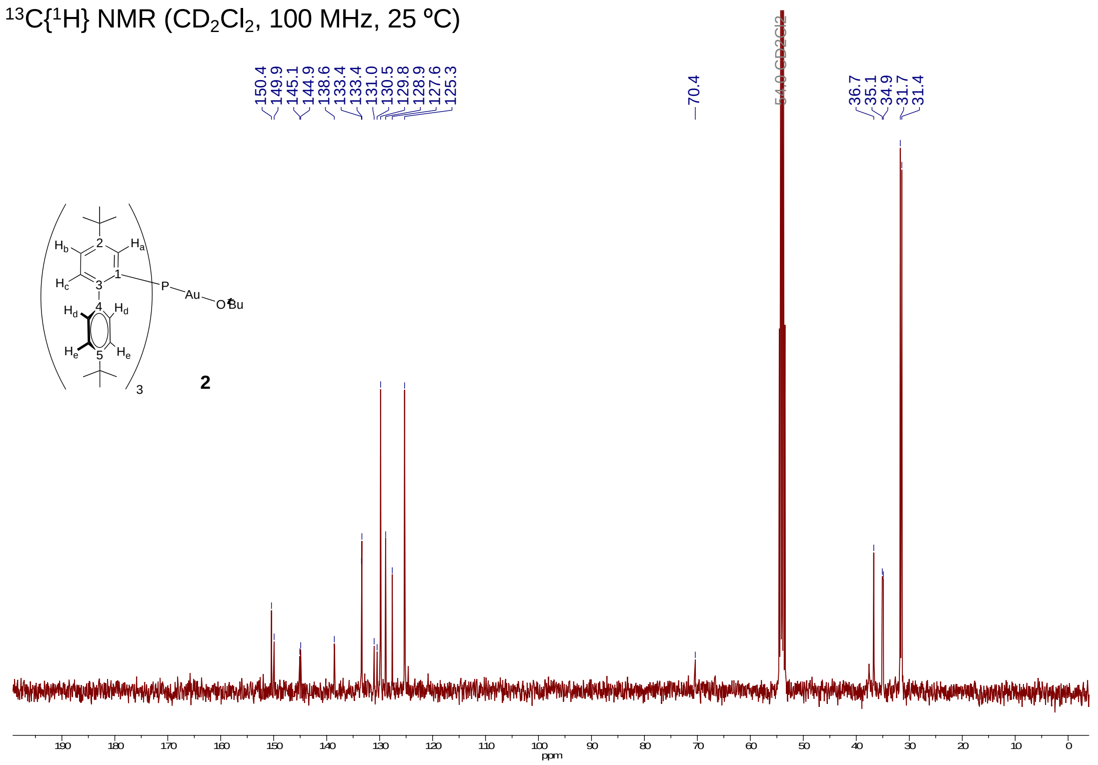

Figure S2. $^{13}C\{^{1}H\}$ NMR of complex **2**.

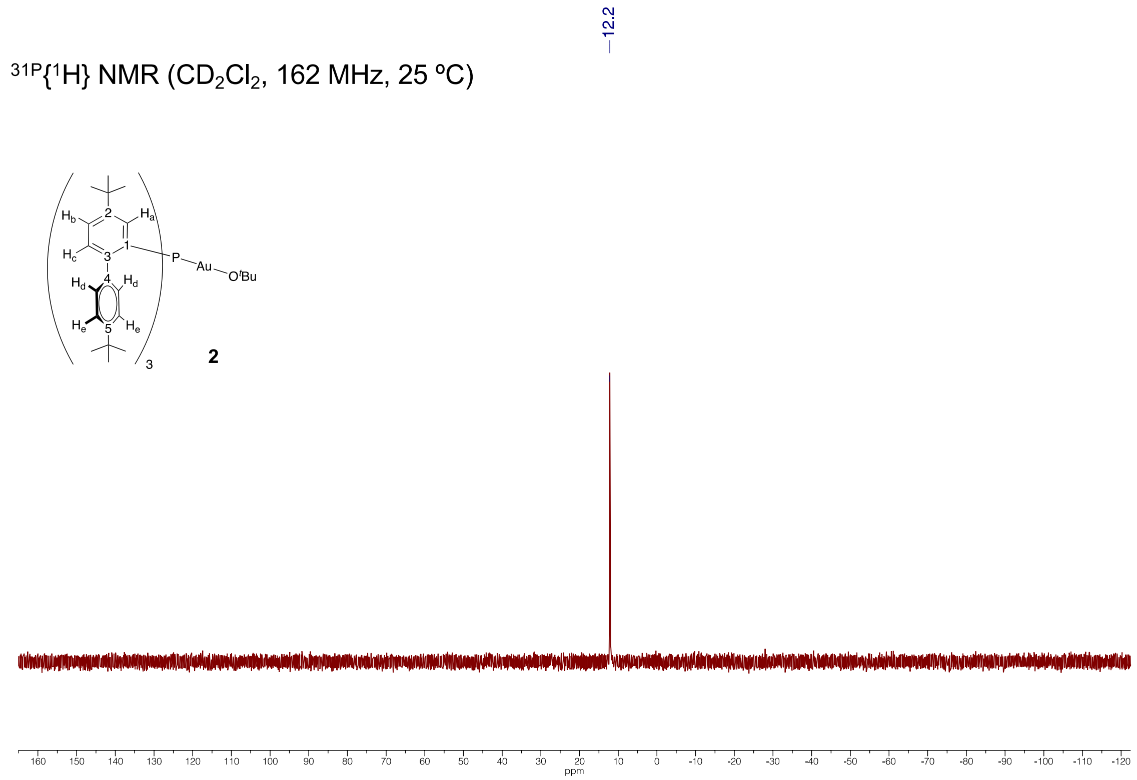

Figure S3. $^{31}P\{^1H\}$ NMR of complex **2**.

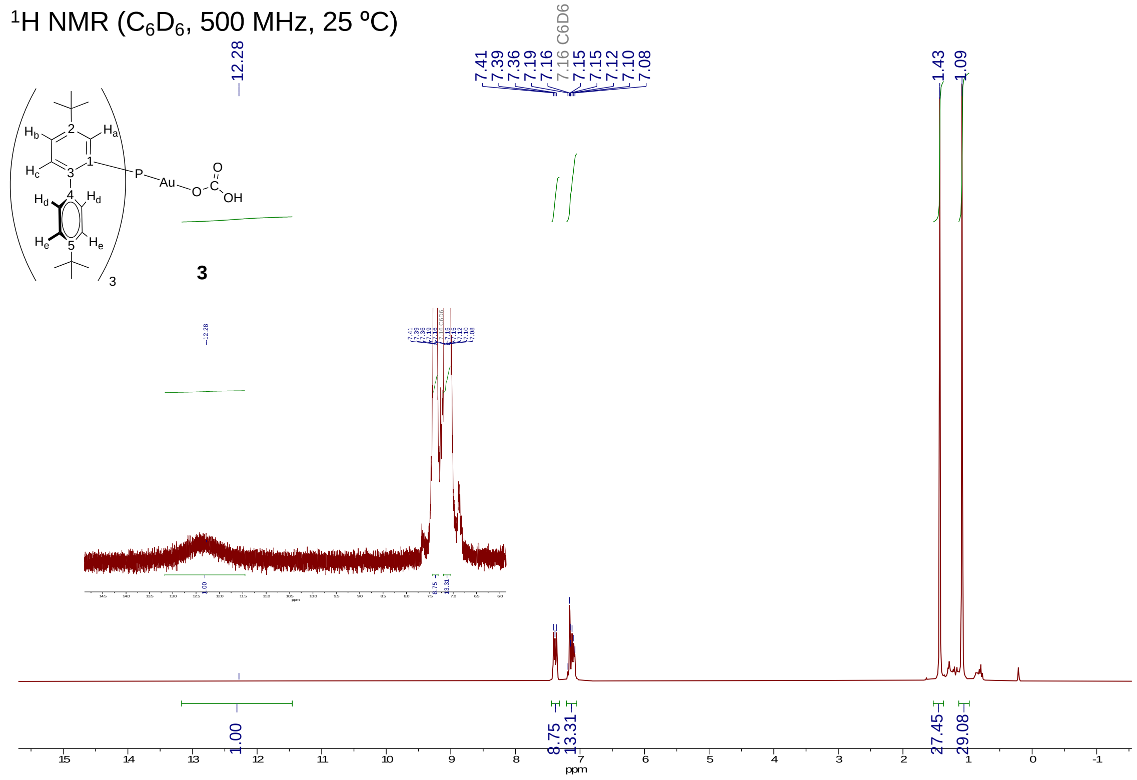

Figure S4. ^1H NMR of complex **3** with inset of the COOH proton region.

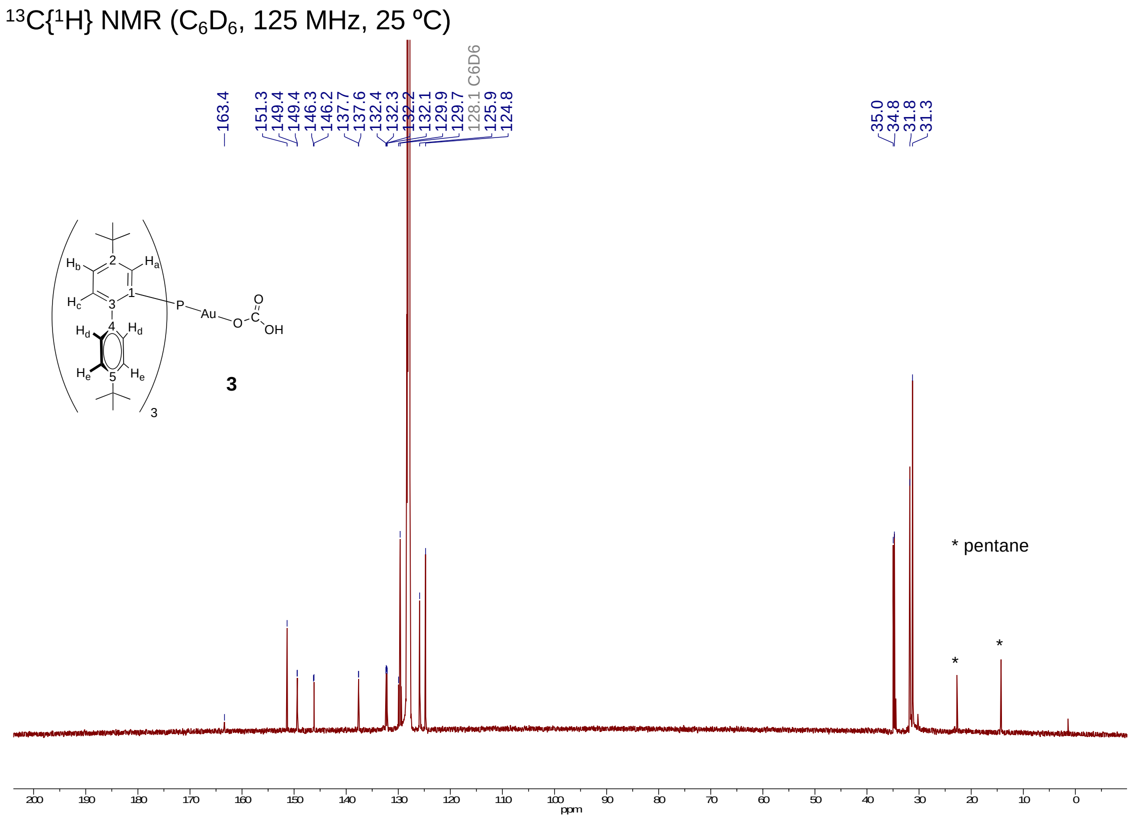

Figure S5. $^{13}C\{^1H\}$ NMR of complex **3**.

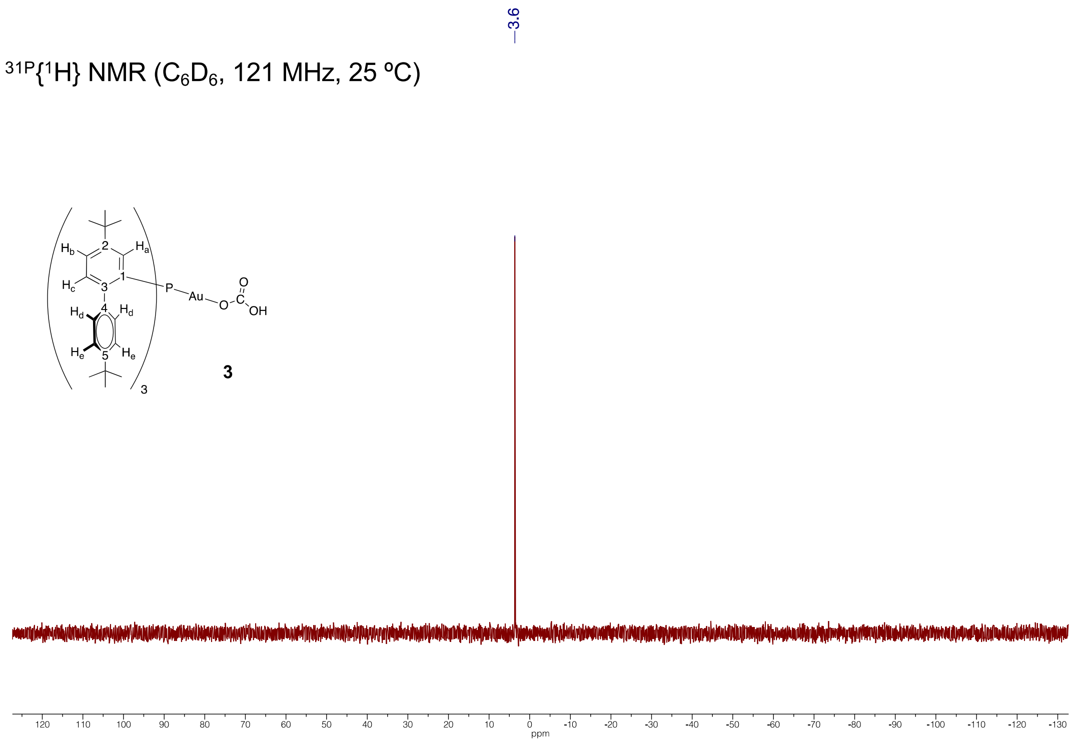

Figure S6. $^{31}P\{^1H\}$ NMR of complex **3**.

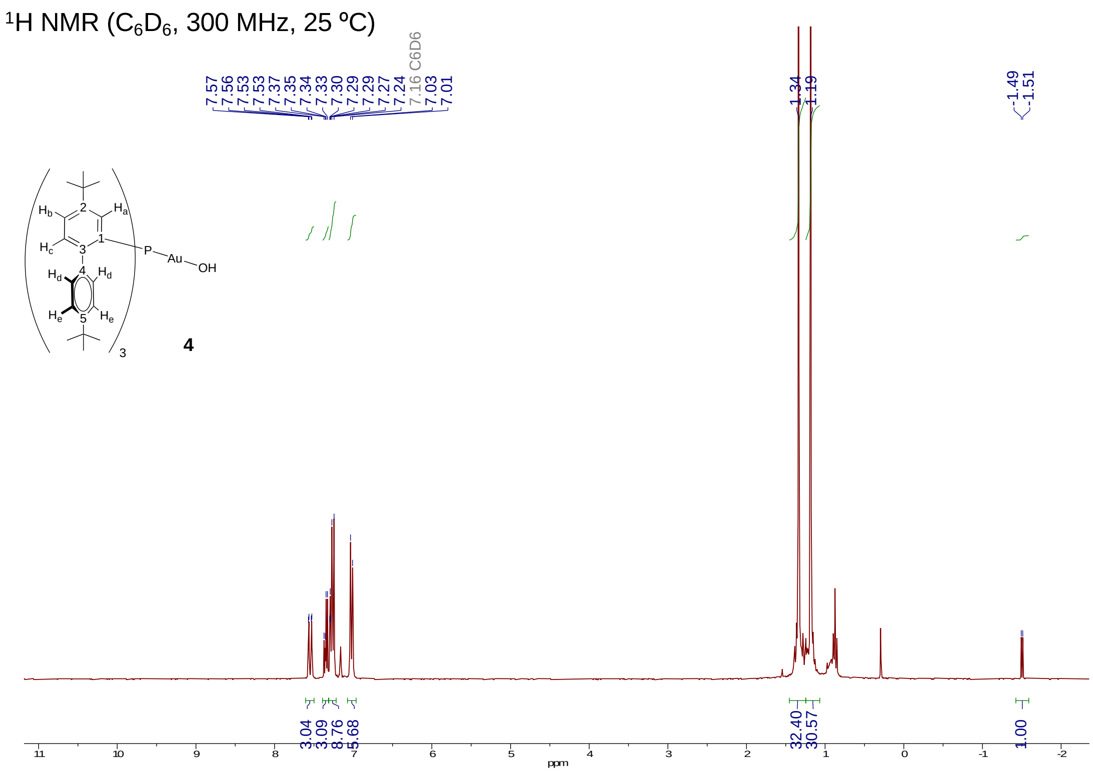

Figure S7. ^{1}H NMR of complex **4**.

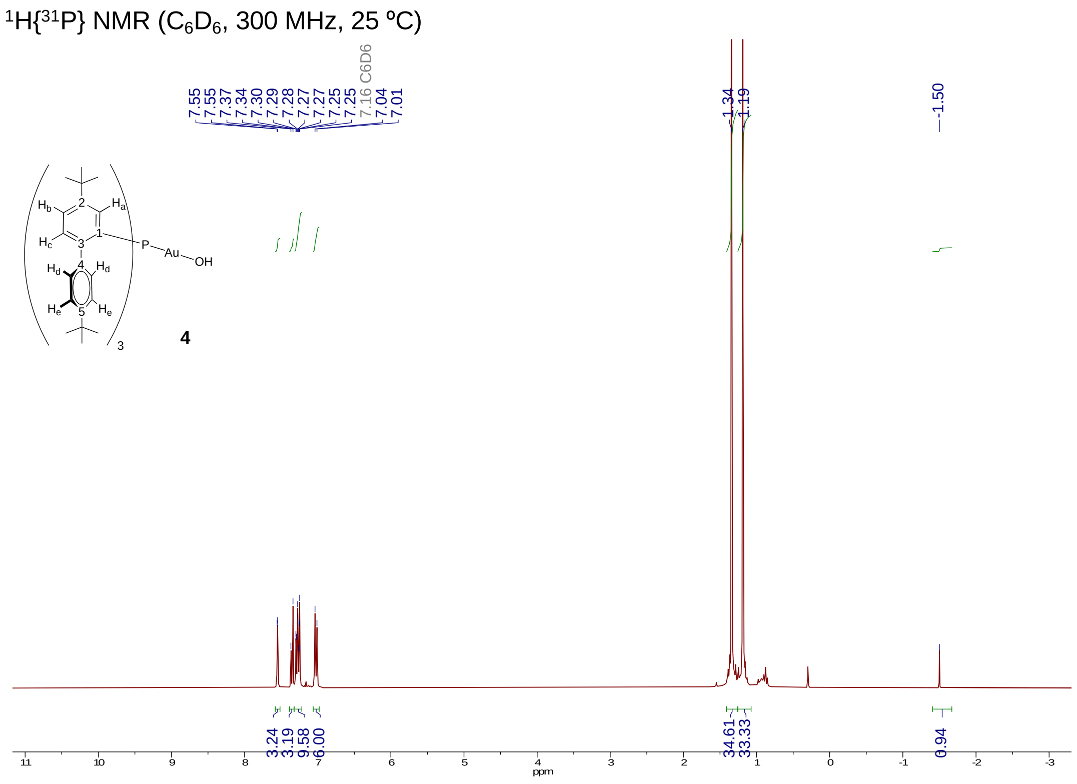

Figure S8. $^{1}H\{^{31}P\}$ NMR of complex **4**.

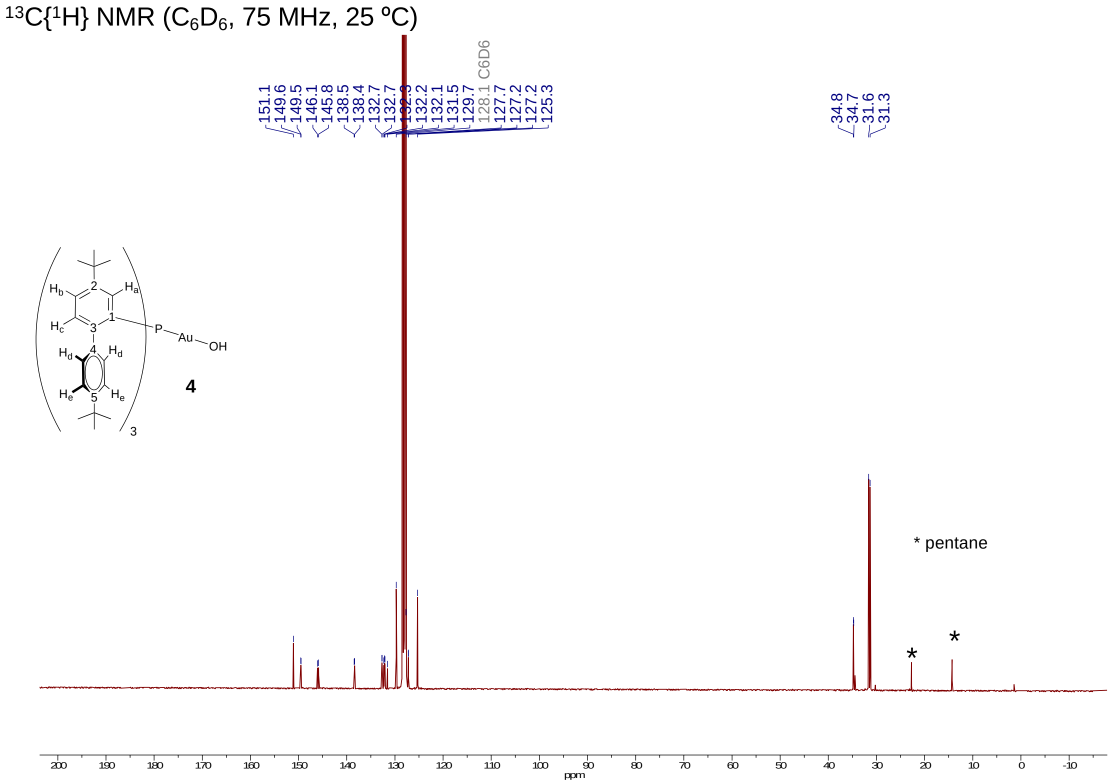

Figure S9. $^{13}C\{^1H\}$ NMR of complex **4**.

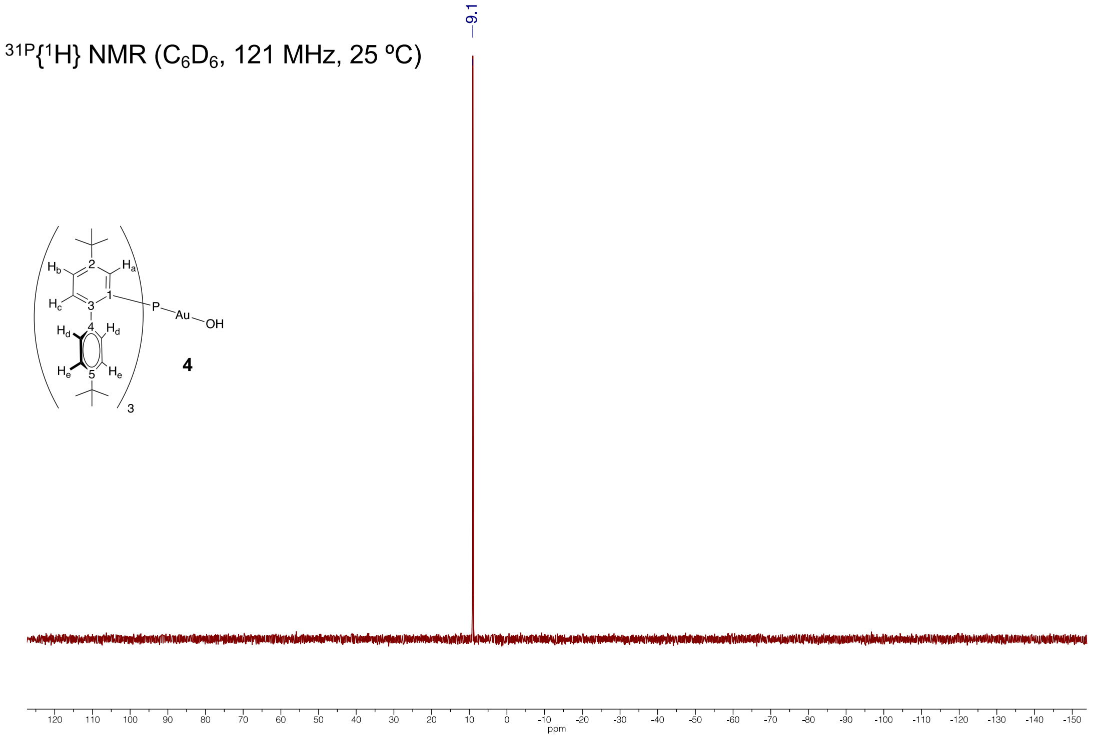

Figure S10. $^{31}P\{^1H\}$ NMR of complex **4**.

^1H NMR (THF-d_6, 500 MHz, –80 ºC)

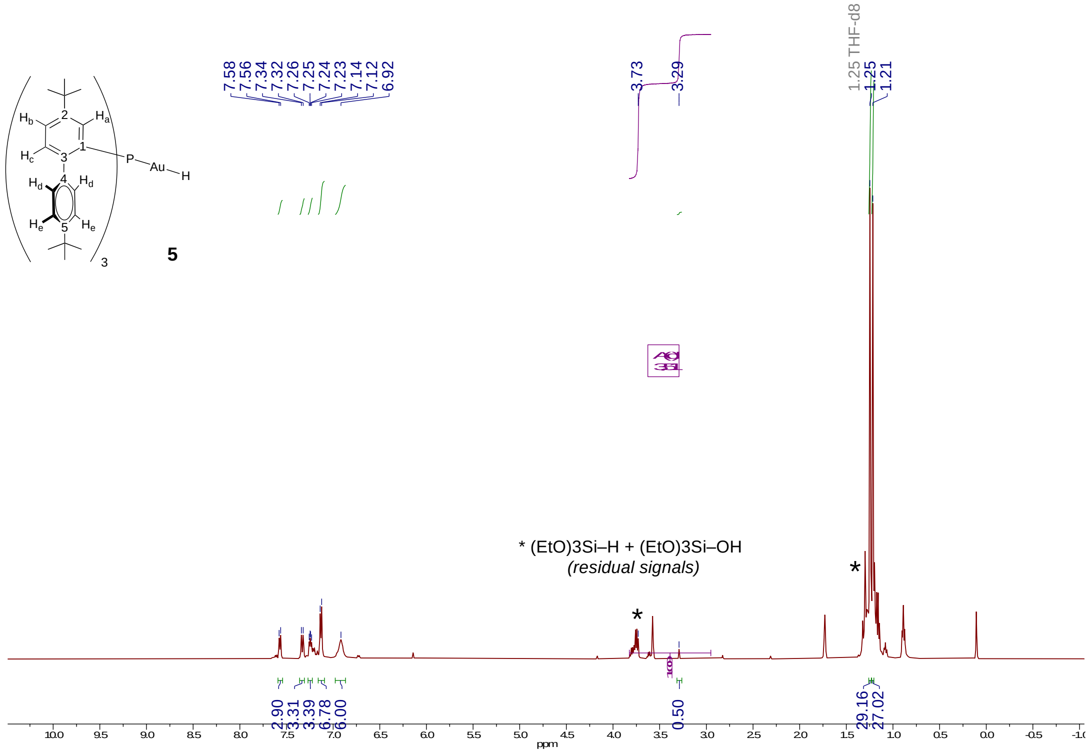

Figure S11. ^1H NMR of complex **5** at –80 ºC.

$^1H\{^{31}P\}$ NMR (THF-d_6, 500 MHz, –80 ºC)

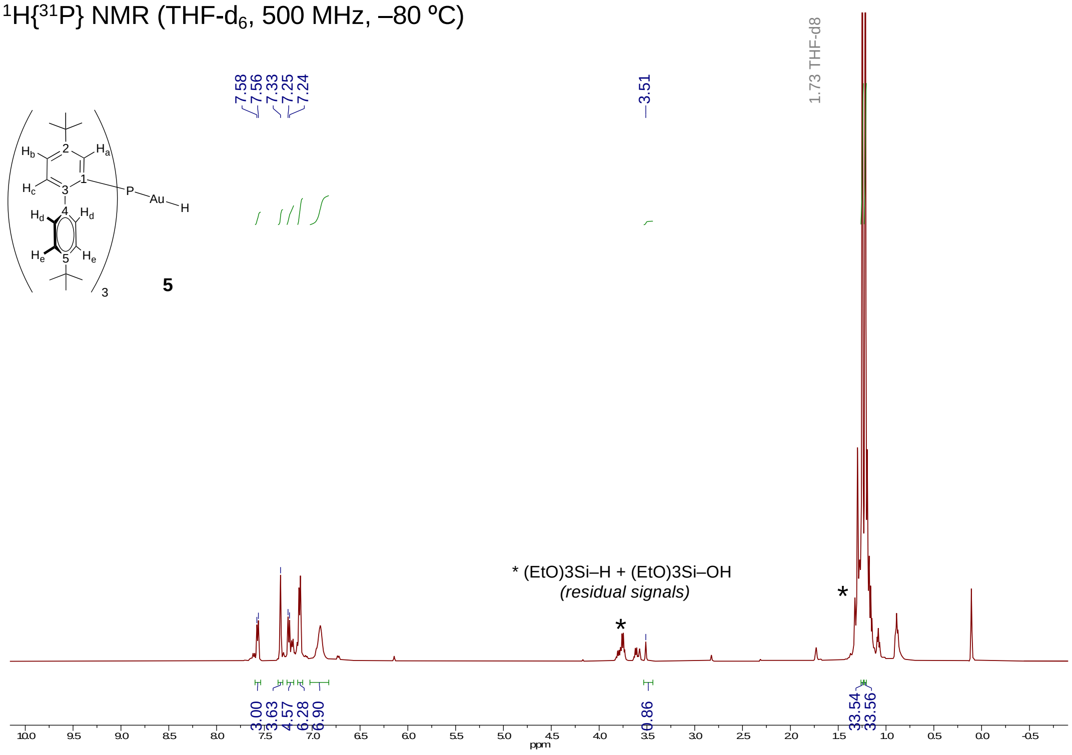

Figure S12. $^1H\{31P\}$ NMR of complex **5** at –80 ºC.

$^{13}C\{^1H\}$ NMR (THF-d_6, 125 MHz, –80 ºC)

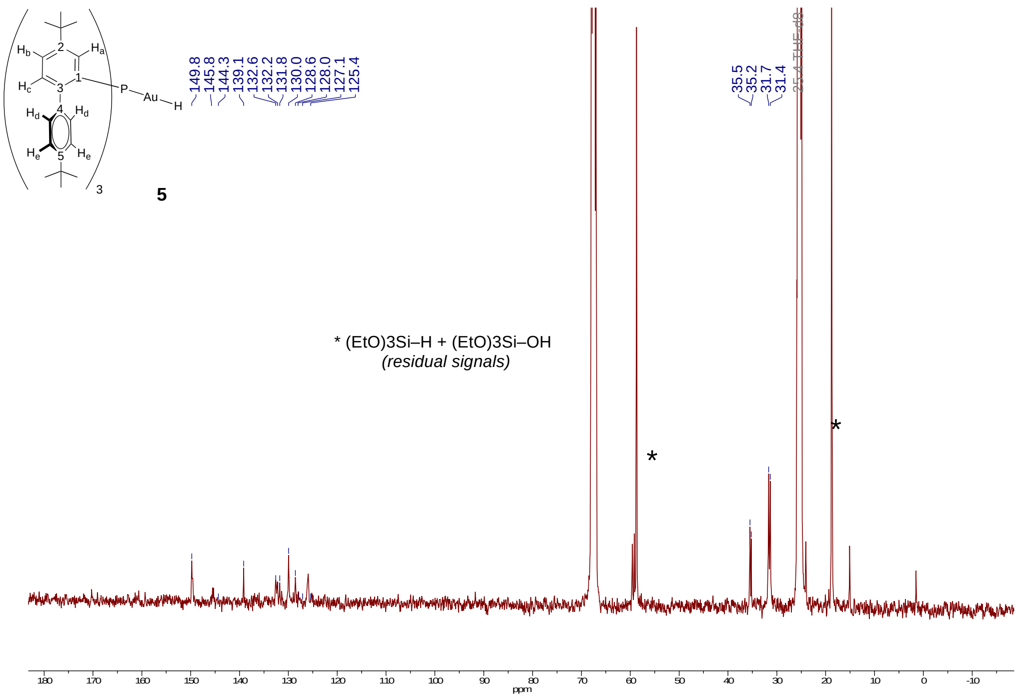

Figure S13. $^{13}C\{^1H\}$ NMR of complex **5** at –80 ºC.

$^{31}P\{^1H\}$ NMR (THF-d_6, 161 MHz, –80 ºC)

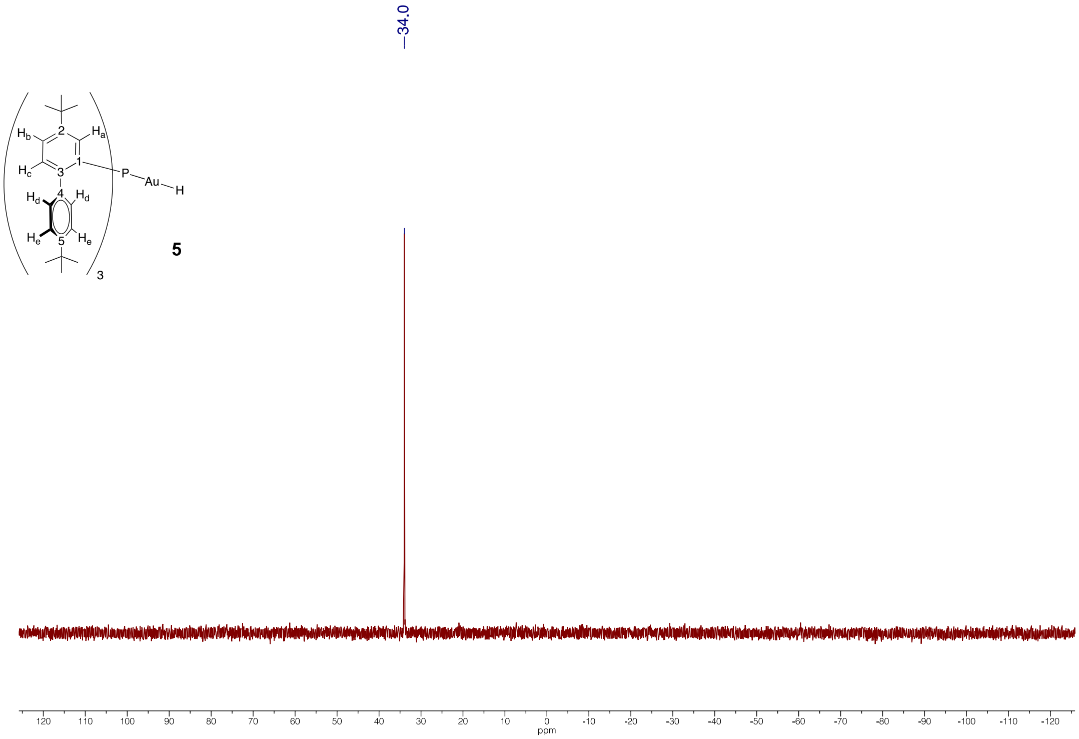

Figure S14. $^{31}P\{^1H\}$ NMR of complex **5** at –80 ºC.

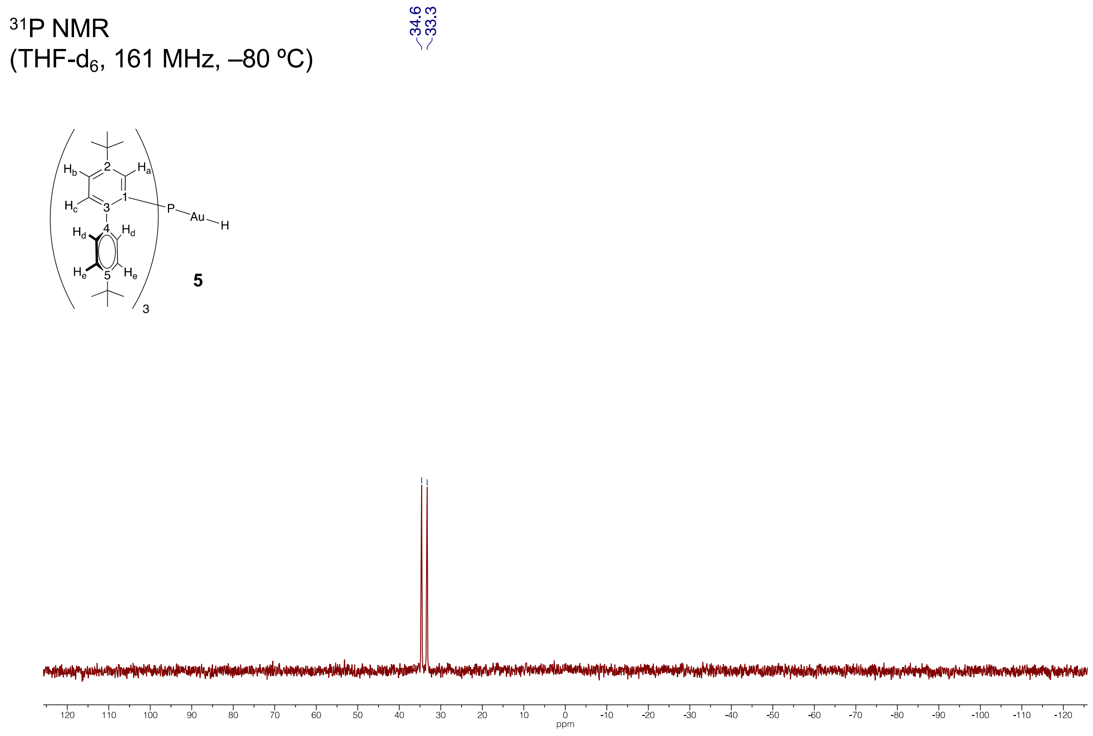

Figure S15. $^{31}P\{^{1}H\}$ NMR of complex **5** at –80 ºC.

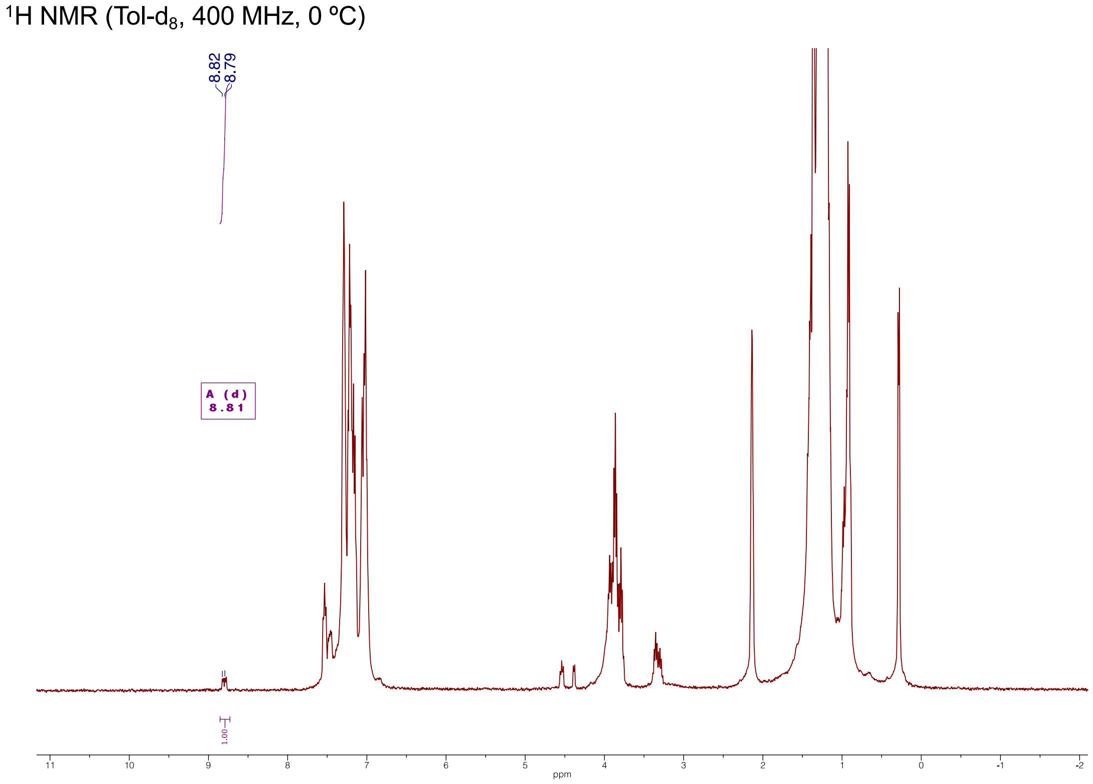

Figure S16. ^{1}H NMR of reaction of complex **5** with CO_2 at 0 ºC with formate proton signal.

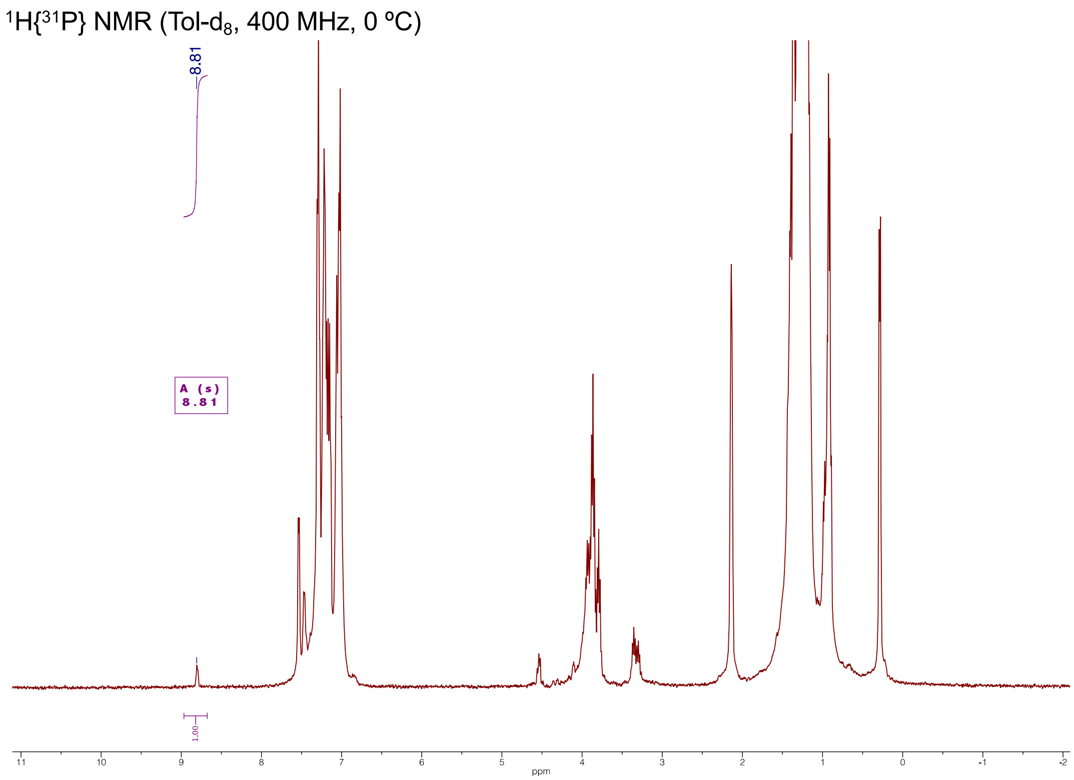

Figure S17. $^1H\{^{31}P\}$ NMR of reaction of complex **5** with CO_2 at 0 ºC with formate proton signal.

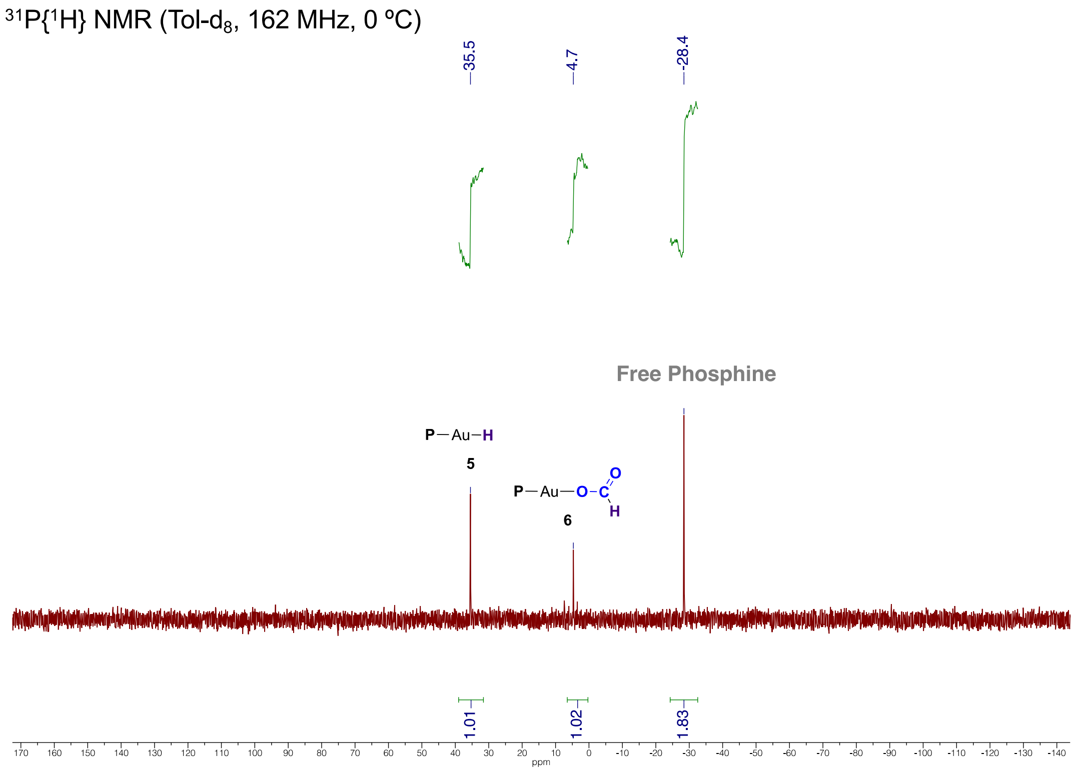

Figure S18. $^{31}P\{^1H\}$ NMR of c reaction of complex **5** with CO_2 at 0 ºC indicating a mixture of products.

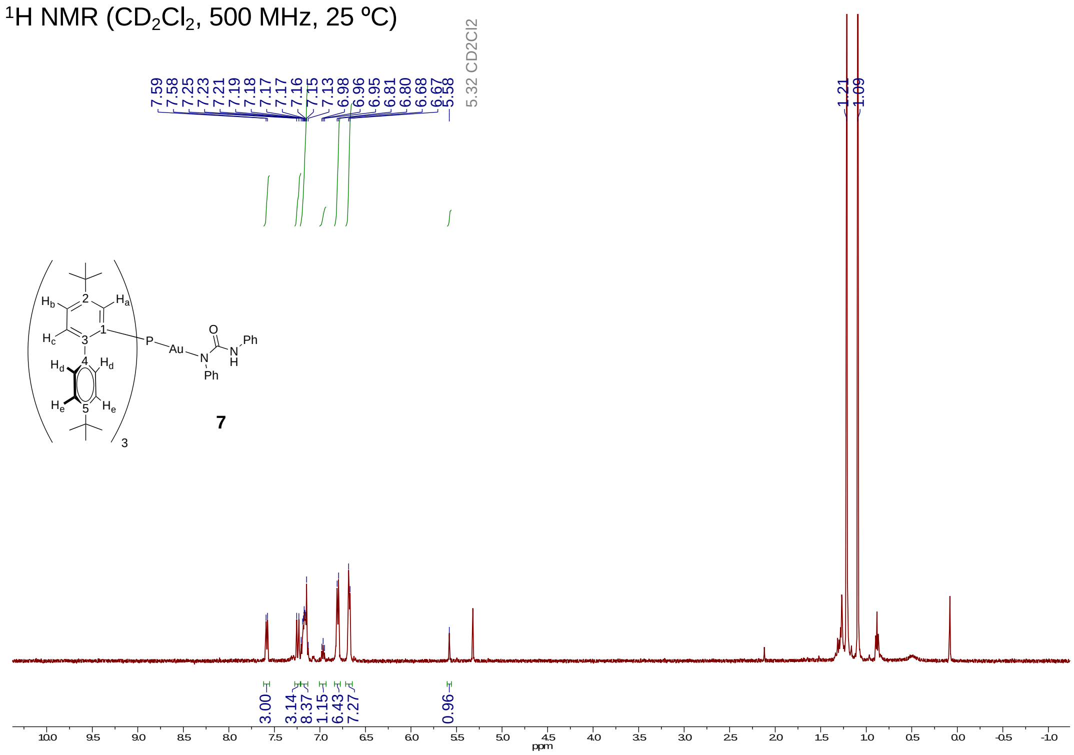

Figure S19. ^{1}H NMR of complex **7**.

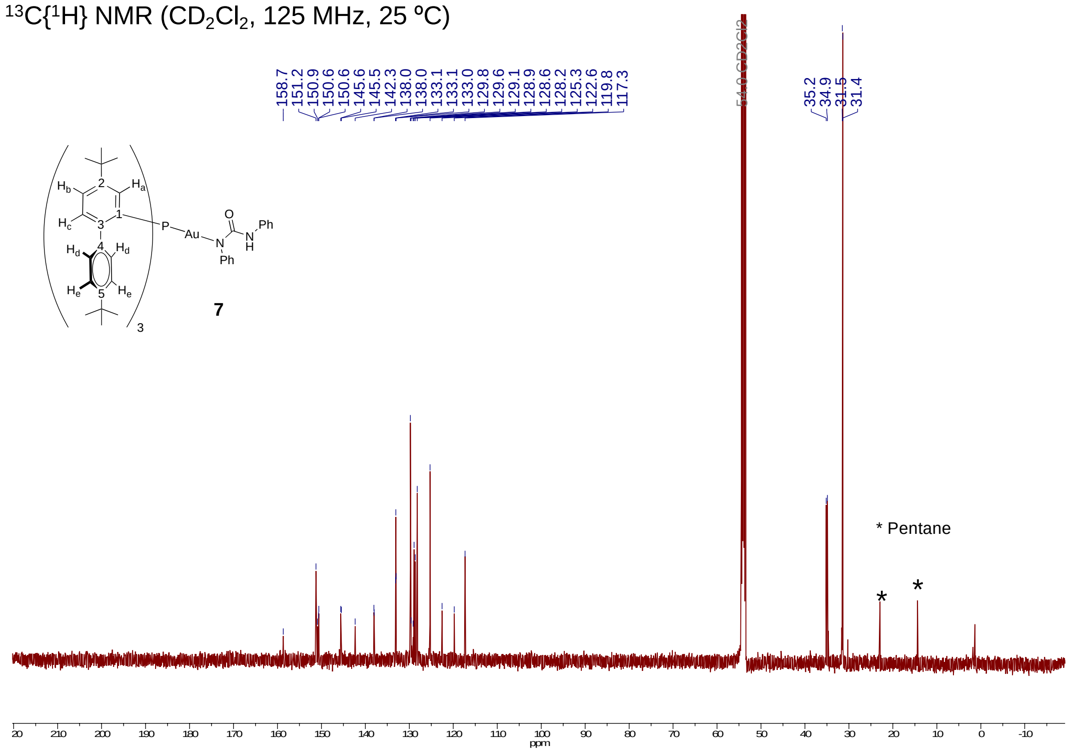

Figure S20. $^{13}C\{^{1}H\}$ NMR of complex **7**.

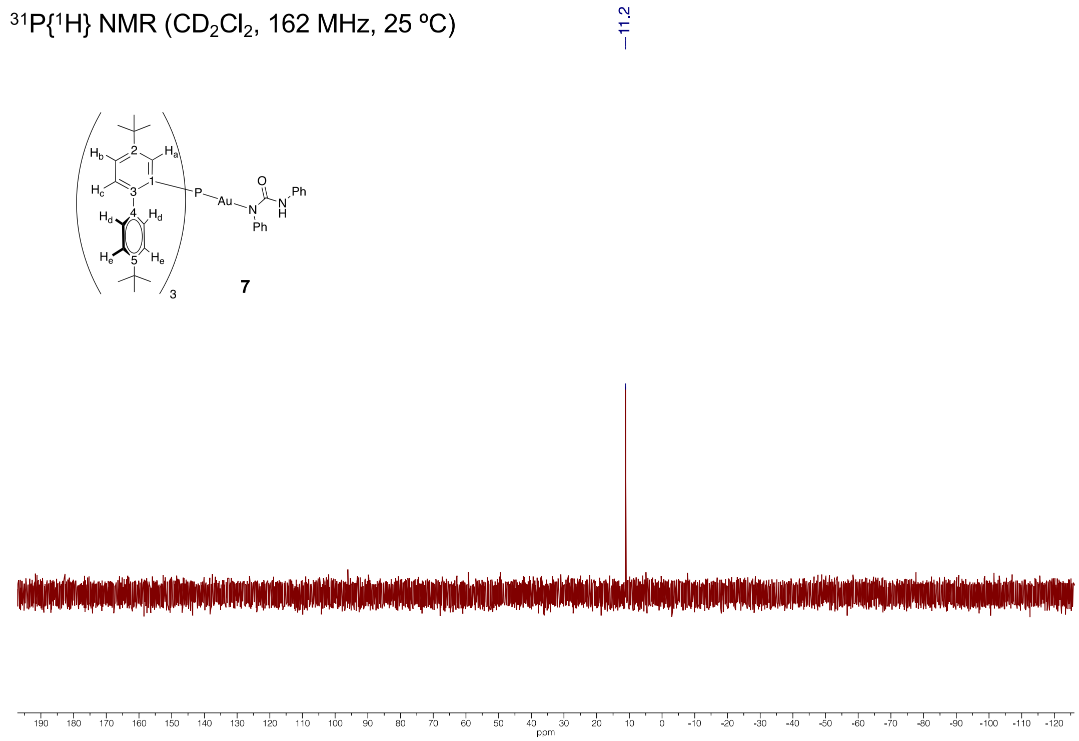

Figure S21. $^{31}P\{^1H\}$ NMR of complex **7**.

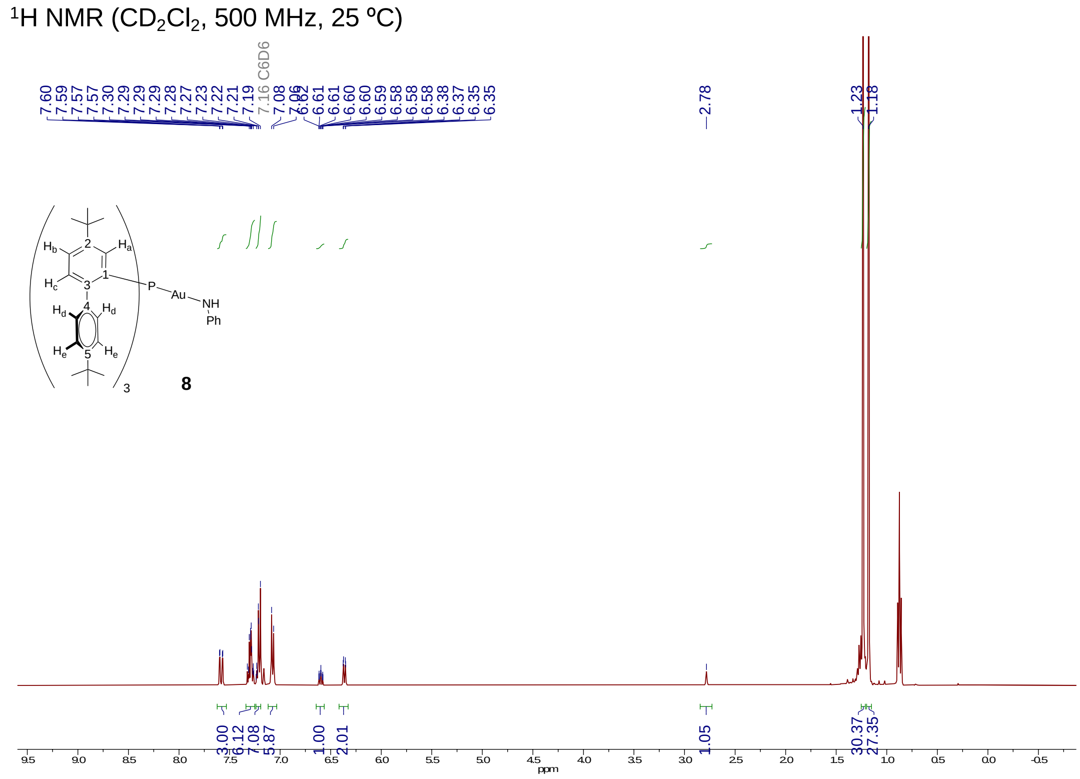

Figure S22. ^1H NMR of complex **8**.

$^{13}C\{^{1}H\}$ NMR (CD_2Cl_2, 125 MHz, 25 ºC)

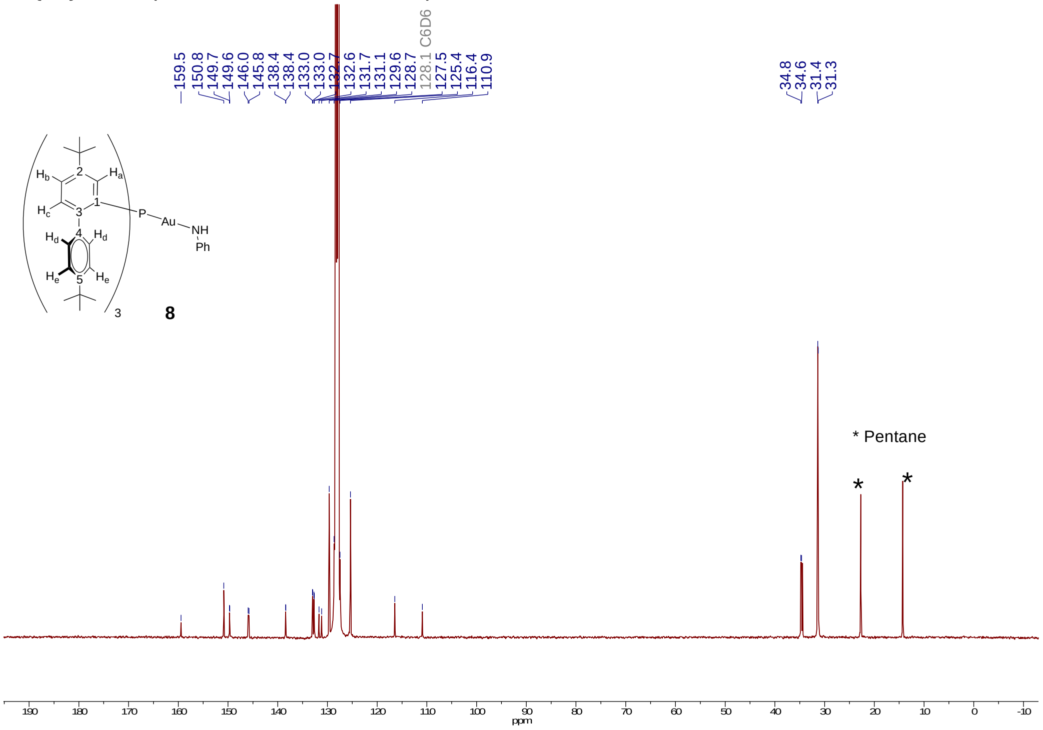

Figure S23. $^{13}C\{^{1}H\}$ NMR of complex **8**.

$^{31}P\{^{1}H\}$ NMR (CD_2Cl_2, 162 MHz, 25 ºC)

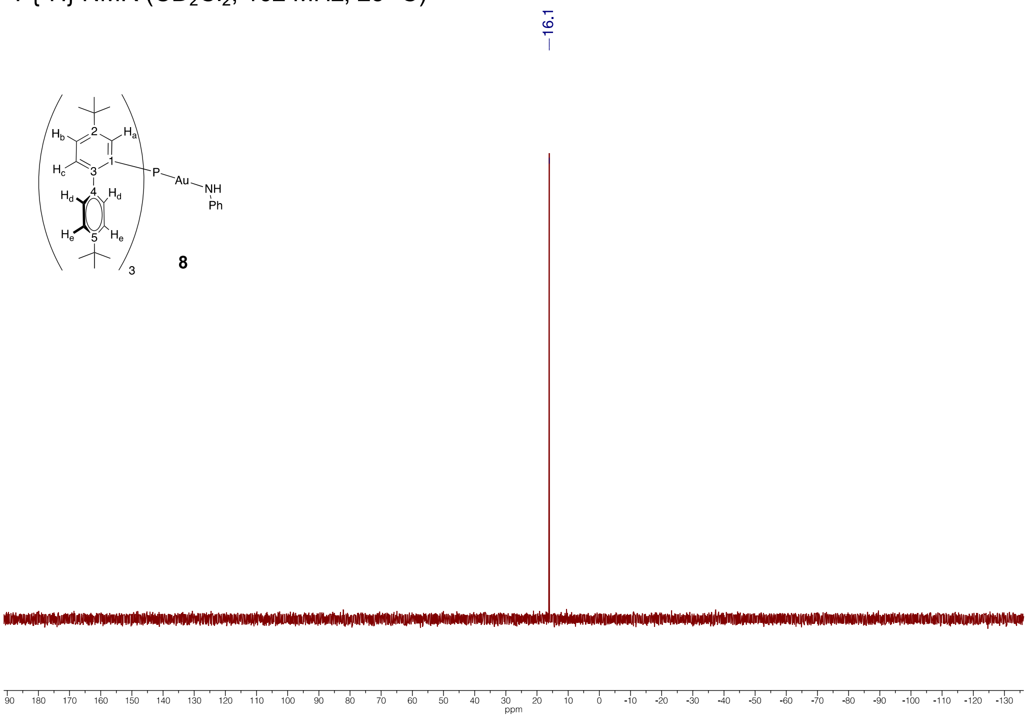

Figure S24. $^{31}P\{^{1}H\}$ NMR of complex **8**.

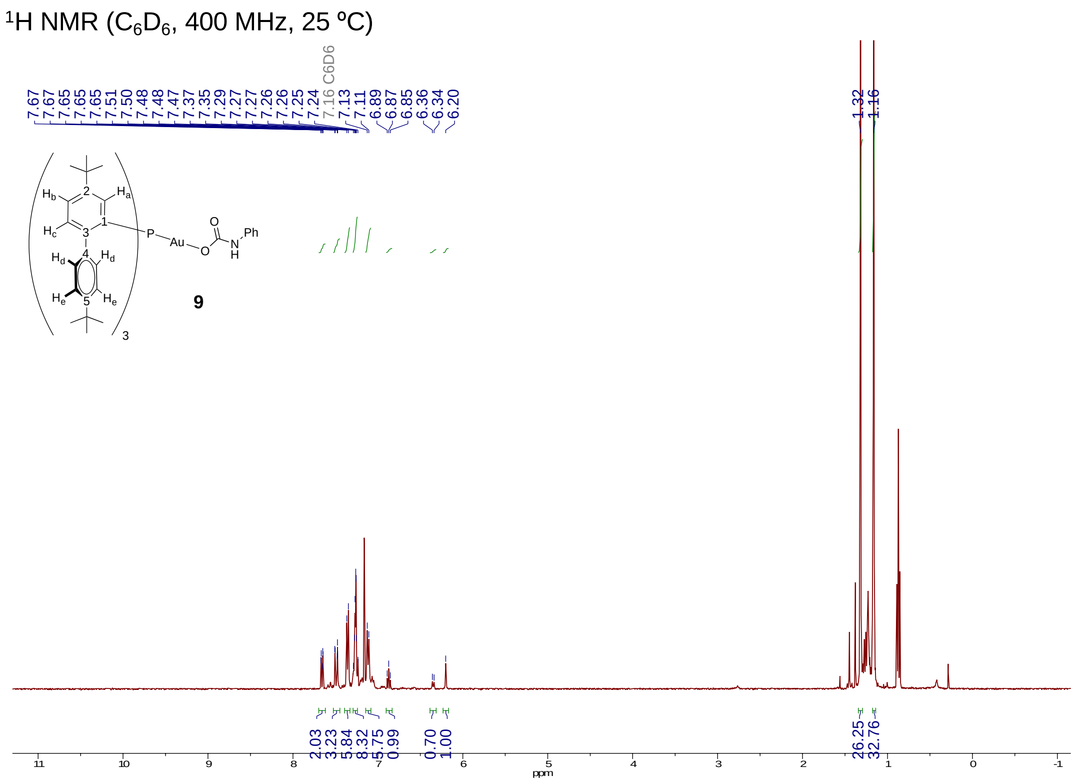

Figure S25. ^{1}H NMR of complex **9**.

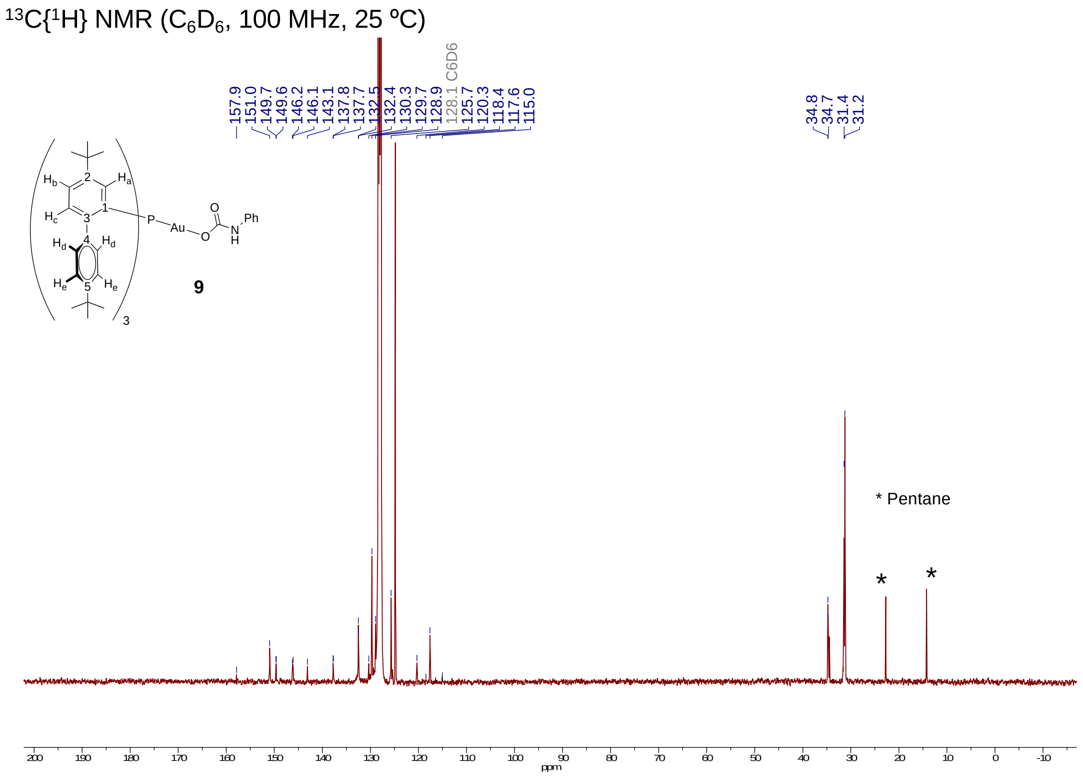

Figure S26. ^{13}C{^{1}H} NMR of complex **9**.

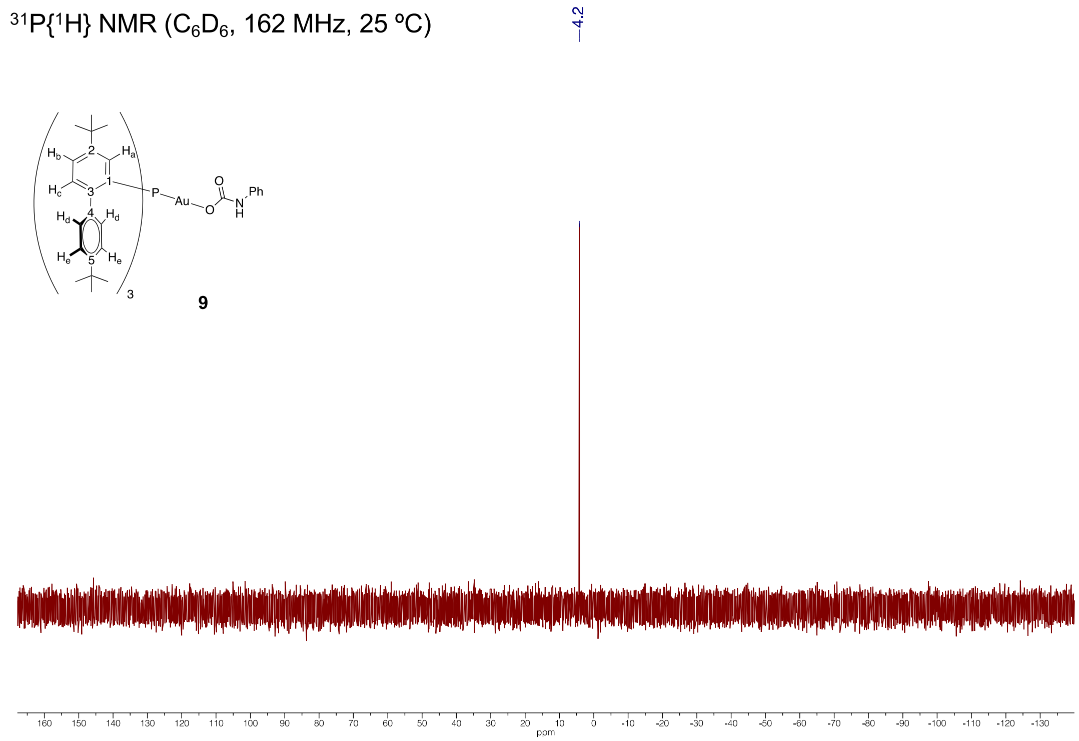

Figure S27. $^{31}P\{^{1}H\}$ NMR of complex **9**.

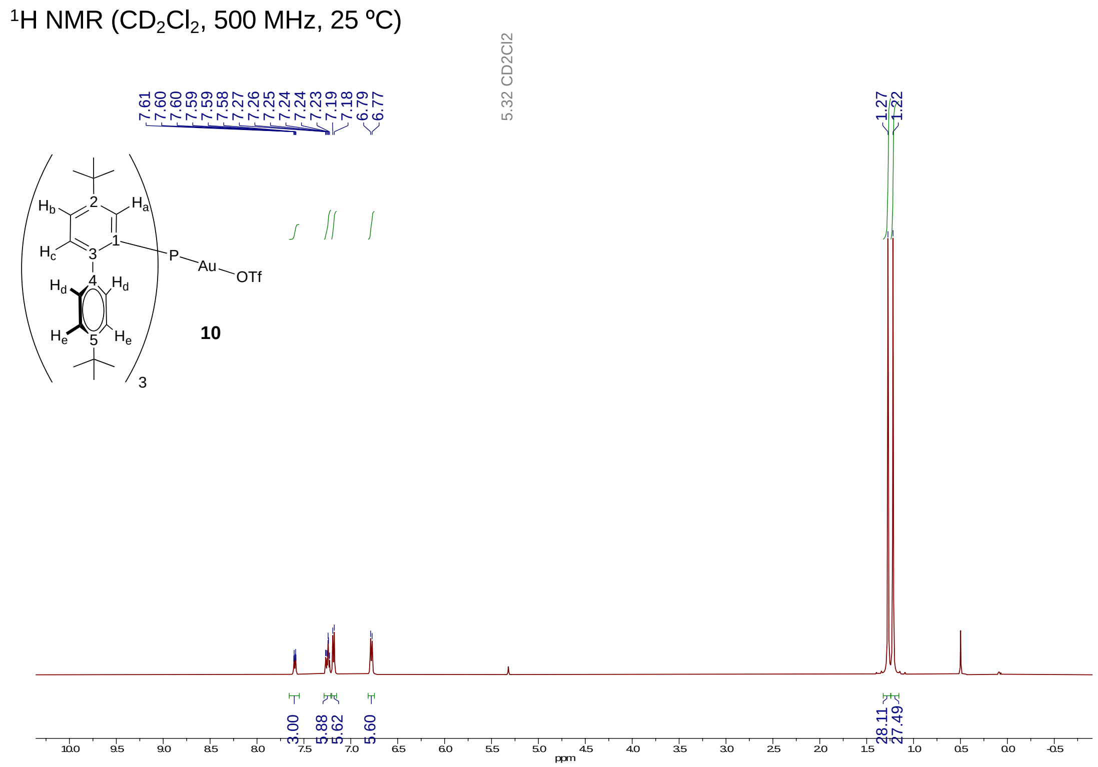

Figure S28. ^{1}H NMR of complex **10**.

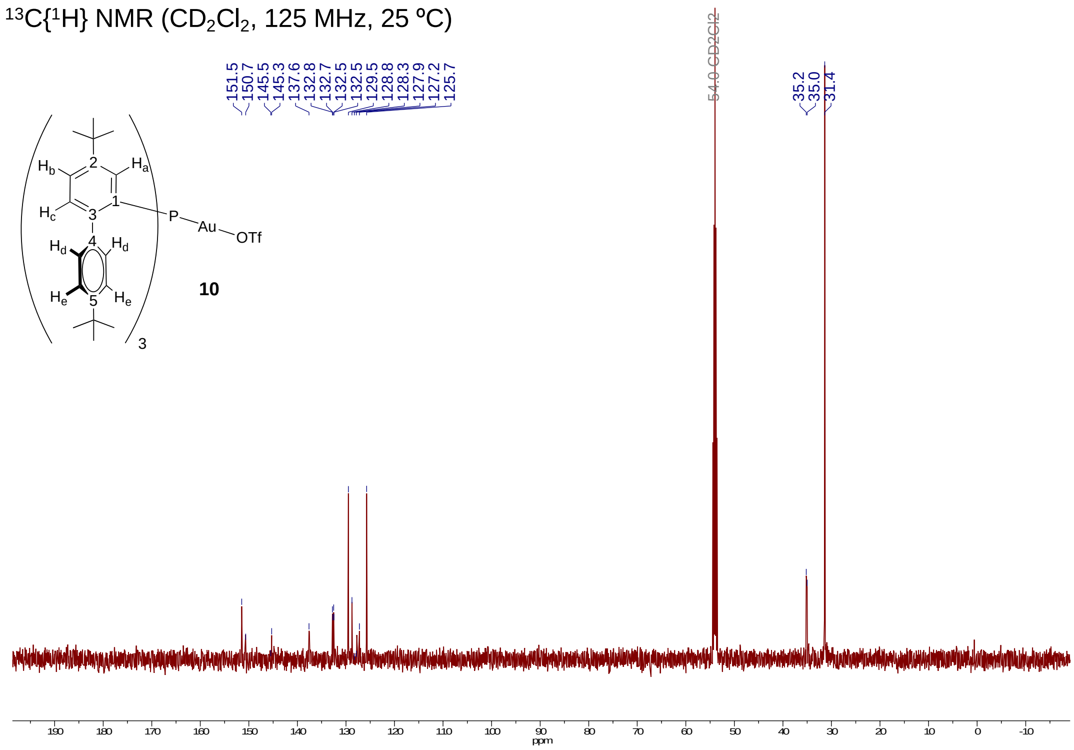

Figure S29. $^{13}C\{^{1}H\}$ NMR of complex **10**.

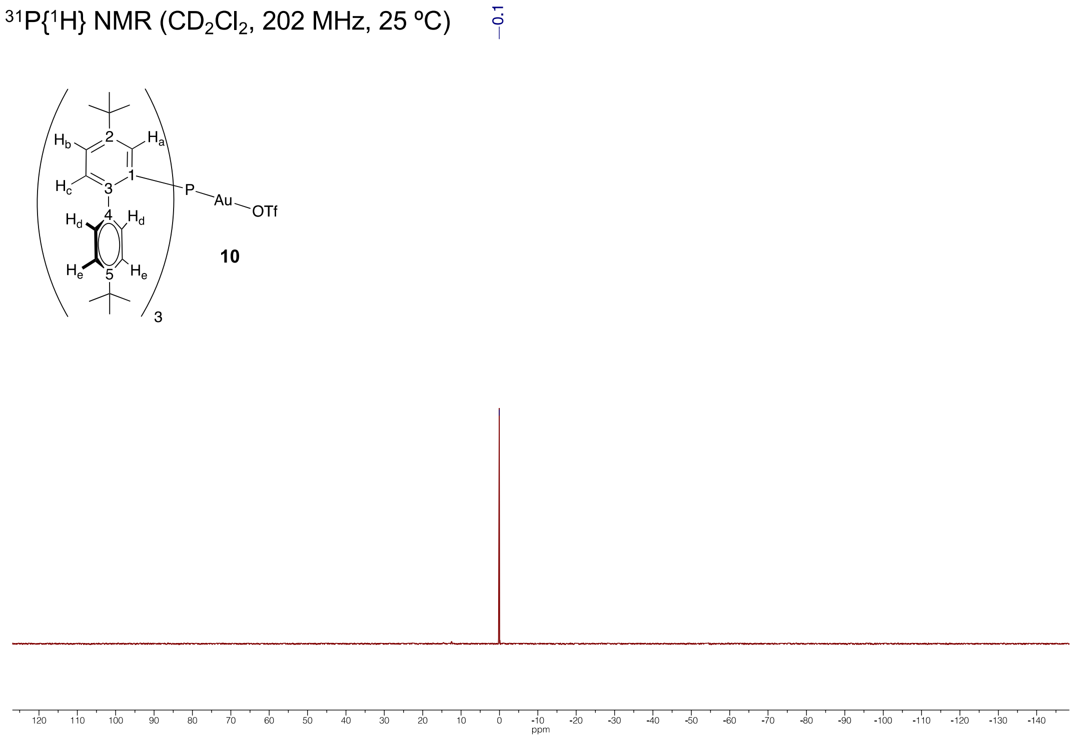

Figure S30. $^{31}P\{^{1}H\}$ NMR of complex **10**.

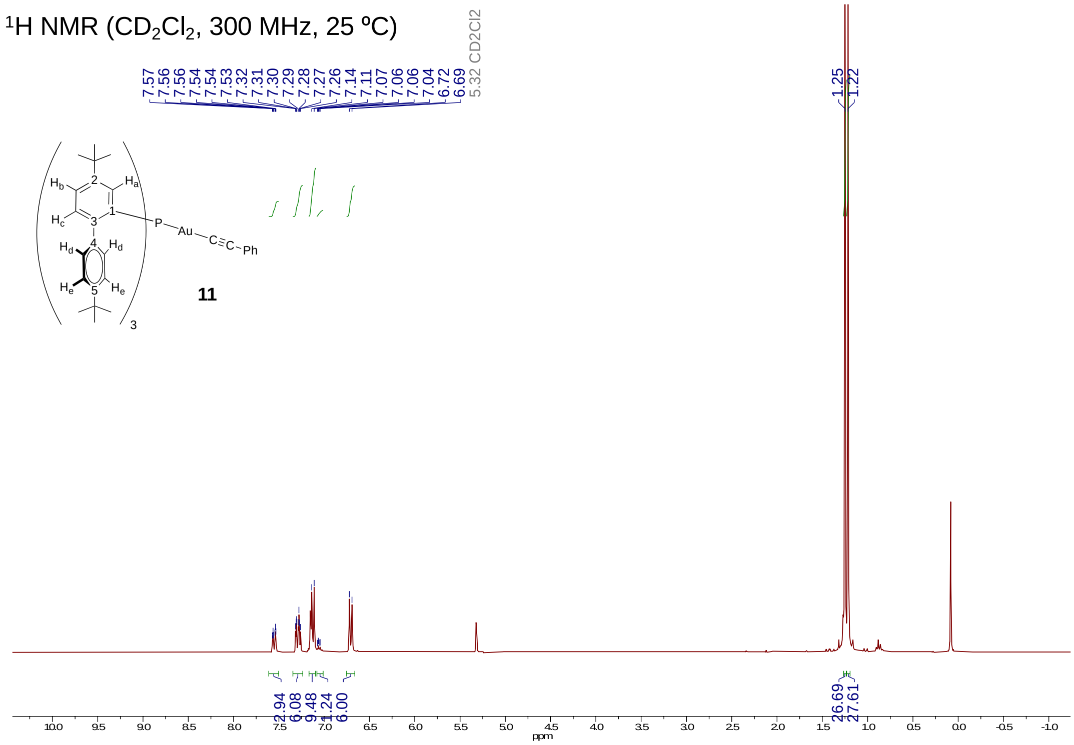

Figure S31. ^{1}H NMR of complex **11**.

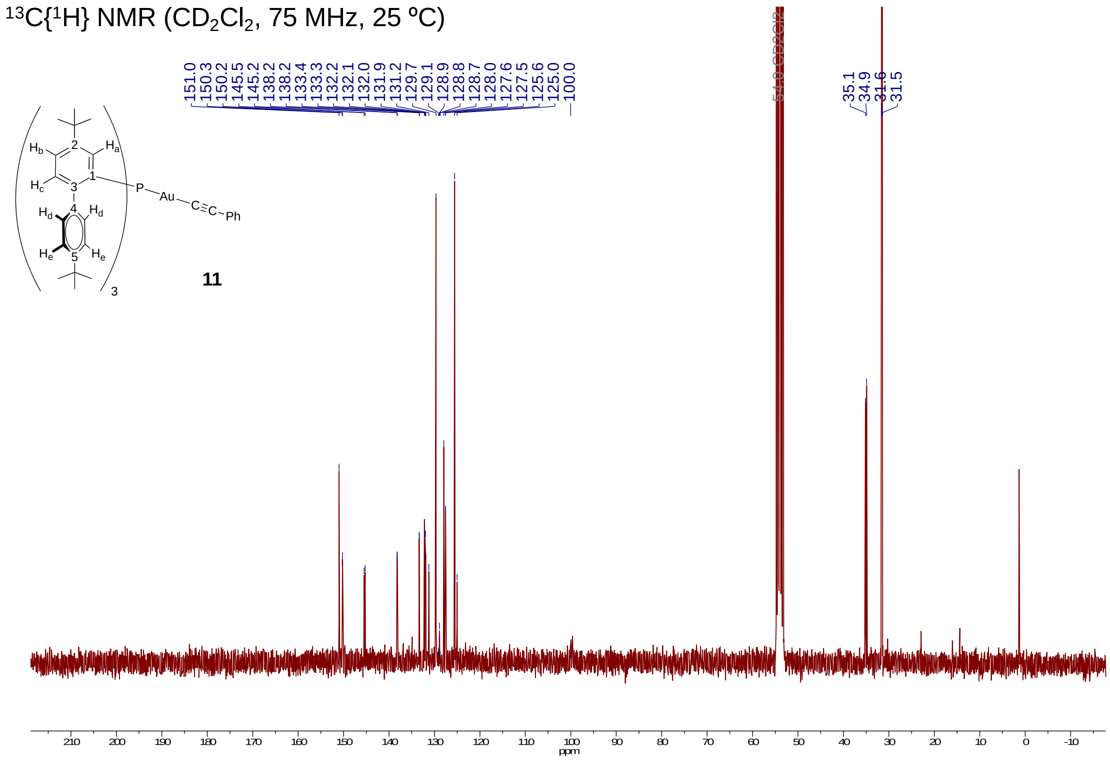

Figure S32. ^{13}C{^{1}H} NMR of complex **11**.

$^{31}P\{^1H\}$ NMR (CD_2Cl_2, 121 MHz, 25 ºC)

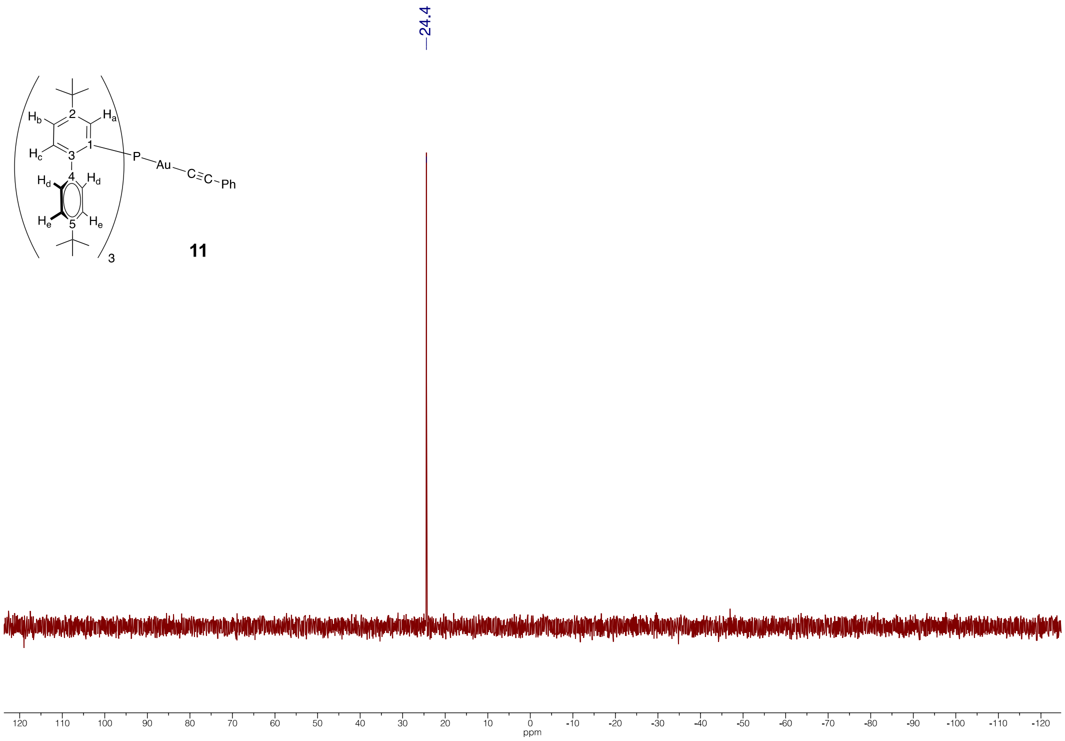

Figure S33. $^{31}P\{^1H\}$ NMR of complex **11**.

^1H NMR (CD_2Cl_2, 500 MHz, 25 ºC)

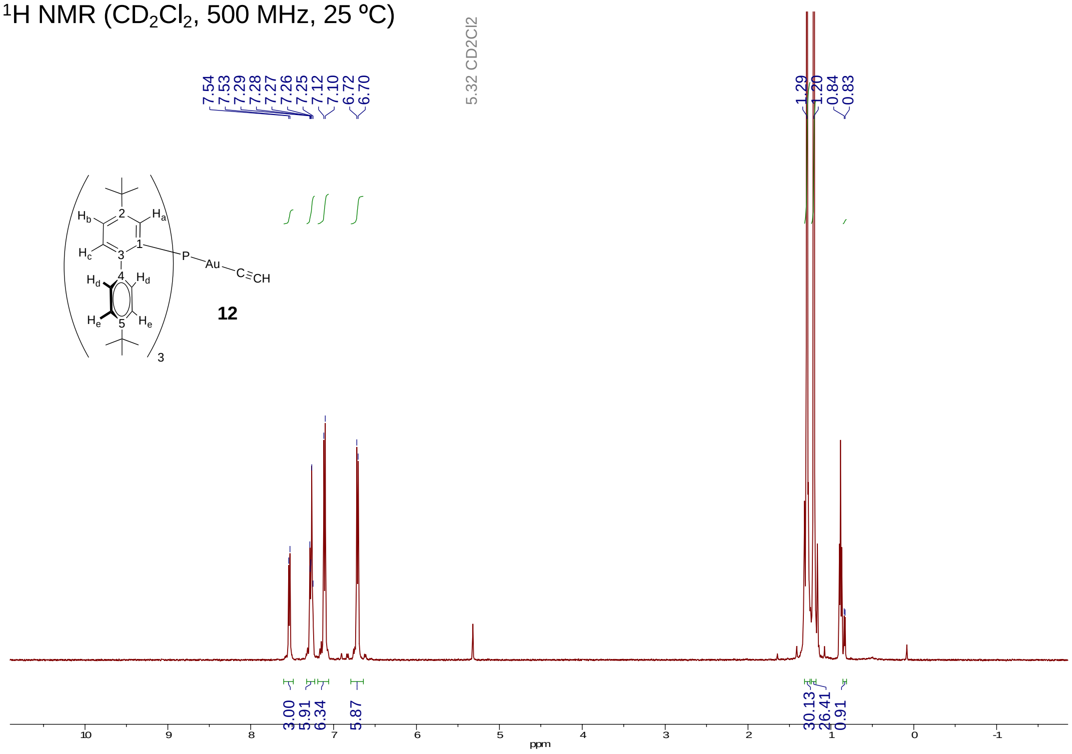

Figure S34. ^1H NMR of complex **12**.

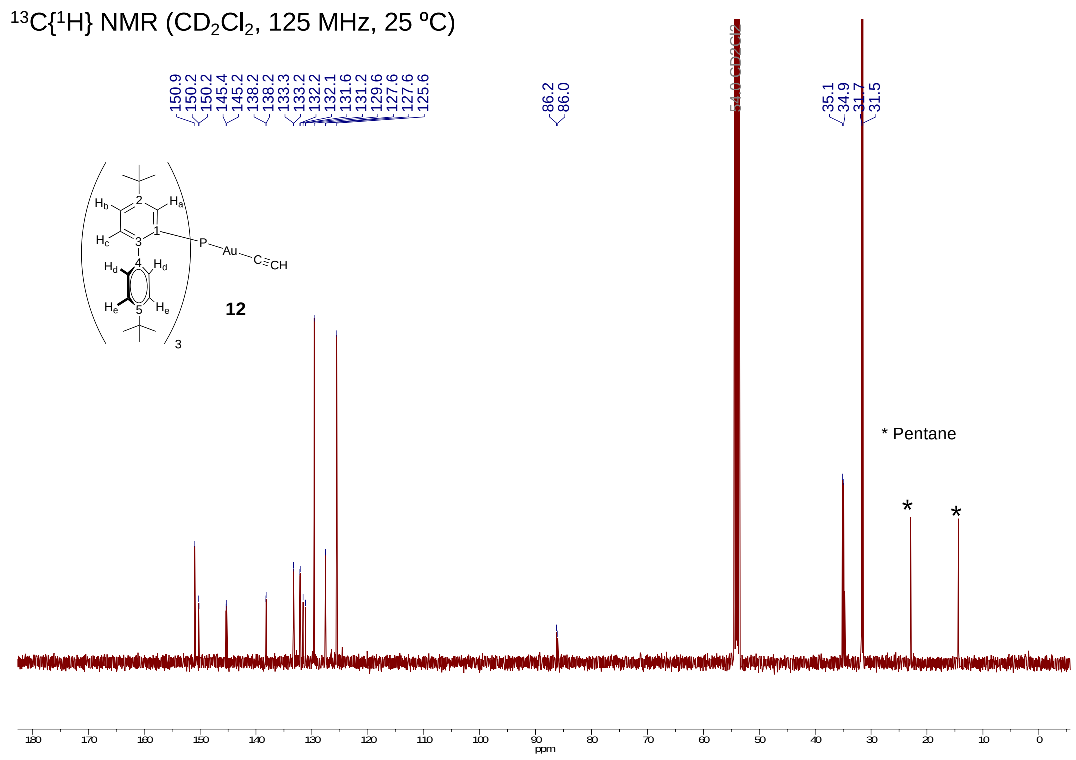

Figure S35. $^{13}C\{^{1}H\}$ NMR of complex **12**.

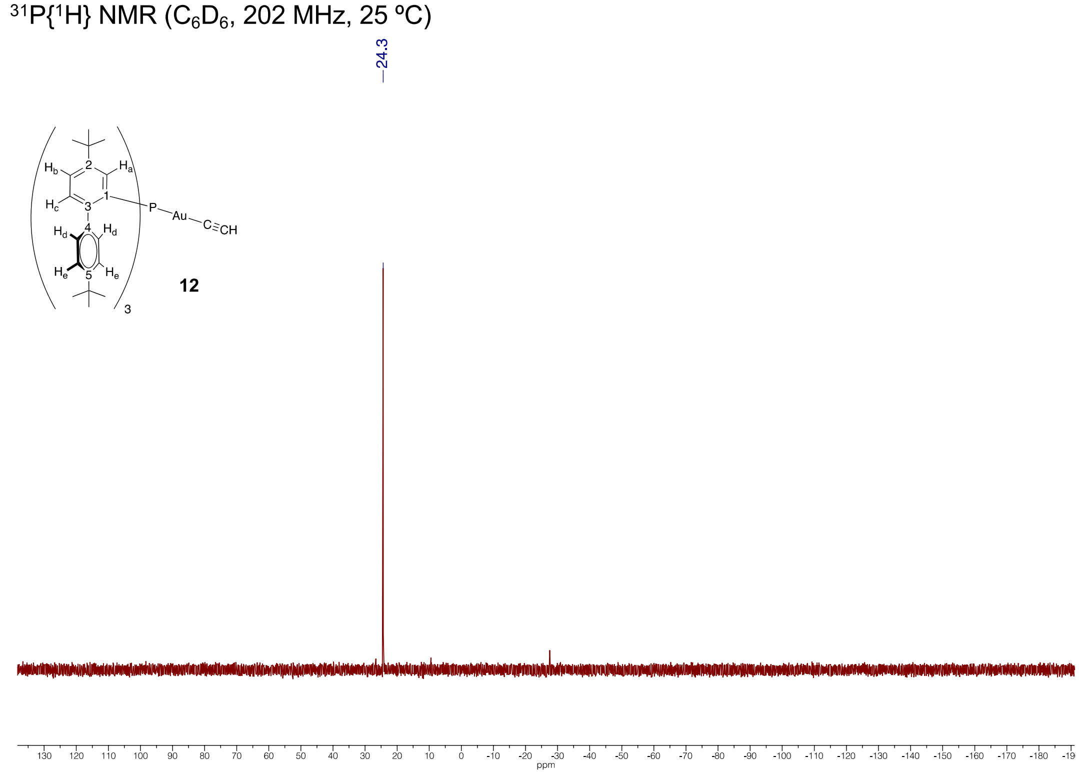

Figure S36. $^{31}P\{^{1}H\}$ NMR of complex **12**.

3. IR spectroscopic measurements

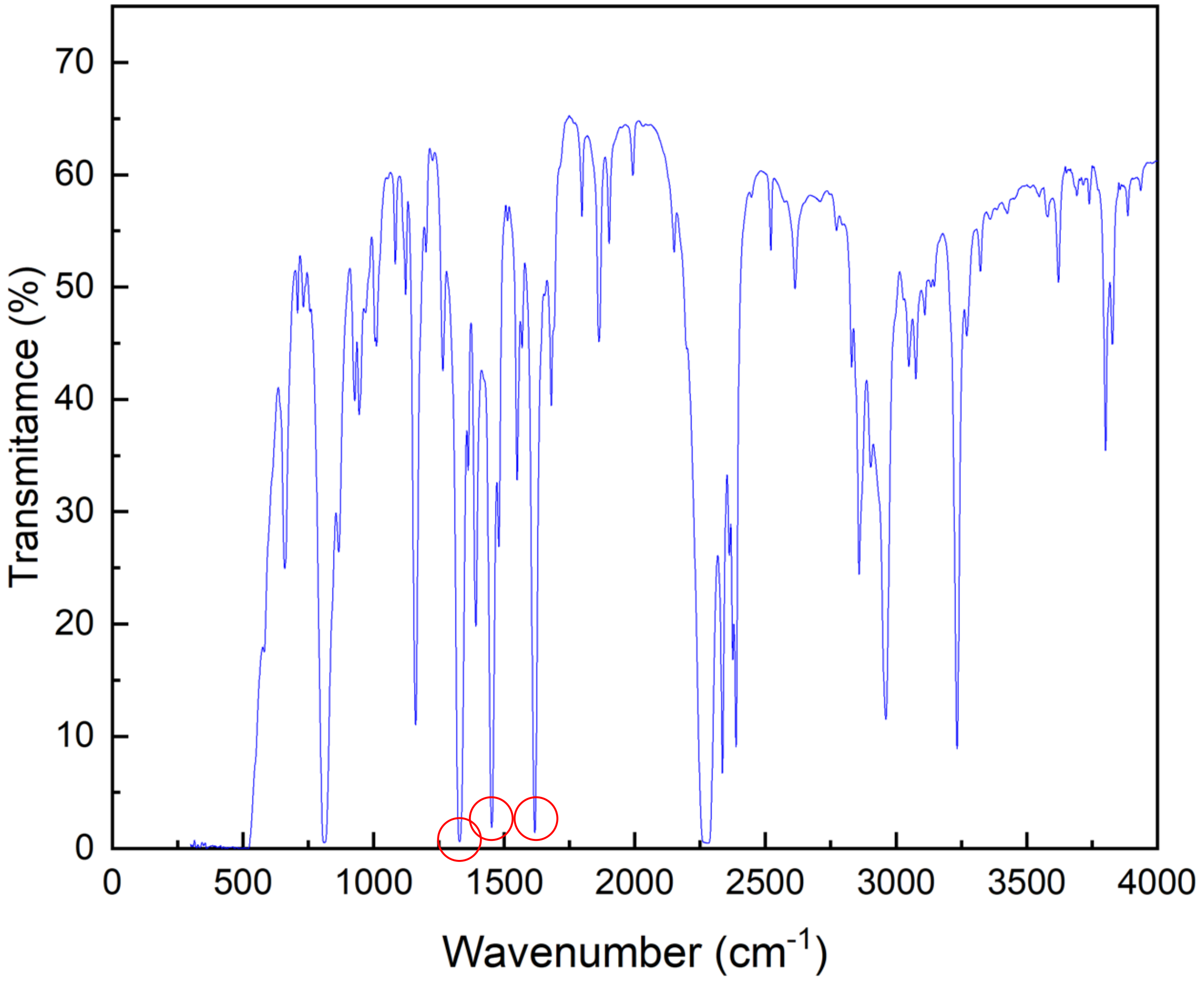

Figure S37. IR spectra of complex **3** (C–O stretching bands at 1618, 1453 and 1330 cm^{-1} marked with red cercles).

4. Crystal structure experiemnts

Crystallographic details. Low-temperature diffraction data were collected on a D8 Quest APEX-III single crystal diffractometer with a Photon III detector and a IμS 3.0 microfocus X-ray source at the Instituto de Investigaciones Químicas, Sevilla. Data were collected by means of ω and φ scans using monochromatic radiation λ(Mo Kα1) = 0.71073 Å. The diffraction images collected were processed and scaled using APEX-III software. All structures were solved using SHELXT and refined against F^2 on all data by full-matrix least squares with SHELXL.[1] All non-hydrogen atoms were refined anisotropically. Hydrogen atoms were included in the model at geometrically calculated positions and refined using a riding model. The isotropic displacement parameters of all hydrogen atoms were fixed to 1.2 times the U value of the atoms to which they are linked (1.5 times for methyl groups). In six of the seven reported structures we used the program SQUEEZE to compensate for the contribution of disordered solvent molecules and counteranions, which account for 1 pentane (**3**), 2 pentane (**4**), 4 pentane (**7**), 8 pentane **(8**), 2 pentane (**11**) and 3 dichloromethane (**12**) in the unit cell. Besides, compounds **3** and **10** contain as well 5 and 3 dichloromethane molecules, respectively, which could be modelled from the Fourier map. These, as well as some disordered tert-butyl groups were, modelled using anisotropic displacement parameter restraints. Compounds **3** and **7** contain two independent gold molecules per unit cell.

A summary of the fundamental crystal and refinement data are given in Table S1 and Table S2. Atomic coordinates, anisotropic displacement parameters and bond lengths and angles can be found in the cif files, which have been deposited in the Cambridge Crystallographic Data Centre with no. 2246839-2246845. These data can be obtained free of charge from The Cambridge Crystallographic Data Centre via www.ccdc.cam.ac.uk/data_request/cif.

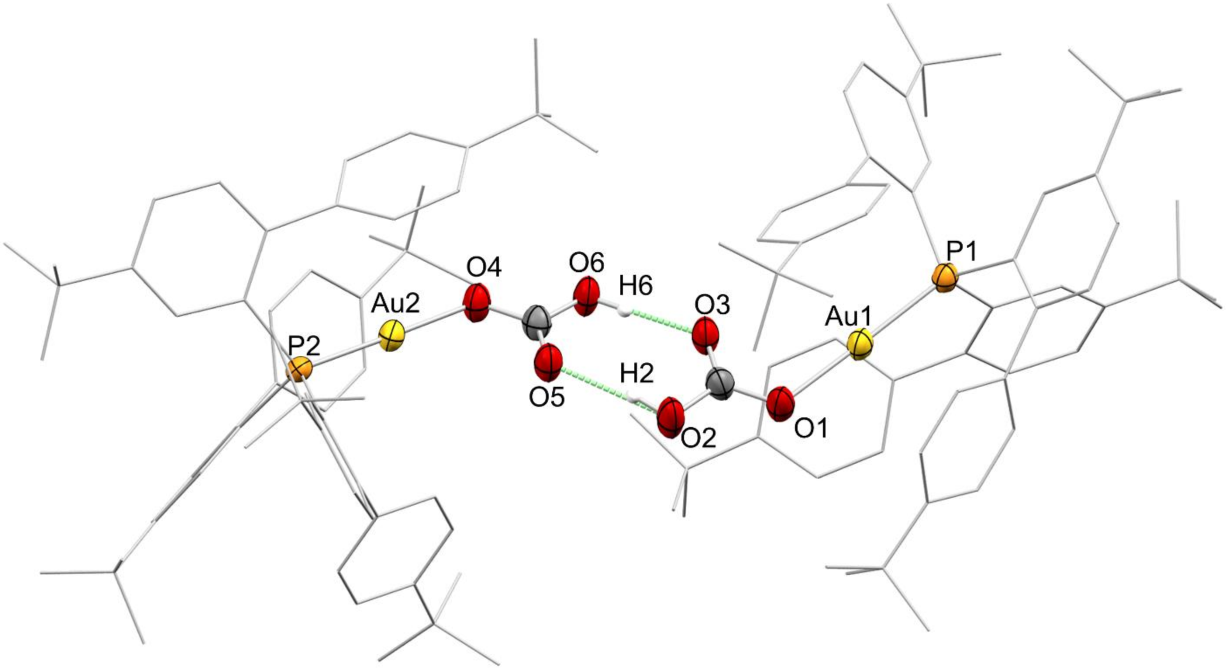

Figure S38. ORTEP diagram of complex **3** showing the hydrogen bonding interactions of the hydrogencarbonate moieties that connect the two independent molecules of gold. Hydrogen atoms, except those involve in the hydrogen bonding, are excluded for clarity. Thermal ellipsoids are set at 50% probability. All carbon atoms are represented in wireframe format for clarity. Hydrogen bonding is highlighted in green.

Table S1. Crystal data and structure refinement for compounds **3**, **4** and **7**.

	**3**	**4**	**7**
formula	$C_{127}H_{162}Au_2Cl_{10}O_6P_2$	$C_{60}H_{76}AuOP$	$C_{73}H_{86}AuN_2OP$
Fw	2594.93	1041.14	1235.37
cryst.size, mm	0.20 × 0.17 × 0.12	0.17 × 0.16 × 0.07	0.11 × 0.04 × 0.02
crystal system	Triclinic	Triclinic	Triclinic
space group	*P-1*	*P-1*	*P-1*
a, Å	14.1501 (8)	13.0604 (9)	11.2713 (17)
b, Å	20.1582 (11)	15.4921 (11)	21.610 (3)
c, Å	24.9884 (12)	16.6421 (12)	29.221 (4)
α, deg	83.825 (2)	105.091 (3)	95.032 (5)
β, deg	76.402 (2)	100.851 (3)	91.400 (5)
γ, deg	71.627 (2)	103.304 (3)	95.878 (5)
V, Å^3	6570.4 (6)	3051.5 (4)	7048.8 (18)
T, K	193	193	193
Z	2	2	4
ρ_{calc}, g cm^{-3}	1.312	1.133	1.164
μ, mm^{-1} (MoKα)	2.51	2.47	2.15
F(000)	2660	1076	2560
absorption corrections	multi-scan, 0.589–0.745	multi-scan, 0.594–0.745	multi-scan, 0.642–0.746
θ range, deg	1.9-26.4	2.2-26.5	1.9-28.3
no. of rflns measd	240018	54005	499391
R_{int}	0.106	0.101	0.114
no. of rflns unique	26880	12449	35032
no. of params / restraints	1361 / 159	586 / 114	1441 / 72
R_1 ($I > 2\sigma(I)$) [a]	0.0538	0.0652	0.0640
R_1 (all data)	0.0894	0.1007	0.0943
wR_2 ($I > 2\sigma(I)$)	0.1266	0.1580	0.1174
wR_2 (all data)	0.1461	0.1755	0.1300
Diff.Fourier.peaks min/max, eÅ^{-3}	-1.36 / 2.31	-1.03 / 1.56	-2.90/ 1.33
CCDC number	2246844	2246839	2246845

Table S2. Crystal data and structure refinement for compounds **8**, **10** and **11**.

	**8**	**10**	**11**
formula	$C_{66}H_{81}AuNP$	$C_{62}H_{77}AuCl_2F_3O_3PS$	$C_{68}H_{80}AuP$
Fw	1116.25	1258.13	1125.25
cryst.size, mm	0.17 × 0.14 × 0.06	0.20 × 0.08 × 0.03	0.12 × 0.08 × 0.07
crystal system	Monoclinic	Monoclinic	Triclinic
space group	$P2_1/n$	$P2_1/n$	*P-1*
a, Å	15.0688 (7)	14.917 (4)	13.2253 (14)
b, Å	27.3855 (15)	25.991 (7)	15.4964 (19)
c, Å	17.6724 (11)	16.112 (4)	16.7792 (18)
α, deg	90	90	105.404 (5)
β, deg	106.475 (2)	103.051 (11)	102.572 (5)
γ, deg	90	90	101.837 (6)
V, $Å^3$	6993.4 (7)	6085 (3)	3106.9 (6)
T, K	193	193	193
Z	4	4	2
ρ_{calc}, g cm^{-3}	1.060	1.373	1.203
μ, mm^{-1} (MoKα)	2.16	2.62	2.43
F(000)	2312	2576	1164
absorption corrections	multi-scan, 0.589–0.745	multi-scan, 0.589–0.745	multi-scan, 0.589–0.745
θ range, deg	2.5–27.0	2.3-28.2	2.3-27.1
no. of rflns measd	207756	109272	106519
R_{int}	0.076	0.101	0.052
no. of rflns unique	15433	11769	12191
no. of params / restraints	640 / 0	676 / 48	649 / 113
R_1 ($I > 2\sigma(I)$) [a]	0.0289	0.0580	0.0639
R_1 (all data)	0.0481	0.0714	0.0706
wR_2 ($I > 2\sigma(I)$)	0.0676	0.1710	0.1553
wR_2 (all data)	0.0802	0.1839	0.1600
Diff.Fourier.peaks min/max, e$Å^{-3}$	-0.89 / 1.61	-1.33 / 2.17	-2.81 / 4.15
CCDC number	2246843	2246841	2246842

Table S3. Crystal data and structure refinement for compounds **12**.

	**12**
formula	$C_{63}H_{78}AuCl_2P$
Fw	1134.09
cryst.size, mm	0.21 × 0.18 × 0.12
crystal system	Trigonal
space group	*R-3*
a, Å	48.2649 (11)
b, Å	48.2649 (11)
c, Å	13.4071 (4)
α, deg	90
β, deg	90
γ, deg	120
V, Å^3	27047.6 (15)
T, K	193
Z	18
ρ_{calc}, g cm^{-3}	1.253
μ, mm^{-1} (MoKα)	2.60
F(000)	10512
absorption corrections	multi-scan, 0.552–0.746
θ range, deg	1.8-28.3
no. of rflns measd	108900
R_{int}	0.065
no. of rflns unique	14925
no. of params / restraints	607 / 4
R_1 ($I > 2\sigma(I)$) [a]	0.0466
R_1 (all data)	0.0776
wR_2 ($I > 2\sigma(I)$)	0.1210
wR_2 (all data)	0.1430
Diff.Fourier.peaks min/max, eÅ^{-3}	-1.08 / 1.51
CCDC number	2246840

4. Computational details

Calculations were performed at the DFT level with the Gaussian 09 (Revision D.01) program.[2] The hybrid functional PBE0[3] was used throughout the computational study, and dispersion effects were accounted for by using Grimme's D3 parameter set with Becke–Johnson (BJ) damping at the optimization stage.[4] Geometry optimizations were carried out without geometry constraints, using the 6-31G(d,p)[5] basis set to represent the C, H, O and P atoms and the Stuttgart/Dresden Effective Core Potential and its associated basis set (SDD)[6] to describe the Au atoms. Bulk solvent effects (dichloromethane) were included at the optimization stage with the SMD continuum model[7]. The stationary points and their nature as minima or saddle points (TS) were characterized by vibrational analysis, which also produced enthalpy (H), entropy (S) and Gibbs energy (G) data at 298.15 K. The minima connected by a given transition state were determined by perturbing the transition states along the TS coordinate and optimizing to the nearest minimum.

4.1 Carbon dioxide insertion reactions

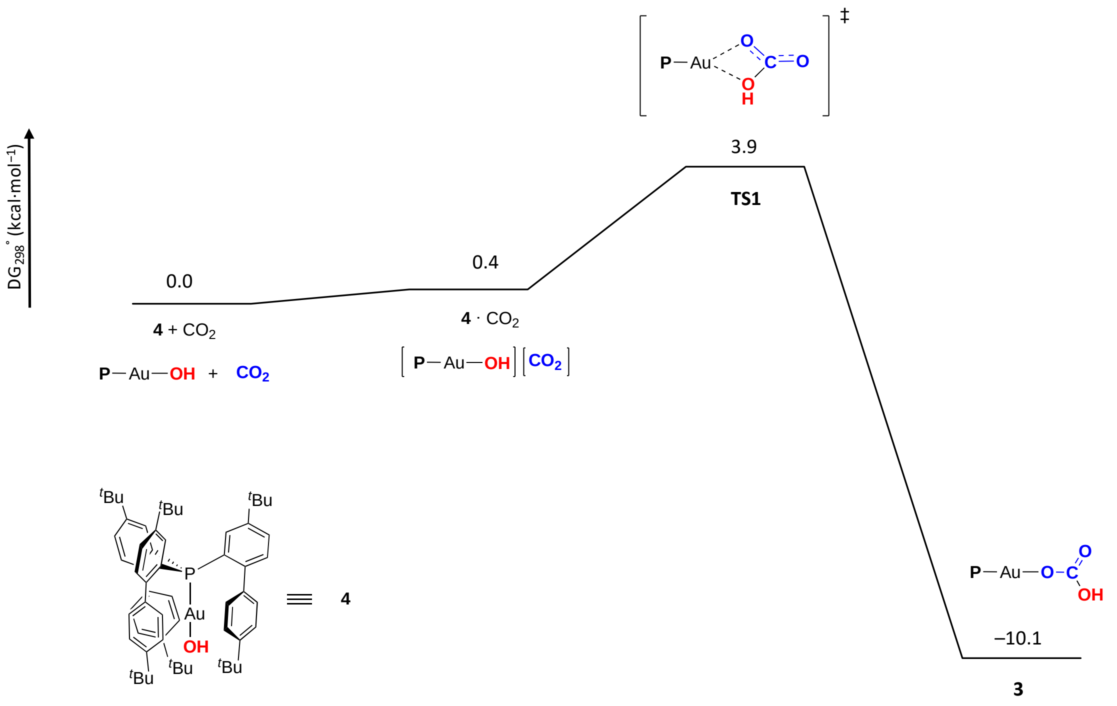

Figure S39. Free energy profile for the CO_2 insertion into the Au–OH bond of complex **4**.

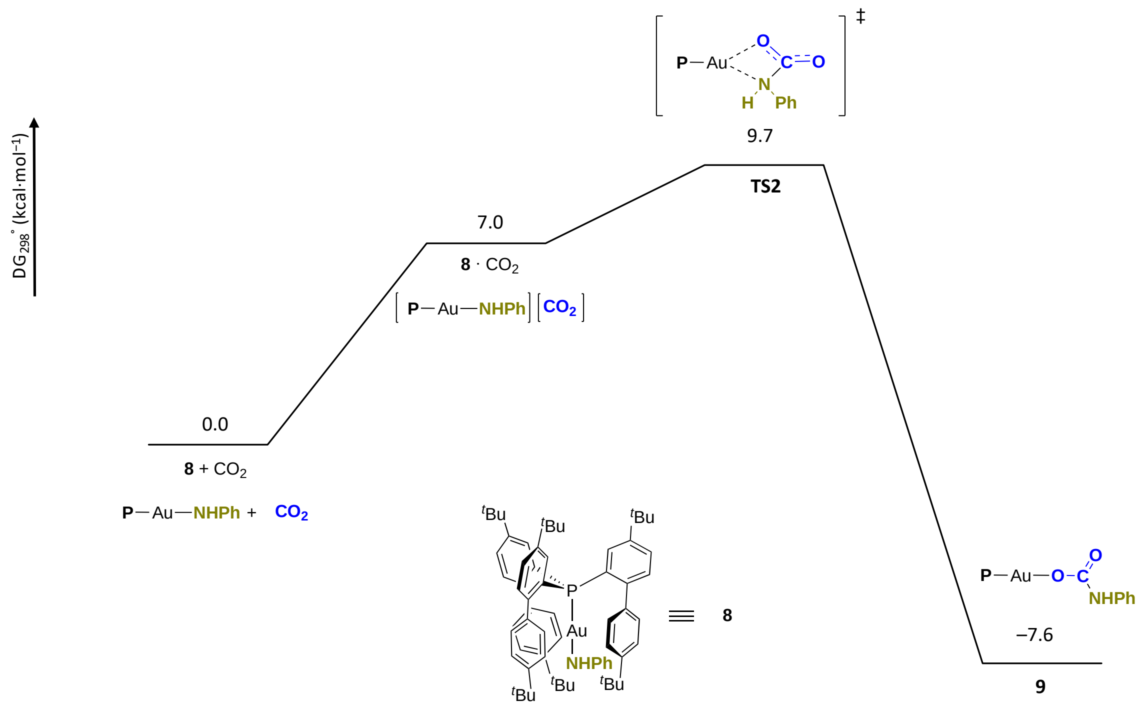

Figure S40. Free energy profile for the CO_2 insertion into the Au–NH bond of complex **8**.

4.2 Au(I)-Hydride Formation

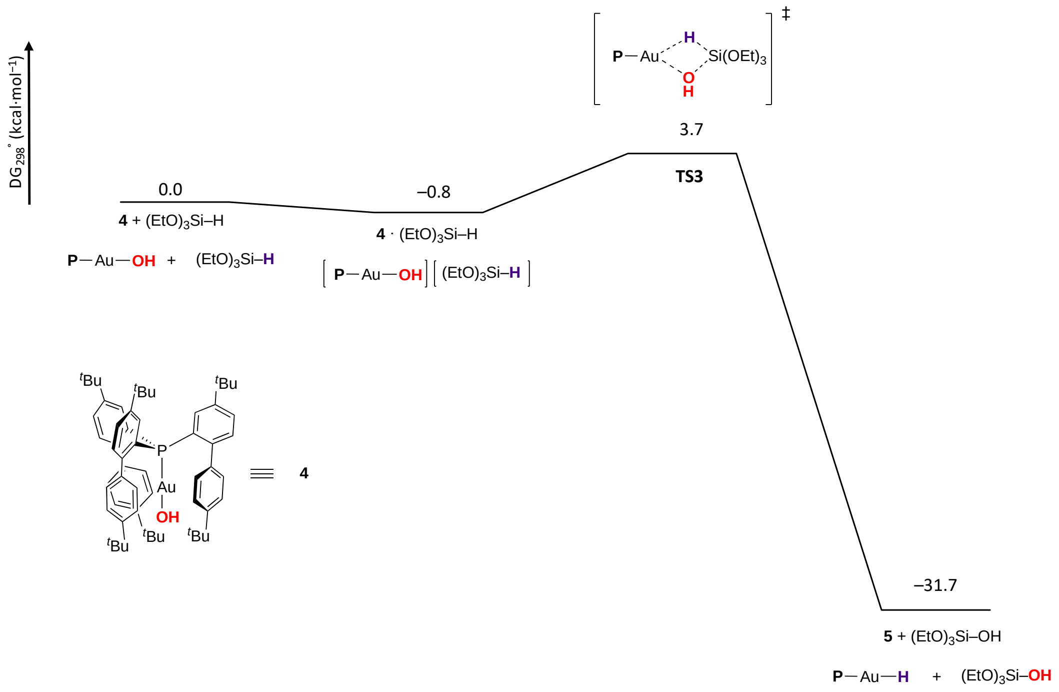

Figure S41. Free energy profile for the formation of complex **5**.

4.3 Cartesian coordinates and energies for all the species discussed in the text

CO2 E(RPBE1PBE) = -188.380075454

C	0.000000000	0.000000000	0.000000000
O	0.000000000	0.000000000	1.165547000
O	0.000000000	0.000000000	-1.165547000

(EtO)3SiH E(RPBE1PBE) = -752.917760209

Si	0.198328000	-0.238458000	0.166545000
H	0.054121000	0.002835000	1.627190000
O	1.767867000	-0.571300000	-0.196637000
O	-0.595322000	-1.568986000	-0.375830000
O	-0.350327000	1.105969000	-0.612984000
C	-0.805265000	2.277090000	0.055964000
H	-0.405510000	2.319683000	1.078857000
H	-0.399429000	3.138786000	-0.486019000
C	-2.316289000	2.331947000	0.083880000
H	-2.726300000	1.495530000	0.660216000
H	-2.654669000	3.264980000	0.547017000
H	-2.722270000	2.284124000	-0.931503000
C	2.789373000	0.354625000	0.165687000
H	2.769856000	1.207636000	-0.525575000
H	2.611855000	0.748203000	1.177917000
C	4.128778000	-0.340888000	0.109643000
H	4.316658000	-0.732038000	-0.895116000
H	4.931040000	0.359359000	0.363574000
H	4.160459000	-1.175572000	0.816899000
C	-2.002091000	-1.591970000	-0.596786000
H	-2.320144000	-0.663807000	-1.088642000
H	-2.195217000	-2.414519000	-1.293929000

C	-2.771403000	-1.804412000	0.689756000
H	-2.450252000	-2.727512000	1.182442000
H	-2.619122000	-0.973949000	1.387962000
H	-3.844038000	-1.879143000	0.481817000

(EtO)3SiOH E(RPBE1PBE) = -828.130678125

Si	-0.055076000	-0.020357000	0.682385000
O	1.419524000	-0.396110000	0.065272000
O	-1.163974000	-0.758390000	-0.276211000
O	-0.288992000	-0.546910000	2.222048000
H	0.178719000	-0.051257000	2.903970000
O	-0.206126000	1.612974000	0.695166000
C	1.778937000	-1.734147000	-0.275520000
H	1.058598000	-2.136437000	-0.998823000
H	1.740648000	-2.368266000	0.620521000
C	3.171855000	-1.732293000	-0.858435000
H	3.471238000	-2.750238000	-1.127878000
H	3.211811000	-1.110695000	-1.758682000
H	3.893400000	-1.339704000	-0.135020000
C	-0.114869000	2.373350000	-0.510082000
H	-0.803846000	3.218558000	-0.402566000
H	-0.464346000	1.777554000	-1.364403000
C	1.296375000	2.863566000	-0.746147000
H	1.640494000	3.464202000	0.101908000
H	1.979495000	2.019021000	-0.874089000
H	1.335575000	3.485478000	-1.646901000
C	-2.562636000	-0.561659000	-0.079178000
H	-2.771844000	0.503311000	0.090464000
H	-2.886044000	-1.108745000	0.815699000
C	-3.304929000	-1.054209000	-1.298336000

H	-3.104349000	-2.116730000	-1.468544000
H	-4.383527000	-0.923769000	-1.164036000
H	-2.996811000	-0.497433000	-2.189024000

4 E(RPBE1PBE) = -2881.92811412

Au	-1.512825000	-0.010625000	-0.050087000
C	3.390418000	-3.184285000	-0.094923000
C	3.268206000	1.542162000	2.817785000
C	0.968062000	-2.550959000	1.259797000
C	-0.270600000	-2.261490000	2.016854000
C	-2.688686000	-1.651123000	3.365195000
C	-0.202962000	-0.748387000	-2.990061000
C	0.992283000	0.116107000	-2.864983000
C	3.300294000	1.775359000	-2.713640000
C	-3.993039000	-1.239976000	4.046367000
C	-2.568108000	-2.310582000	-3.049887000
C	4.704693000	-3.531754000	-0.789532000
C	4.546036000	2.646002000	-2.561030000
C	-4.034175000	4.242393000	-1.049021000
C	-2.727565000	3.780286000	-0.405318000
C	0.875490000	2.364379000	1.510222000
C	-0.350124000	2.869207000	0.848498000
C	5.758311000	-3.873569000	0.274183000
H	6.711088000	-4.124540000	-0.205301000
H	5.928041000	-3.023639000	0.944645000
H	5.456720000	-4.729800000	0.885527000
C	5.233156000	-2.372676000	-1.636809000
H	4.538250000	-2.099133000	-2.438224000
H	5.424049000	-1.480963000	-1.029704000
H	6.179163000	-2.664961000	-2.104054000

C	4.485296000	-4.746516000	-1.702529000
H	3.739697000	-4.525996000	-2.474219000
H	5.421992000	-5.018695000	-2.202003000
H	4.140146000	-5.619216000	-1.139050000
C	4.199666000	3.882390000	-1.717378000
H	3.849187000	3.609167000	-0.716633000
H	3.416075000	4.477633000	-2.198613000
H	5.085201000	4.516957000	-1.599098000
C	5.087787000	3.112780000	-3.911609000
H	5.977999000	3.730730000	-3.755060000
H	5.376159000	2.268849000	-4.547413000
H	4.354596000	3.718213000	-4.455291000
C	5.638528000	1.835414000	-1.846414000
H	5.314613000	1.513011000	-0.851266000
H	6.541326000	2.444176000	-1.722451000
H	5.906414000	0.942756000	-2.422280000
C	2.748585000	-1.961703000	-0.282895000
H	3.180025000	-1.236836000	-0.962487000
C	1.552230000	-1.632463000	0.368486000
C	1.593817000	-3.793566000	1.420583000
C	2.774483000	-4.105378000	0.762348000
C	2.675638000	1.279513000	-1.565422000
H	3.081227000	1.546088000	-0.595454000
C	2.729590000	1.444047000	-3.943682000
C	1.599563000	0.632691000	-4.011588000
C	1.539988000	0.470091000	-1.613313000
C	-0.210129000	-2.058868000	-2.497803000
C	-1.371353000	-2.819535000	-2.527402000
H	-1.345602000	-3.823957000	-2.113893000

C	-2.533369000	-1.026226000	-3.599263000
C	-1.372337000	-0.257287000	-3.573781000
C	-1.409666000	-3.048869000	1.838466000
H	-1.376242000	-3.886324000	1.146456000
C	-2.598261000	-2.743179000	2.496642000
H	-3.469088000	-3.359899000	2.302644000
C	-1.525583000	-0.898059000	3.577030000
H	-1.546321000	-0.050661000	4.256939000
C	-0.339111000	-1.192949000	2.918859000
H	0.542294000	-0.584190000	3.097471000
C	2.635971000	2.746742000	3.150786000
C	2.659331000	0.752587000	1.844510000
C	1.469016000	3.142141000	2.513325000
C	1.480383000	1.134538000	1.190160000
C	-0.392082000	3.106122000	-0.527249000
H	0.496072000	2.938263000	-1.129231000
C	-1.560518000	3.552903000	-1.139937000
H	-1.548762000	3.712221000	-2.212780000
C	-2.666923000	3.569328000	0.980782000
C	-1.503320000	3.130033000	1.599262000
H	-1.492648000	2.954231000	2.671885000
C	-4.134977000	-3.435902000	-1.482980000
H	-5.072088000	-3.996840000	-1.384498000
H	-4.221241000	-2.503462000	-0.913389000
H	-3.337852000	-4.033728000	-1.029548000
C	-5.052974000	-2.407299000	-3.550345000
H	-5.942689000	-3.039893000	-3.463292000
H	-4.915398000	-2.174416000	-4.611885000
H	-5.255431000	-1.473422000	-3.015537000

C	-3.802040000	-1.231295000	5.569854000
H	-3.537522000	-2.228589000	5.938809000
H	-3.013093000	-0.537796000	5.878170000
H	-4.729759000	-0.921163000	6.064700000
C	-5.145071000	-2.186579000	3.706504000
H	-5.354625000	-2.198374000	2.631497000
H	-4.939699000	-3.213215000	4.029408000
H	-6.054098000	-1.853299000	4.218441000
C	-4.372620000	0.170544000	3.566671000
H	-3.605454000	0.906012000	3.830914000
H	-4.492139000	0.184421000	2.478059000
H	-5.315297000	0.486852000	4.028854000
C	-3.910564000	4.379026000	-2.566930000
H	-3.642331000	3.426428000	-3.036334000
H	-4.871648000	4.697228000	-2.984382000
H	-3.162172000	5.126697000	-2.850822000
C	-4.431572000	5.606344000	-0.464838000
H	-4.580136000	5.554272000	0.618557000
H	-3.660925000	6.359168000	-0.664506000
H	-5.369750000	5.952160000	-0.914117000
C	4.306739000	0.974698000	5.014073000
H	5.229963000	0.686605000	5.529373000
H	3.951324000	1.908935000	5.460304000
H	3.554769000	0.201272000	5.204779000
H	1.146051000	-4.517514000	2.096339000
H	3.226439000	-5.079366000	0.928985000
H	3.063126000	3.392810000	3.912838000
H	1.006277000	4.089450000	2.777332000
H	3.105512000	-0.198529000	1.582573000

C	4.565608000	1.127349000	3.508340000
C	5.625437000	2.214872000	3.278572000
H	5.823155000	2.349815000	2.209308000
H	5.311864000	3.180232000	3.688247000
H	6.565991000	1.934391000	3.765817000
C	5.108220000	-0.198475000	2.970683000
H	4.406333000	-1.024325000	3.130136000
H	5.332067000	-0.141701000	1.899395000
H	6.038260000	-0.448638000	3.491225000
H	-3.548386000	3.743161000	1.592777000
C	-3.846458000	-3.141366000	-2.964206000
C	-3.659289000	-4.460879000	-3.726871000
H	-2.824947000	-5.046350000	-3.326850000
H	-4.564417000	-5.074430000	-3.650751000
H	-3.463424000	-4.275777000	-4.789138000
H	1.181204000	0.377022000	-4.981606000
H	3.163500000	1.811017000	-4.867961000
H	0.697294000	-2.479513000	-2.075484000
H	-3.430317000	-0.591646000	-4.026326000
H	-1.387995000	0.755937000	-3.967110000
C	-5.139607000	3.218903000	-0.748182000
H	-4.878638000	2.232787000	-1.147812000
H	-5.314187000	3.114201000	0.327981000
H	-6.082656000	3.537476000	-1.207128000
P	0.749212000	-0.029257000	-0.030887000
O	-3.538806000	0.090881000	-0.061049000
H	-3.729484000	0.964799000	0.301098000

4·CO2 E(RPBE1PBE) = -3070.32995334

Au	-1.321120000	-0.046311000	-0.188452000
P	0.919768000	-0.161087000	-0.005634000
O	-3.895321000	0.117110000	-2.326136000
O	-5.277284000	1.159481000	-0.843152000
C	3.600134000	-0.889599000	-3.035682000
C	1.632365000	1.214433000	0.971228000
C	0.873903000	-2.912596000	0.755993000
C	1.809498000	-0.052662000	-1.598808000
C	0.933159000	1.822192000	2.029615000
C	4.748577000	-1.855640000	-3.318993000
C	-0.382606000	1.332731000	2.504048000
C	1.551129000	-1.679249000	0.799237000
C	-2.650808000	4.907942000	-1.314331000
C	-4.313077000	0.510647000	-1.243071000
C	-1.540292000	3.914960000	-1.654141000
C	0.461106000	1.994754000	-2.251873000
C	3.485580000	2.794308000	1.216402000
C	1.509583000	0.971748000	-2.515661000
C	-0.375687000	-3.146123000	0.000880000
C	4.592336000	-2.494807000	3.031159000
C	3.289863000	-2.625985000	2.245509000
C	4.857823000	3.318371000	0.799106000
C	-2.938778000	0.345077000	3.250175000
C	-2.808286000	-3.594266000	-1.396377000
C	-4.132118000	-3.856336000	-2.112049000
C	-4.302448000	-0.235294000	3.625511000
C	-4.395674000	-5.369567000	-2.122428000
H	-4.455326000	-5.774557000	-1.106704000
H	-5.344916000	-5.585526000	-2.626202000

H	-3.599814000	-5.904017000	-2.652924000
C	-5.273201000	-3.145474000	-1.369711000
H	-5.373636000	-3.492439000	-0.336890000
H	-5.106069000	-2.064832000	-1.356133000
H	-6.225007000	-3.337615000	-1.877927000
C	-4.116109000	-3.345421000	-3.553237000
H	-3.933760000	-2.266325000	-3.586065000
H	-3.356790000	-3.850567000	-4.160013000
H	-5.088873000	-3.536707000	-4.018712000
C	5.605912000	-3.517394000	2.496707000
H	6.548632000	-3.437166000	3.049324000
H	5.241604000	-4.543981000	2.604117000
H	5.816875000	-3.343230000	1.435908000
C	4.314721000	-2.779104000	4.514195000
H	3.596296000	-2.060416000	4.923187000
H	3.909821000	-3.785080000	4.663098000
H	5.241856000	-2.701412000	5.093012000
C	5.201853000	-1.096576000	2.908009000
H	5.444139000	-0.849503000	1.868249000
H	4.532254000	-0.322251000	3.298282000
H	6.131502000	-1.052903000	3.484417000
C	4.953544000	-2.857380000	-2.181061000
H	5.192563000	-2.355769000	-1.236513000
H	4.069424000	-3.484159000	-2.021609000
H	5.789885000	-3.519823000	-2.426030000
C	4.447593000	-2.632394000	-4.608554000
H	4.328244000	-1.962282000	-5.465801000
H	5.267928000	-3.323026000	-4.833891000
H	3.527353000	-3.217702000	-4.506588000

C	6.045217000	-1.051255000	-3.495157000
H	6.883999000	-1.727764000	-3.693667000
H	5.977912000	-0.348576000	-4.331564000
H	6.276673000	-0.478078000	-2.590374000
C	5.800623000	3.281192000	2.010519000
H	6.790027000	3.659742000	1.730536000
H	5.427549000	3.898347000	2.833920000
H	5.920495000	2.257575000	2.382554000
C	4.714243000	4.765498000	0.305701000
H	4.307676000	5.420793000	1.082363000
H	5.692072000	5.162225000	0.010509000
H	4.049950000	4.819625000	-0.563930000
C	5.476662000	2.482308000	-0.323656000
H	5.623401000	1.439138000	-0.021862000
H	4.862658000	2.491919000	-1.231055000
H	6.457675000	2.892638000	-0.583717000
C	-3.397060000	5.301701000	-2.597294000
H	-4.196483000	6.014158000	-2.364062000
H	-2.719917000	5.773579000	-3.317779000
H	-3.856439000	4.434457000	-3.081300000
C	-3.635615000	4.244666000	-0.339770000
H	-4.090958000	3.342187000	-0.758260000
H	-3.129141000	3.964008000	0.590034000
H	-4.443096000	4.942493000	-0.088129000
C	-2.107261000	6.179271000	-0.661003000
H	-1.596709000	5.965159000	0.284499000
H	-1.406823000	6.708354000	-1.316568000
H	-2.935933000	6.860514000	-0.441086000
C	-5.451931000	0.705816000	3.259553000

H	-6.403738000	0.238753000	3.532889000
H	-5.387836000	1.659511000	3.793845000
H	-5.486406000	0.915692000	2.184089000
C	-4.332915000	-0.485411000	5.140479000
H	-4.194507000	0.449013000	5.695199000
H	-5.296722000	-0.916679000	5.434098000
H	-3.544992000	-1.181092000	5.446985000
C	-4.519011000	-1.565804000	2.887631000
H	-4.488234000	-1.425696000	1.802403000
H	-3.760091000	-2.308324000	3.151652000
H	-5.498045000	-1.981868000	3.150231000
C	-1.495041000	-3.677537000	0.653820000
H	-1.436195000	-3.901556000	1.715800000
C	-2.682888000	-3.890739000	-0.031513000
H	-3.531727000	-4.293650000	0.513865000
C	-1.680095000	-3.086229000	-2.047006000
H	-1.720909000	-2.845355000	-3.103561000
C	-0.484770000	-2.864900000	-1.364481000
H	0.375740000	-2.482588000	-1.907372000
C	1.414531000	-3.973397000	1.497712000
H	0.900873000	-4.930744000	1.481449000
C	2.588453000	-3.838726000	2.222348000
H	2.965985000	-4.695745000	2.773473000
C	2.739435000	-1.562960000	1.535053000
H	3.238101000	-0.602566000	1.556798000
C	2.837252000	-0.962285000	-1.870292000
H	3.051629000	-1.733371000	-1.140671000
C	3.286103000	0.126382000	-3.945628000
H	3.850469000	0.220412000	-4.869243000

C	2.264987000	1.034018000	-3.689806000
H	2.047859000	1.818416000	-4.409709000
C	0.785342000	3.197564000	-1.624668000
H	1.816084000	3.396993000	-1.347479000
C	-0.199123000	4.139211000	-1.330087000
H	0.098388000	5.056776000	-0.833449000
C	-0.876272000	1.771085000	-2.603410000
H	-1.160299000	0.846837000	-3.099483000
C	-1.853288000	2.716010000	-2.309418000
H	-2.879619000	2.487698000	-2.578447000
C	-2.774738000	1.648887000	2.767946000
H	-3.634283000	2.301181000	2.650286000
C	-1.519215000	2.137881000	2.406992000
H	-1.428523000	3.143658000	2.006011000
C	-0.530455000	0.041978000	3.029175000
H	0.343055000	-0.594335000	3.141368000
C	-1.782209000	-0.436279000	3.394283000
H	-1.857843000	-1.445644000	3.788902000
C	2.889843000	1.701041000	0.592830000
H	3.403046000	1.213915000	-0.227365000
C	2.760209000	3.417439000	2.240644000
H	3.171913000	4.285052000	2.749022000
C	1.517031000	2.941694000	2.634514000
H	0.984068000	3.433998000	3.443609000
O	-3.439087000	0.075118000	-0.112272000
H	-3.746386000	0.534828000	0.684003000

TS1 E(RPBE1PBE) = -3070.32280395

Au	1.313911000	0.340313000	-0.152605000

P	-0.908933000	0.068547000	0.007867000
O	3.436148000	0.967182000	-1.530670000
O	5.387843000	0.332443000	-0.539517000
C	-3.816593000	1.709365000	-2.398260000
C	-1.482089000	-1.642997000	0.332161000
C	-1.096643000	2.289481000	1.792515000
C	-1.881076000	0.542893000	-1.469426000
C	-0.700729000	-2.624123000	0.968861000
C	-5.078201000	2.553275000	-2.228335000
C	0.637695000	-2.394634000	1.558039000
C	-1.627542000	1.050372000	1.382762000
C	3.266224000	-3.265306000	-3.073505000
C	4.190800000	0.587191000	-0.611898000
C	1.979060000	-2.442897000	-3.070943000
C	-0.333366000	-0.810620000	-2.933076000
C	-3.294461000	-3.255490000	-0.043337000
C	-1.514498000	0.072149000	-2.743021000
C	0.024653000	2.981024000	1.117468000
C	-4.507061000	0.534769000	3.901874000
C	-3.305328000	1.128415000	3.170802000
C	-4.688263000	-3.598689000	-0.562960000
C	3.206581000	-2.076156000	2.739075000
C	2.178724000	4.352517000	-0.126838000
C	3.366656000	5.070623000	-0.764697000
C	4.585247000	-1.968692000	3.395434000
C	3.619722000	6.392846000	-0.025747000
H	3.848520000	6.231082000	1.032225000
H	4.471539000	6.916633000	-0.474166000
H	2.746127000	7.051481000	-0.085763000

C	4.609453000	4.173987000	-0.657347000
H	4.843493000	3.928306000	0.383529000
H	4.454470000	3.236496000	-1.199737000
H	5.480037000	4.684859000	-1.085190000
C	3.126188000	5.381857000	-2.242516000
H	2.977389000	4.469673000	-2.830063000
H	2.255971000	6.031708000	-2.387168000
H	3.999537000	5.900118000	-2.652210000
C	-5.668001000	1.539067000	3.853590000
H	-6.539916000	1.132869000	4.378465000
H	-5.404392000	2.488817000	4.329233000
H	-5.960879000	1.749607000	2.819059000
C	-4.125263000	0.264616000	5.364307000
H	-3.300727000	-0.453708000	5.427713000
H	-3.815750000	1.180772000	5.877353000
H	-4.981840000	-0.150288000	5.907373000
C	-4.976606000	-0.776949000	3.268734000
H	-5.285179000	-0.635015000	2.226951000
H	-4.198003000	-1.547128000	3.294904000
H	-5.840555000	-1.159275000	3.821826000
C	-5.331636000	2.921235000	-0.765151000
H	-5.467081000	2.031334000	-0.140059000
H	-4.512680000	3.513996000	-0.343297000
H	-6.245856000	3.519160000	-0.693082000
C	-4.934597000	3.847905000	-3.040873000
H	-4.791664000	3.643639000	-4.106675000
H	-5.836232000	4.461609000	-2.935351000
H	-4.079340000	4.436539000	-2.691697000
C	-6.285659000	1.755708000	-2.742908000

H	-7.202232000	2.345872000	-2.631850000
H	-6.180906000	1.496916000	-3.801058000
H	-6.409566000	0.824735000	-2.178374000
C	-5.538261000	-4.135078000	0.598311000
H	-6.540909000	-4.396716000	0.242093000
H	-5.096795000	-5.032505000	1.043214000
H	-5.643536000	-3.383756000	1.388872000
C	-4.567125000	-4.677585000	-1.648840000
H	-4.091861000	-5.586412000	-1.266461000
H	-5.560209000	-4.950702000	-2.022711000
H	-3.973451000	-4.315571000	-2.495359000
C	-5.396165000	-2.381829000	-1.163153000
H	-5.531859000	-1.583914000	-0.424265000
H	-4.847676000	-1.968327000	-2.016797000
H	-6.389046000	-2.676639000	-1.517760000
C	3.894204000	-3.229760000	-4.474106000
H	4.829639000	-3.801002000	-4.484635000
H	3.220064000	-3.667173000	-5.218890000
H	4.124914000	-2.206364000	-4.785964000
C	4.245987000	-2.651082000	-2.061640000
H	4.520064000	-1.627067000	-2.326719000
H	3.807653000	-2.625947000	-1.058188000
H	5.166770000	-3.244811000	-2.017591000
C	3.021058000	-4.724532000	-2.688035000
H	2.604184000	-4.813085000	-1.678650000
H	2.339350000	-5.223422000	-3.385603000
H	3.969963000	-5.270890000	-2.704063000
C	5.701753000	-2.037852000	2.342155000
H	6.676163000	-1.964497000	2.837512000

H	5.688722000	-2.980373000	1.786985000
H	5.643080000	-1.221950000	1.611642000
C	4.744650000	-3.150263000	4.364805000
H	4.672510000	-4.107304000	3.837546000
H	5.721794000	-3.107756000	4.859566000
H	3.968963000	-3.130332000	5.137923000
C	4.747379000	-0.663294000	4.177572000
H	4.602090000	0.214158000	3.536795000
H	4.047679000	-0.594598000	5.016749000
H	5.759894000	-0.609065000	4.590560000
C	1.155311000	3.364639000	1.851176000
H	1.213480000	3.118347000	2.908440000
C	2.209132000	4.027446000	1.237362000
H	3.078332000	4.289181000	1.834617000
C	1.038667000	3.985774000	-0.846706000
H	0.960602000	4.219205000	-1.902896000
C	-0.018869000	3.310269000	-0.240435000
H	-0.894784000	3.054045000	-0.829638000
C	-1.669996000	2.901723000	2.916147000
H	-1.266616000	3.854537000	3.248252000
C	-2.745298000	2.341815000	3.588821000
H	-3.157363000	2.865234000	4.447177000
C	-2.715778000	0.501358000	2.075827000
H	-3.105813000	-0.453444000	1.748843000
C	-3.014767000	1.347776000	-1.315626000
H	-3.276669000	1.685597000	-0.320193000
C	-3.429590000	1.253130000	-3.664199000
H	-4.017581000	1.516351000	-4.539337000
C	-2.303960000	0.454727000	-3.830754000

H	-2.031479000	0.106894000	-4.823579000
C	-0.408667000	-2.181610000	-2.683492000
H	-1.360126000	-2.627104000	-2.410263000
C	0.730369000	-2.982128000	-2.750326000
H	0.630266000	-4.039304000	-2.527517000
C	0.902570000	-0.271893000	-3.306821000
H	0.986250000	0.793204000	-3.504734000
C	2.032684000	-1.076126000	-3.374720000
H	2.981991000	-0.610104000	-3.614325000
C	2.958403000	-3.070943000	1.781310000
H	3.756733000	-3.741410000	1.477516000
C	1.704973000	-3.230417000	1.205740000
H	1.550437000	-4.005730000	0.460309000
C	0.874091000	-1.400210000	2.512067000
H	0.061681000	-0.750462000	2.826638000
C	2.133781000	-1.247854000	3.090322000
H	2.266922000	-0.463391000	3.827705000
C	-2.757158000	-1.976731000	-0.149222000
H	-3.338487000	-1.205499000	-0.638731000
C	-2.489266000	-4.234476000	0.553424000
H	-2.850494000	-5.254741000	0.648638000
C	-1.231373000	-3.921701000	1.044182000
H	-0.638733000	-4.695087000	1.525160000
O	3.427992000	0.442487000	0.597552000
H	3.845420000	-0.177165000	1.212459000

3 E(RPBE1PBE) = -3070.34706405

Au	-1.283395000	0.117044000	-0.218062000
P	0.953425000	-0.082195000	-0.011590000

O	-3.343194000	0.393843000	-0.351756000
O	-3.450532000	0.052957000	-2.595088000
C	3.393886000	-2.803511000	1.841179000
C	1.823238000	0.175354000	-1.607042000
C	1.131457000	1.604505000	2.277121000
C	1.580507000	-1.692662000	0.611871000
C	1.444046000	1.211138000	-2.484204000
C	4.749549000	-2.766273000	2.542619000
C	0.327458000	2.146157000	-2.195132000
C	1.750906000	1.138411000	1.103869000
C	-4.395105000	-3.716039000	-1.895730000
C	-3.919056000	0.343090000	-1.497161000
C	-3.014321000	-3.458716000	-1.293270000
C	-0.461846000	-3.058065000	-0.113325000
C	3.607034000	-0.525062000	-3.130618000
C	0.861887000	-2.899932000	0.527593000
C	-0.177558000	1.101837000	2.749245000
C	5.013625000	3.194386000	1.031560000
C	3.656745000	2.637450000	1.455476000
C	4.794789000	-1.421256000	-3.475055000
C	-1.906170000	3.773944000	-1.559746000
C	-2.744399000	0.146150000	3.490853000
C	-4.155914000	-0.338178000	3.816989000
C	-3.129044000	4.570786000	-1.109463000
C	-4.585561000	0.234441000	5.175548000
H	-4.571756000	1.329211000	5.172060000
H	-5.604900000	-0.087074000	5.418145000
H	-3.921038000	-0.109568000	5.975858000
C	-5.121827000	0.151513000	2.726690000

H	-5.173813000	1.244278000	2.690925000
H	-4.808426000	-0.195559000	1.737039000
H	-6.132459000	-0.222635000	2.927311000
C	-4.240170000	-1.863987000	3.878172000
H	-3.951897000	-2.319744000	2.924655000
H	-3.603987000	-2.280415000	4.666814000
H	-5.271032000	-2.164524000	4.093263000
C	6.035451000	2.929213000	2.146745000
H	7.014565000	3.333380000	1.865840000
H	5.736802000	3.399157000	3.089149000
H	6.149576000	1.854432000	2.326459000
C	4.886267000	4.707453000	0.803107000
H	4.160509000	4.923818000	0.011515000
H	4.563403000	5.228560000	1.709828000
H	5.853133000	5.126520000	0.502721000
C	5.523970000	2.549955000	-0.259467000
H	5.665173000	1.469230000	-0.146995000
H	4.842615000	2.720083000	-1.100538000
H	6.493298000	2.984423000	-0.524166000
C	5.377355000	-1.371026000	2.511972000
H	5.555299000	-1.026769000	1.487034000
H	4.750674000	-0.629615000	3.019634000
H	6.344033000	-1.396298000	3.025486000
C	4.567102000	-3.185390000	4.008523000
H	4.158804000	-4.197391000	4.092541000
H	5.531385000	-3.168204000	4.528656000
H	3.887261000	-2.501760000	4.528463000
C	5.702917000	-3.746815000	1.844097000
H	6.682833000	-3.734355000	2.334340000

H	5.324904000	-4.773550000	1.878796000
H	5.844943000	-3.475036000	0.792166000
C	6.038765000	-0.544084000	-3.680658000
H	6.905466000	-1.169580000	-3.922119000
H	5.903216000	0.169149000	-4.499683000
H	6.271675000	0.024375000	-2.773373000
C	4.490795000	-2.189523000	-4.768970000
H	4.306438000	-1.510698000	-5.607745000
H	5.338548000	-2.830317000	-5.036242000
H	3.607204000	-2.825387000	-4.648012000
C	5.096006000	-2.430227000	-2.365251000
H	5.340833000	-1.931175000	-1.421023000
H	4.254643000	-3.108237000	-2.185712000
H	5.958502000	-3.040748000	-2.651326000
C	-4.643725000	-5.231913000	-1.925506000
H	-5.628842000	-5.446060000	-2.355958000
H	-3.888954000	-5.741448000	-2.534555000
H	-4.615470000	-5.665640000	-0.920500000
C	-5.475428000	-3.047195000	-1.032984000
H	-5.464611000	-3.415231000	-0.002540000
H	-5.345870000	-1.961250000	-1.003272000
H	-6.467344000	-3.257780000	-1.449445000
C	-4.513376000	-3.170745000	-3.319690000
H	-4.327162000	-2.092063000	-3.347496000
H	-3.813268000	-3.662885000	-4.003778000
H	-5.524952000	-3.355849000	-3.697110000
C	-4.336156000	4.343083000	-2.020713000
H	-5.176696000	4.951202000	-1.669665000
H	-4.126341000	4.631758000	-3.056494000

H	-4.659844000	3.297052000	-2.010213000
C	-2.803056000	6.070253000	-1.097329000
H	-2.542593000	6.423909000	-2.101144000
H	-3.671030000	6.642626000	-0.750672000
H	-1.965941000	6.300197000	-0.430814000
C	-3.503339000	4.110684000	0.308761000
H	-3.703768000	3.034193000	0.323598000
H	-2.696896000	4.313429000	1.020587000
H	-4.400076000	4.637837000	0.655429000
C	-1.249598000	1.985008000	2.927016000
H	-1.103884000	3.045617000	2.740052000
C	-2.504049000	1.513207000	3.288570000
H	-3.318680000	2.225003000	3.388409000
C	-1.655420000	-0.720441000	3.367196000
H	-1.781317000	-1.784241000	3.535077000
C	-0.393363000	-0.253970000	3.005872000
H	0.430097000	-0.956166000	2.915553000
C	1.783443000	2.612580000	3.000319000
H	1.311936000	2.991502000	3.903195000
C	3.013653000	3.116997000	2.604542000
H	3.479992000	3.893777000	3.204336000
C	2.996525000	1.655056000	0.722262000
H	3.448111000	1.286216000	-0.190249000
C	2.822724000	-1.672837000	1.265286000
H	3.350244000	-0.730473000	1.333354000
C	2.661319000	-3.994942000	1.763260000
H	3.054723000	-4.907672000	2.202257000
C	1.431250000	-4.034566000	1.127384000
H	0.887981000	-4.973893000	1.072120000

C	-0.704631000	-2.699246000	-1.442249000
H	0.097807000	-2.276253000	-2.041571000
C	-1.958043000	-2.902045000	-2.019567000
H	-2.104783000	-2.600269000	-3.049732000
C	-1.512377000	-3.630951000	0.615355000
H	-1.349453000	-3.915629000	1.651586000
C	-2.759740000	-3.819601000	0.037222000
H	-3.550216000	-4.261976000	0.637243000
C	-1.941113000	2.882768000	-2.633683000
H	-2.847609000	2.758563000	-3.213687000
C	-0.845051000	2.083576000	-2.949388000
H	-0.922963000	1.362355000	-3.757635000
C	0.397352000	3.089230000	-1.161428000
H	1.311369000	3.192816000	-0.586551000
C	-0.701142000	3.879404000	-0.850319000
H	-0.619061000	4.578113000	-0.021931000
C	2.886753000	-0.669721000	-1.945095000
H	3.158055000	-1.457056000	-1.253519000
C	3.203681000	0.490608000	-4.005070000
H	3.724931000	0.636083000	-4.947297000
C	2.144845000	1.331675000	-3.688888000
H	1.856279000	2.117873000	-4.381424000
O	-5.241810000	0.666376000	-1.390118000
H	-5.597499000	0.588862000	-2.286499000

8 E(RPBE1PBE) = -3281.27533629

Au	1.136934000	0.051804000	0.104533000
C	-3.840709000	2.861419000	1.058694000
C	-3.898398000	-2.558120000	1.704961000

C	-1.467563000	1.748581000	2.174241000
C	-0.244123000	1.197818000	2.800748000
C	2.161136000	0.133274000	3.864839000
C	-0.177665000	2.084896000	-2.200073000
C	-1.265856000	1.148082000	-2.577955000
C	-3.324228000	-0.622239000	-3.444220000
C	3.453974000	-0.492548000	4.386533000
C	1.982819000	3.770167000	-1.455276000
C	-5.117755000	3.465398000	0.479633000
C	-4.435990000	-1.591253000	-3.839738000
C	3.994826000	-4.134537000	-1.549294000
C	2.619498000	-3.736414000	-1.014739000
C	-1.282707000	-2.838503000	0.603717000
C	0.061887000	-3.094191000	0.040495000
C	-6.252990000	3.312860000	1.502198000
H	-7.175370000	3.758364000	1.112846000
H	-6.449416000	2.256117000	1.715029000
H	-6.014450000	3.808899000	2.448170000
C	-5.540416000	2.782389000	-0.822562000
H	-4.777065000	2.879127000	-1.602549000
H	-5.746634000	1.716291000	-0.676526000
H	-6.457896000	3.247830000	-1.197199000
C	-4.882407000	4.955731000	0.193170000
H	-4.071313000	5.091024000	-0.530705000
H	-5.789594000	5.408282000	-0.222833000
H	-4.619993000	5.507390000	1.101519000
C	-3.940427000	-3.033565000	-3.654452000
H	-3.655952000	-3.237108000	-2.617029000
H	-3.069806000	-3.233068000	-4.288572000

H	-4.731032000	-3.741139000	-3.928468000
C	-4.866214000	-1.406413000	-5.294661000
H	-5.660648000	-2.120258000	-5.535322000
H	-5.257458000	-0.399965000	-5.478481000
H	-4.037217000	-1.584843000	-5.988006000
C	-5.653442000	-1.352613000	-2.933171000
H	-5.408087000	-1.510211000	-1.877628000
H	-6.461633000	-2.044466000	-3.195611000
H	-6.031096000	-0.330149000	-3.043637000
C	-3.165991000	1.812054000	0.438817000
H	-3.543633000	1.417298000	-0.497225000
C	-1.992260000	1.257368000	0.965842000
C	-2.131073000	2.821153000	2.783983000
C	-3.287470000	3.365158000	2.243767000
C	-2.801308000	-0.681761000	-2.148045000
H	-3.190714000	-1.426610000	-1.462643000
C	-2.787106000	0.335030000	-4.304629000
C	-1.778909000	1.195916000	-3.875612000
C	-1.792588000	0.171771000	-1.701521000
C	-0.331500000	3.051808000	-1.198179000
C	0.729783000	3.870262000	-0.834857000
H	0.575344000	4.596420000	-0.041029000
C	2.109128000	2.846101000	-2.494861000
C	1.049685000	2.021038000	-2.863824000
C	0.875079000	2.009443000	2.998442000
H	0.834305000	3.054908000	2.704590000
C	2.057332000	1.482899000	3.514074000
H	2.911734000	2.142891000	3.619423000
C	1.014384000	-0.659754000	3.716251000

H	1.042704000	-1.708066000	4.000201000
C	-0.164598000	-0.144238000	3.193766000
H	-1.031587000	-0.789004000	3.082059000
C	-3.231085000	-3.789592000	1.724802000
C	-3.217562000	-1.478630000	1.149193000
C	-1.959179000	-3.919725000	1.190150000
C	-1.929371000	-1.589161000	0.603762000
C	0.377764000	-2.822681000	-1.293196000
H	-0.371162000	-2.381190000	-1.945677000
C	1.632440000	-3.145420000	-1.808983000
H	1.829983000	-2.930821000	-2.853725000
C	2.297633000	-3.988384000	0.326280000
C	1.045289000	-3.687534000	0.841848000
H	0.824920000	-3.900366000	1.884616000
C	3.409738000	4.356009000	0.503244000
H	4.245371000	4.967824000	0.862938000
H	3.664505000	3.301784000	0.653634000
H	2.536352000	4.583572000	1.122719000
C	4.428164000	4.366179000	-1.762273000
H	5.230177000	5.010358000	-1.386012000
H	4.309930000	4.575620000	-2.831031000
H	4.753334000	3.326909000	-1.647117000
C	3.230458000	-0.988892000	5.822567000
H	2.966280000	-0.159968000	6.488378000
H	2.426032000	-1.729979000	5.869939000
H	4.142846000	-1.458221000	6.208227000
C	4.615311000	0.502460000	4.388900000
H	4.822372000	0.885461000	3.383188000
H	4.424104000	1.356253000	5.047873000

H	5.522263000	0.004734000	4.747696000
C	3.838726000	-1.681664000	3.492442000
H	3.076534000	-2.465968000	3.513911000
H	3.963283000	-1.369069000	2.450484000
H	4.781182000	-2.121383000	3.838462000
C	4.152330000	-3.814246000	-3.035440000
H	4.054125000	-2.741655000	-3.226853000
H	5.149005000	-4.120074000	-3.371016000
H	3.417254000	-4.347938000	-3.648008000
C	4.185150000	-5.647366000	-1.358178000
H	4.135424000	-5.933180000	-0.302699000
H	3.416664000	-6.212499000	-1.897398000
H	5.164853000	-5.955203000	-1.741408000
C	-5.275281000	-2.804850000	3.770162000
H	-6.280659000	-2.729073000	4.199651000
H	-4.923976000	-3.830490000	3.920973000
H	-4.613939000	-2.134744000	4.330171000
H	-1.730866000	3.220835000	3.712047000
H	-3.768271000	4.191616000	2.760237000
H	-3.711544000	-4.663570000	2.156466000
H	-1.469615000	-4.889820000	1.202228000
H	-3.693846000	-0.506689000	1.147211000
C	-5.305457000	-2.426778000	2.282237000
C	-6.246850000	-3.380330000	1.532239000
H	-6.288413000	-3.133074000	0.465832000
H	-5.925473000	-4.422574000	1.624435000
H	-7.261865000	-3.305215000	1.938117000
C	-5.851686000	-1.003132000	2.153711000
H	-5.232939000	-0.277319000	2.692440000

H	-5.918524000	-0.687231000	1.106430000
H	-6.860230000	-0.959819000	2.577145000
H	3.037600000	-4.436715000	0.983712000
C	3.142851000	4.643939000	-0.981834000
C	2.771971000	6.123846000	-1.155538000
H	1.878010000	6.387404000	-0.580996000
H	3.592357000	6.763329000	-0.809783000
H	2.578578000	6.358913000	-2.207849000
H	-1.380837000	1.937207000	-4.563654000
H	-3.148866000	0.424820000	-5.323576000
H	-1.286102000	3.161824000	-0.695973000
H	3.054391000	2.731828000	-3.012474000
H	1.190458000	1.288651000	-3.654493000
C	5.085364000	-3.383577000	-0.771095000
H	4.987633000	-2.303063000	-0.910003000
H	5.034531000	-3.594244000	0.301954000
H	6.076894000	-3.687401000	-1.126671000
P	-1.134737000	-0.047031000	-0.003038000
N	3.154497000	0.216953000	0.293517000
H	3.486385000	0.517269000	1.201239000
C	4.109635000	0.281917000	-0.670528000
C	5.442754000	0.667919000	-0.377638000
C	3.828951000	-0.050050000	-2.018668000
C	6.416737000	0.719873000	-1.367462000
H	5.696834000	0.928568000	0.648571000
C	4.810578000	0.011276000	-2.997953000
H	2.815637000	-0.352380000	-2.269404000
C	6.119141000	0.396571000	-2.692657000
H	7.426415000	1.022404000	-1.096722000

H	4.548306000	-0.248363000	-4.021902000
H	6.882521000	0.441778000	-3.463555000

8·CO2 E(RPBE1PBE) = -3281.28271948

Au	1.112279000	0.370662000	0.173424000
P	-1.137672000	0.042709000	0.107558000
O	3.069607000	1.569558000	2.336297000
O	5.054895000	0.994969000	1.408691000
C	-3.697829000	-3.143271000	0.047088000
C	-1.971206000	1.194102000	1.266976000
C	-1.540536000	-0.319307000	-2.662936000
C	-1.828550000	-1.612932000	0.476442000
C	-1.520537000	2.512641000	1.485594000
C	-5.069081000	-3.442035000	-0.553959000
C	-0.353800000	3.150655000	0.824741000
C	-1.917274000	0.399156000	-1.515604000
C	3.961466000	-2.036495000	3.991679000
C	3.847531000	1.229947000	1.441112000
C	2.633329000	-2.123313000	3.240827000
C	0.152259000	-2.399191000	1.882023000
C	-3.799225000	1.499359000	2.875190000
C	-1.148242000	-2.599345000	1.210997000
C	-0.474233000	-1.354401000	-2.645716000
C	-4.837197000	2.548120000	-2.821071000
C	-3.674977000	1.559574000	-2.759274000
C	-5.031438000	0.978037000	3.611145000
C	1.855199000	4.518845000	-0.336483000
C	1.544558000	-3.345027000	-2.606711000
C	2.662049000	-4.387187000	-2.580990000

C	3.035618000	5.303869000	-0.909753000
C	3.546852000	-4.216311000	-3.824924000
H	4.014379000	-3.227228000	-3.857197000
H	4.348140000	-4.964051000	-3.822466000
H	2.963926000	-4.345788000	-4.743490000
C	3.515722000	-4.177559000	-1.322060000
H	3.931410000	-3.166993000	-1.282516000
H	2.918526000	-4.326754000	-0.417462000
H	4.348882000	-4.890016000	-1.303972000
C	2.118884000	-5.816377000	-2.565041000
H	1.509997000	-6.009583000	-1.675121000
H	1.510962000	-6.031816000	-3.450543000
H	2.953803000	-6.524867000	-2.554160000
C	-6.101150000	1.808431000	-3.283342000
H	-6.946307000	2.503620000	-3.338115000
H	-5.970624000	1.362082000	-4.274061000
H	-6.364158000	1.007104000	-2.583842000
C	-4.497820000	3.659466000	-3.824875000
H	-3.597391000	4.202641000	-3.517693000
H	-4.325475000	3.259192000	-4.828980000
H	-5.323794000	4.376752000	-3.886793000
C	-5.120111000	3.189642000	-1.461293000
H	-5.389447000	2.442930000	-0.705940000
H	-4.260416000	3.757321000	-1.088499000
H	-5.960636000	3.884825000	-1.554612000
C	-5.643973000	-2.239107000	-1.304986000
H	-5.783233000	-1.374966000	-0.645841000
H	-5.003436000	-1.932808000	-2.139610000
H	-6.623753000	-2.501263000	-1.717173000

C	-4.944299000	-4.614574000	-1.537544000
H	-4.574963000	-5.519933000	-1.045861000
H	-5.922455000	-4.846741000	-1.973324000
H	-4.258011000	-4.369939000	-2.355748000
C	-6.037214000	-3.823811000	0.574975000
H	-7.025726000	-4.055959000	0.163076000
H	-5.687816000	-4.703154000	1.125317000
H	-6.150394000	-3.001375000	1.289793000
C	-6.227333000	1.886793000	3.290871000
H	-7.121519000	1.531291000	3.814975000
H	-6.047377000	2.921125000	3.599986000
H	-6.441281000	1.887741000	2.216241000
C	-4.761654000	0.998891000	5.122526000
H	-4.540171000	2.009536000	5.480125000
H	-5.639709000	0.635182000	5.668032000
H	-3.912105000	0.356007000	5.376970000
C	-5.387967000	-0.451288000	3.197444000
H	-5.610562000	-0.517336000	2.126680000
H	-4.582625000	-1.158172000	3.424867000
H	-6.279458000	-0.776882000	3.743085000
C	4.078299000	-3.256386000	4.918704000
H	5.018402000	-3.214960000	5.480954000
H	3.252706000	-3.283920000	5.638621000
H	4.065895000	-4.195217000	4.354998000
C	5.130391000	-2.043284000	2.994980000
H	5.106431000	-2.920445000	2.340040000
H	5.119605000	-1.140572000	2.375453000
H	6.081446000	-2.065120000	3.540422000
C	4.061434000	-0.764446000	4.835649000

H	3.954354000	0.133907000	4.218112000
H	3.299504000	-0.739177000	5.623003000
H	5.041000000	-0.728976000	5.324615000
C	4.357461000	4.649312000	-0.481021000
H	5.203952000	5.249379000	-0.833551000
H	4.439177000	4.564377000	0.606841000
H	4.462736000	3.645900000	-0.906777000
C	2.985003000	6.738570000	-0.361245000
H	3.063767000	6.757392000	0.730174000
H	3.815997000	7.326321000	-0.767387000
H	2.048795000	7.233288000	-0.642038000
C	2.999030000	5.374330000	-2.436596000
H	3.046283000	4.381029000	-2.895035000
H	2.096227000	5.877429000	-2.799122000
H	3.862904000	5.943114000	-2.795890000
C	0.878511000	-1.004643000	-2.712356000
H	1.160747000	0.041720000	-2.788705000
C	1.865308000	-1.982791000	-2.692283000
H	2.903344000	-1.670019000	-2.738963000
C	0.189901000	-3.684952000	-2.553203000
H	-0.112309000	-4.725186000	-2.493702000
C	-0.803775000	-2.707864000	-2.574967000
H	-1.847981000	-3.002627000	-2.522415000
C	-2.243255000	-0.074224000	-3.846748000
H	-1.965610000	-0.624800000	-4.741505000
C	-3.283812000	0.845561000	-3.898116000
H	-3.801194000	0.999216000	-4.841143000
C	-2.972169000	1.316153000	-1.579689000
H	-3.257092000	1.838348000	-0.674597000

C	-3.085572000	-1.900939000	-0.078406000
H	-3.590171000	-1.121963000	-0.636102000
C	-2.991601000	-4.131079000	0.747117000
H	-3.417327000	-5.123765000	0.866224000
C	-1.755486000	-3.861790000	1.311229000
H	-1.243294000	-4.638985000	1.871448000
C	0.387709000	-1.322582000	2.742917000
H	-0.399161000	-0.595036000	2.927005000
C	1.606377000	-1.190143000	3.406915000
H	1.748178000	-0.333420000	4.054398000
C	1.178555000	-3.336556000	1.709087000
H	1.024926000	-4.176821000	1.037136000
C	2.390271000	-3.196280000	2.370952000
H	3.160775000	-3.945221000	2.208002000
C	1.736351000	4.362956000	1.052277000
H	2.503661000	4.762339000	1.708503000
C	0.656235000	3.704761000	1.621491000
H	0.604296000	3.592995000	2.700305000
C	-0.240926000	3.294755000	-0.562376000
H	-1.016533000	2.903429000	-1.212210000
C	0.842885000	3.965263000	-1.128850000
H	0.877866000	4.065636000	-2.208978000
C	-3.090590000	0.719406000	1.964057000
H	-3.409616000	-0.299231000	1.788108000
C	-3.333927000	2.800511000	3.095638000
H	-3.843619000	3.450929000	3.801188000
C	-2.224130000	3.285798000	2.420696000
H	-1.891894000	4.304249000	2.602513000
N	3.120450000	1.130273000	0.065204000

H	2.835189000	2.097670000	-0.115440000
C	3.856978000	0.658979000	-1.066941000
C	3.701727000	1.302304000	-2.296800000
C	4.691830000	-0.456083000	-0.972879000
C	4.388286000	0.850246000	-3.420449000
H	3.027759000	2.152050000	-2.370751000
C	5.389456000	-0.891135000	-2.096372000
H	4.798675000	-0.967051000	-0.024363000
C	5.245139000	-0.244039000	-3.323640000
H	4.254787000	1.361019000	-4.370076000
H	6.045983000	-1.752757000	-2.011318000
H	5.790823000	-0.593380000	-4.195327000

TS2 E(RPBE1PBE) = -3281.27533629

Au	1.034864000	0.301228000	0.472144000
P	-1.165685000	-0.003781000	0.109207000
O	2.675343000	0.920408000	2.090374000
O	4.860041000	1.169444000	1.570523000
C	-3.733538000	-3.149442000	-0.402831000
C	-2.093408000	1.060518000	1.283667000
C	-1.283773000	-0.081567000	-2.709087000
C	-1.894083000	-1.677932000	0.287850000
C	-1.645983000	2.350481000	1.639430000
C	-5.063910000	-3.377482000	-1.116776000
C	-0.450133000	3.032671000	1.080644000
C	-1.811119000	0.484179000	-1.536937000
C	3.702292000	-2.370555000	4.020282000
C	3.662341000	1.090073000	1.319966000
C	2.386384000	-2.433769000	3.246442000

C	-0.021447000	-2.602212000	1.753958000
C	-4.003548000	1.250702000	2.810900000
C	-1.276874000	-2.730642000	0.984781000
C	-0.186272000	-1.085472000	-2.692976000
C	-4.661029000	2.688280000	-2.905852000
C	-3.472802000	1.732461000	-2.826917000
C	-5.278296000	0.684717000	3.431646000
C	1.782042000	4.505912000	0.103550000
C	1.890934000	-3.015642000	-2.692949000
C	3.039802000	-4.023106000	-2.680758000
C	2.960706000	5.356997000	-0.369952000
C	3.940448000	-3.787413000	-3.902461000
H	4.377045000	-2.783870000	-3.897333000
H	4.765255000	-4.509205000	-3.907326000
H	3.378219000	-3.907822000	-4.835074000
C	3.863564000	-3.823742000	-1.400192000
H	4.258998000	-2.806267000	-1.332886000
H	3.250814000	-4.002123000	-0.511082000
H	4.710270000	-4.520057000	-1.378928000
C	2.541898000	-5.468257000	-2.718866000
H	1.929012000	-5.712043000	-1.844314000
H	1.951180000	-5.671896000	-3.618729000
H	3.398452000	-6.150549000	-2.721370000
C	-5.828718000	1.982960000	-3.610695000
H	-6.689360000	2.657475000	-3.680857000
H	-5.562895000	1.673814000	-4.626358000
H	-6.139559000	1.090707000	-3.055948000
C	-4.250296000	3.929545000	-3.711129000
H	-3.418259000	4.451928000	-3.226400000

H	-3.938837000	3.666620000	-4.727056000
H	-5.092355000	4.626588000	-3.787388000
C	-5.131638000	3.138488000	-1.521455000
H	-5.452253000	2.291156000	-0.904934000
H	-4.350141000	3.680459000	-0.977570000
H	-5.987265000	3.812839000	-1.629764000
C	-5.562980000	-2.115112000	-1.823475000
H	-5.736577000	-1.293833000	-1.119218000
H	-4.857726000	-1.767638000	-2.586821000
H	-6.514004000	-2.328782000	-2.321960000
C	-4.896520000	-4.486446000	-2.165275000
H	-4.573897000	-5.429089000	-1.712536000
H	-5.849289000	-4.669506000	-2.674402000
H	-4.156346000	-4.202809000	-2.921468000
C	-6.113911000	-3.808165000	-0.081820000
H	-7.078208000	-3.985417000	-0.571312000
H	-5.821462000	-4.731626000	0.428191000
H	-6.256091000	-3.033304000	0.679637000
C	-6.444195000	1.642100000	3.144473000
H	-7.369187000	1.251776000	3.583409000
H	-6.267236000	2.636064000	3.567010000
H	-6.601159000	1.756685000	2.066162000
C	-5.083492000	0.555666000	4.949127000
H	-4.859280000	1.522485000	5.410744000
H	-5.994245000	0.163828000	5.415804000
H	-4.260108000	-0.128771000	5.180195000
C	-5.633179000	-0.691307000	2.864216000
H	-5.802440000	-0.649908000	1.782486000
H	-4.850457000	-1.431940000	3.060790000

H	-6.555568000	-1.051713000	3.331112000
C	3.909147000	-3.702834000	4.756576000
H	4.846021000	-3.680211000	5.325190000
H	3.090084000	-3.895630000	5.458393000
H	3.960762000	-4.545469000	4.059202000
C	4.867656000	-2.141127000	3.045567000
H	4.913563000	-2.914778000	2.272240000
H	4.783836000	-1.165851000	2.555592000
H	5.817973000	-2.161802000	3.591986000
C	3.712335000	-1.234768000	5.044251000
H	3.567369000	-0.264764000	4.557705000
H	2.937069000	-1.363118000	5.807715000
H	4.679862000	-1.213688000	5.557222000
C	4.286140000	4.687668000	0.021086000
H	5.126167000	5.339920000	-0.242987000
H	4.343687000	4.486849000	1.094998000
H	4.425219000	3.738662000	-0.506499000
C	2.873820000	6.733020000	0.309368000
H	2.934511000	6.650432000	1.398948000
H	3.700293000	7.370449000	-0.024686000
H	1.933047000	7.235720000	0.059705000
C	2.951443000	5.567890000	-1.884318000
H	3.018688000	4.620559000	-2.429671000
H	2.049591000	6.092503000	-2.217745000
H	3.815170000	6.175607000	-2.173192000
C	1.155820000	-0.694312000	-2.739522000
H	1.408312000	0.361676000	-2.776962000
C	2.170951000	-1.642234000	-2.740333000
H	3.200110000	-1.298747000	-2.772123000

C	0.547237000	-3.396901000	-2.654990000
H	0.275903000	-4.446942000	-2.624260000
C	-0.475353000	-2.449376000	-2.656625000
H	-1.510395000	-2.776863000	-2.620694000
C	-1.859548000	0.288430000	-3.928938000
H	-1.462736000	-0.141627000	-4.844712000
C	-2.925892000	1.177070000	-3.990103000
H	-3.341833000	1.431026000	-4.961328000
C	-2.892131000	1.370080000	-1.612138000
H	-3.291249000	1.774980000	-0.689972000
C	-3.105675000	-1.908302000	-0.383048000
H	-3.560578000	-1.084644000	-0.918489000
C	-3.087505000	-4.199462000	0.263953000
H	-3.525881000	-5.193718000	0.270405000
C	-1.892996000	-3.990801000	0.934446000
H	-1.421249000	-4.816938000	1.459485000
C	0.118013000	-1.675639000	2.791222000
H	-0.709975000	-1.015577000	3.038794000
C	1.299842000	-1.600700000	3.525419000
H	1.369296000	-0.859613000	4.313000000
C	1.053279000	-3.457806000	1.484300000
H	0.967331000	-4.183670000	0.680399000
C	2.231412000	-3.367917000	2.212989000
H	3.048868000	-4.039582000	1.964989000
C	1.630734000	4.222665000	1.469718000
H	2.377783000	4.568976000	2.178356000
C	0.539494000	3.512937000	1.949622000
H	0.457878000	3.308311000	3.013424000
C	-0.299966000	3.299094000	-0.284026000

H	-1.057756000	2.968411000	-0.985742000
C	0.795044000	4.019358000	-0.760404000
H	0.855319000	4.215992000	-1.825913000
C	-3.255050000	0.543538000	1.871558000
H	-3.576193000	-0.449718000	1.586191000
C	-3.536324000	2.518553000	3.174310000
H	-4.075133000	3.109342000	3.910050000
C	-2.388961000	3.047914000	2.602059000
H	-2.058234000	4.042194000	2.889952000
N	3.197208000	1.190600000	-0.090328000
H	2.765199000	2.105824000	-0.222700000
C	4.052003000	0.874953000	-1.162044000
C	3.882201000	1.526565000	-2.388711000
C	5.017295000	-0.131472000	-1.048830000
C	4.659173000	1.174164000	-3.487486000
H	3.125480000	2.302554000	-2.476996000
C	5.798507000	-0.468221000	-2.150153000
H	5.151552000	-0.639245000	-0.100950000
C	5.624779000	0.174865000	-3.375600000
H	4.508997000	1.686996000	-4.433615000
H	6.546059000	-1.250440000	-2.048771000
H	6.235538000	-0.098201000	-4.231031000

9 E(RPBE1PBE) = -3281.30257285

Au	-0.763361000	-0.065901000	0.136072000
P	1.481529000	-0.041245000	-0.050536000
O	-2.815958000	0.012157000	0.436142000
O	-3.296537000	0.354660000	-1.753213000
C	4.435291000	-2.802668000	0.620070000

C	2.047260000	0.723927000	-1.620696000
C	1.870223000	0.982444000	2.574841000
C	2.345449000	-1.661699000	0.014949000
C	1.429922000	1.876943000	-2.148501000
C	5.881020000	-2.769137000	1.109647000
C	0.296367000	2.576037000	-1.494212000
C	2.334945000	0.935283000	1.248135000
C	-3.806978000	-3.611912000	-2.011465000
C	-3.605069000	0.253487000	-0.565224000
C	-2.347690000	-3.401337000	-1.608540000
C	0.345136000	-3.046334000	-0.778563000
C	3.592688000	0.624984000	-3.520260000
C	1.739718000	-2.889833000	-0.311788000
C	0.705085000	0.203502000	3.051046000
C	5.324591000	3.370815000	1.340885000
C	4.118146000	2.534770000	1.761348000
C	4.765004000	-0.019419000	-4.256728000
C	-1.944103000	3.774772000	-0.231393000
C	-1.601878000	-1.244250000	3.849848000
C	-2.883765000	-1.981241000	4.234770000
C	-3.151398000	4.333484000	0.519768000
C	-3.157743000	-1.774536000	5.731451000
H	-3.268602000	-0.713846000	5.978582000
H	-4.083396000	-2.284870000	6.021630000
H	-2.341493000	-2.179759000	6.339652000
C	-4.056221000	-1.415744000	3.417279000
H	-4.222537000	-0.354571000	3.630040000
H	-3.864767000	-1.515124000	2.344324000
H	-4.978664000	-1.954926000	3.662624000

C	-2.791000000	-3.482307000	3.957608000
H	-2.619405000	-3.685330000	2.895024000
H	-1.989643000	-3.957138000	4.534001000
H	-3.732359000	-3.964432000	4.241571000
C	6.536736000	2.967170000	2.193000000
H	7.411963000	3.563579000	1.911835000
H	6.352027000	3.125830000	3.260218000
H	6.783975000	1.909914000	2.046216000
C	5.009334000	4.856665000	1.568364000
H	4.142037000	5.166960000	0.975317000
H	4.793643000	5.070498000	2.619911000
H	5.864400000	5.473756000	1.270457000
C	5.678011000	3.172295000	-0.134815000
H	5.941832000	2.131982000	-0.355975000
H	4.854591000	3.463534000	-0.796470000
H	6.543128000	3.793423000	-0.388371000
C	6.360617000	-1.344257000	1.394306000
H	6.329776000	-0.717971000	0.495797000
H	5.760685000	-0.859225000	2.172089000
H	7.397844000	-1.372456000	1.743502000
C	5.991974000	-3.587481000	2.403959000
H	5.695731000	-4.629882000	2.250657000
H	7.026176000	-3.582913000	2.766350000
H	5.354129000	-3.166299000	3.188587000
C	6.789631000	-3.384903000	0.035708000
H	7.832521000	-3.372680000	0.371866000
H	6.519368000	-4.423938000	-0.177118000
H	6.727492000	-2.821395000	-0.901777000
C	5.877564000	1.023533000	-4.439156000

H	6.729286000	0.578545000	-4.965595000
H	5.536659000	1.883729000	-5.023612000
H	6.231057000	1.392689000	-3.469984000
C	4.287720000	-0.505542000	-5.632614000
H	3.894645000	0.317811000	-6.237465000
H	5.119046000	-0.959919000	-6.183354000
H	3.496482000	-1.256004000	-5.529349000
C	5.342420000	-1.210960000	-3.489428000
H	5.714281000	-0.913410000	-2.502682000
H	4.604589000	-2.008720000	-3.351019000
H	6.184293000	-1.631839000	-4.048688000
C	-3.995773000	-5.070548000	-2.454490000
H	-5.038847000	-5.247447000	-2.741545000
H	-3.361863000	-5.304095000	-3.317017000
H	-3.745240000	-5.771822000	-1.651525000
C	-4.716443000	-3.329514000	-0.806107000
H	-4.507791000	-4.002712000	0.031198000
H	-4.590460000	-2.302368000	-0.451603000
H	-5.766752000	-3.463743000	-1.088765000
C	-4.228174000	-2.688769000	-3.155582000
H	-4.097585000	-1.636342000	-2.884022000
H	-3.658702000	-2.887682000	-4.070422000
H	-5.286655000	-2.852403000	-3.385178000
C	-4.427151000	4.286018000	-0.321842000
H	-5.262904000	4.692974000	0.257304000
H	-4.335578000	4.881971000	-1.236472000
H	-4.688766000	3.261538000	-0.604596000
C	-2.885187000	5.790789000	0.922065000
H	-2.725034000	6.418495000	0.038394000

H	-3.742611000	6.193677000	1.473399000
H	-2.003731000	5.882167000	1.564658000
C	-3.376117000	3.485248000	1.782171000
H	-3.544051000	2.435712000	1.517992000
H	-2.513048000	3.527121000	2.454651000
H	-4.250835000	3.850271000	2.333743000
C	-0.404327000	0.862028000	3.596516000
H	-0.392166000	1.945462000	3.681127000
C	-1.531691000	0.150359000	3.983254000
H	-2.383415000	0.698732000	4.376419000
C	-0.472598000	-1.897208000	3.349314000
H	-0.467559000	-2.976385000	3.242330000
C	0.662289000	-1.189386000	2.957878000
H	1.520518000	-1.728934000	2.568058000
C	2.532774000	1.838467000	3.464710000
H	2.179923000	1.894653000	4.491097000
C	3.628409000	2.593941000	3.073055000
H	4.109677000	3.236805000	3.804917000
C	3.446409000	1.700652000	0.870861000
H	3.776727000	1.650551000	-0.159637000
C	3.672338000	-1.647849000	0.473267000
H	4.115765000	-0.694993000	0.732112000
C	3.814963000	-4.018816000	0.306089000
H	4.360428000	-4.953078000	0.408889000
C	2.504555000	-4.054906000	-0.141797000
H	2.049494000	-5.010377000	-0.387939000
C	-0.158973000	-2.370301000	-1.893182000
H	0.488795000	-1.705284000	-2.459073000
C	-1.480449000	-2.551580000	-2.300859000

H	-1.833938000	-1.995567000	-3.161126000
C	-0.509996000	-3.923743000	-0.099212000
H	-0.140711000	-4.461628000	0.770093000
C	-1.826431000	-4.090862000	-0.504777000
H	-2.461790000	-4.771705000	0.055187000
C	-2.036647000	3.241484000	-1.518793000
H	-2.985959000	3.233838000	-2.040662000
C	-0.938256000	2.654404000	-2.141540000
H	-1.055889000	2.205775000	-3.124019000
C	0.415376000	3.169761000	-0.230343000
H	1.374204000	3.170197000	0.276236000
C	-0.684679000	3.751221000	0.385226000
H	-0.558312000	4.180539000	1.375657000
C	3.106175000	0.124010000	-2.313601000
H	3.560514000	-0.762771000	-1.890875000
C	2.958057000	1.756901000	-4.043956000
H	3.291383000	2.181471000	-4.987080000
C	1.902066000	2.360263000	-3.374782000
H	1.431202000	3.242970000	-3.799163000
N	-4.919626000	0.386907000	-0.127747000
H	-5.043449000	0.252195000	0.865163000
C	-6.069055000	0.647105000	-0.863627000
C	-7.291375000	0.656373000	-0.171218000
C	-6.070115000	0.914274000	-2.241461000
C	-8.481074000	0.922321000	-0.836334000
H	-7.295339000	0.452010000	0.897194000
C	-7.272266000	1.177728000	-2.892829000
H	-5.128728000	0.908876000	-2.774890000
C	-8.484026000	1.185175000	-2.205985000

H	-9.413245000	0.923054000	-0.277045000
H	-7.253700000	1.382447000	-3.960454000
H	-9.414551000	1.392691000	-2.726494000

4·(EtO)3SiH E(RPBE1PBE) = -3634.87736861

Au	0.702914000	-0.239399000	-0.059557000
C	-3.812363000	3.137866000	1.410402000
C	-4.387520000	-2.310423000	1.786068000
C	-1.573924000	1.633194000	2.322087000
C	-0.460245000	0.818263000	2.865135000
C	1.658364000	-0.772563000	3.871820000
C	-0.460275000	2.185504000	-2.105686000
C	-1.607457000	1.334274000	-2.511631000
C	-3.877495000	-0.157495000	-3.370774000
C	2.773559000	-1.662397000	4.417551000
C	1.710843000	3.874508000	-1.395012000
C	-5.031860000	3.935110000	0.952989000
C	-5.113986000	-0.964811000	-3.758876000
C	2.721248000	-4.469100000	-2.688774000
C	1.539369000	-3.930087000	-1.883150000
C	-1.941568000	-2.768435000	0.395236000
C	-0.723777000	-3.109516000	-0.374805000
C	-6.140005000	3.802827000	2.008088000
H	-7.023818000	4.372842000	1.700495000
H	-6.437364000	2.755831000	2.134551000
H	-5.819081000	4.182774000	2.983060000
C	-5.576764000	3.437705000	-0.387098000
H	-4.837920000	3.532635000	-1.190519000
H	-5.893940000	2.390040000	-0.336208000
H	-6.450504000	4.034254000	-0.668691000

C	-4.641586000	5.412466000	0.801814000
H	-3.852079000	5.534318000	0.052159000
H	-5.508885000	6.000694000	0.481601000
H	-4.280100000	5.835297000	1.744342000
C	-4.815558000	-2.462911000	-3.595101000
H	-4.560260000	-2.717531000	-2.561421000
H	-3.980455000	-2.768676000	-4.234791000
H	-5.693897000	-3.054623000	-3.876143000
C	-5.536558000	-0.706298000	-5.204894000
H	-6.421804000	-1.305880000	-5.440526000
H	-5.794114000	0.345045000	-5.372194000
H	-4.748631000	-0.984103000	-5.913317000
C	-6.273038000	-0.573338000	-2.829003000
H	-6.035247000	-0.778864000	-1.779814000
H	-7.173665000	-1.142400000	-3.085787000
H	-6.506060000	0.493335000	-2.919779000
C	-3.287490000	2.088246000	0.659439000
H	-3.735396000	1.837164000	-0.295114000
C	-2.179367000	1.347027000	1.088727000
C	-2.077286000	2.710749000	3.059651000
C	-3.168084000	3.446417000	2.616427000
C	-3.342995000	-0.309931000	-2.087395000
H	-3.805569000	-1.019691000	-1.409611000
C	-3.234593000	0.736718000	-4.227254000
C	-2.125987000	1.465617000	-3.801052000
C	-2.226593000	0.403382000	-1.650304000
C	-0.617435000	3.200877000	-1.155150000
C	0.446980000	4.023011000	-0.809436000
H	0.278015000	4.807705000	-0.076849000

C	1.859393000	2.859424000	-2.344991000
C	0.794562000	2.034489000	-2.701474000
C	0.835638000	1.328612000	2.957867000
H	1.041460000	2.337860000	2.611080000
C	1.875488000	0.541901000	3.450012000
H	2.872108000	0.967403000	3.480770000
C	0.349520000	-1.268846000	3.790240000
H	0.133031000	-2.282050000	4.118081000
C	-0.691563000	-0.492867000	3.298787000
H	-1.695623000	-0.904291000	3.252747000
C	-3.852882000	-3.599207000	1.660657000
C	-3.651422000	-1.263452000	1.237736000
C	-2.663885000	-3.817753000	0.981634000
C	-2.440668000	-1.463889000	0.560037000
C	-0.596505000	-2.770828000	-1.724887000
H	-1.387010000	-2.210863000	-2.216046000
C	0.515471000	-3.173141000	-2.461704000
H	0.562851000	-2.899721000	-3.510335000
C	1.411971000	-4.248850000	-0.522338000
C	0.304379000	-3.853992000	0.216892000
H	0.234565000	-4.116109000	1.269177000
C	3.082014000	4.795036000	0.493308000
H	3.839121000	5.539793000	0.767039000
H	3.453021000	3.811046000	0.795022000
H	2.172956000	5.025440000	1.058461000
C	4.151825000	4.471472000	-1.722255000
H	4.933158000	5.175744000	-1.415650000
H	4.063320000	4.535381000	-2.812927000
H	4.477516000	3.463730000	-1.448200000

C	2.455118000	-2.026912000	5.875383000
H	2.392289000	-1.128987000	6.499902000
H	1.504512000	-2.563741000	5.959822000
H	3.241578000	-2.671205000	6.285204000
C	4.136014000	-0.969372000	4.372717000
H	4.421087000	-0.696845000	3.350869000
H	4.150811000	-0.064071000	4.989804000
H	4.900908000	-1.646664000	4.768385000
C	2.851691000	-2.948230000	3.581475000
H	1.921768000	-3.523317000	3.635980000
H	3.045010000	-2.716648000	2.529411000
H	3.661931000	-3.589294000	3.947778000
C	2.775596000	-3.878563000	-4.098818000
H	2.849698000	-2.785390000	-4.076641000
H	3.657770000	-4.262562000	-4.621385000
H	1.897224000	-4.150956000	-4.693065000
C	2.573071000	-5.994771000	-2.798658000
H	2.573144000	-6.467768000	-1.811136000
H	1.637710000	-6.263347000	-3.301520000
H	3.404204000	-6.416344000	-3.375669000
C	-5.594417000	-2.583379000	3.952556000
H	-6.541796000	-2.435362000	4.482884000
H	-5.350578000	-3.649631000	3.994968000
H	-4.813313000	-2.035303000	4.490615000
H	-1.609727000	2.956975000	4.009210000
H	-3.529199000	4.267469000	3.229723000
H	-4.376822000	-4.449676000	2.088131000
H	-2.286164000	-4.830875000	0.873297000
H	-4.015311000	-0.249770000	1.347748000

C	-5.715041000	-2.085728000	2.505283000
C	-6.814420000	-2.876676000	1.781164000
H	-6.918464000	-2.542427000	0.742903000
H	-6.600635000	-3.950113000	1.770530000
H	-7.777460000	-2.731526000	2.283326000
C	-6.114843000	-0.608662000	2.529266000
H	-5.376501000	0.006537000	3.055291000
H	-6.240270000	-0.205277000	1.518043000
H	-7.071050000	-0.498363000	3.050729000
H	2.184189000	-4.832528000	-0.028734000
C	2.840728000	4.834159000	-1.022899000
C	2.431806000	6.255033000	-1.440251000
H	1.522113000	6.582180000	-0.925901000
H	3.230167000	6.964656000	-1.193982000
H	2.248161000	6.311596000	-2.519149000
H	-1.657098000	2.172706000	-4.480283000
H	-3.596382000	0.885390000	-5.239102000
H	-1.586525000	3.362564000	-0.696350000
H	2.822285000	2.692315000	-2.812297000
H	0.943352000	1.255591000	-3.445328000
C	4.042733000	-4.142416000	-1.983661000
H	4.181114000	-3.060247000	-1.901613000
H	4.094733000	-4.568796000	-0.977583000
H	4.884386000	-4.544886000	-2.558165000
P	-1.533194000	0.027790000	0.001814000
O	2.743031000	-0.620635000	-0.216334000
H	2.927894000	-1.438184000	-0.693513000
Si	4.113912000	0.689173000	0.106273000
H	2.950245000	1.381940000	0.723394000

O	4.513053000	0.786294000	-1.531969000
O	5.101538000	2.026439000	0.557079000
O	5.063006000	-0.442885000	0.935912000
C	5.207846000	-1.791871000	0.572987000
H	4.275991000	-2.349911000	0.746942000
H	5.441095000	-1.879782000	-0.499532000
C	6.328736000	-2.418954000	1.374902000
H	6.110902000	-2.376614000	2.446649000
H	6.461077000	-3.469201000	1.092012000
H	7.272375000	-1.892436000	1.197869000
C	4.042780000	0.014319000	-2.606469000
H	4.304769000	-1.047088000	-2.474918000
H	2.947559000	0.064855000	-2.677729000
C	4.672155000	0.514779000	-3.890145000
H	5.763641000	0.445252000	-3.836239000
H	4.330005000	-0.078700000	-4.745230000
H	4.410017000	1.562513000	-4.071917000
C	6.459428000	2.100059000	0.221004000
H	6.974915000	1.161642000	0.480665000
H	6.581548000	2.237732000	-0.865850000
C	7.105970000	3.257300000	0.957227000
H	6.615269000	4.202004000	0.698057000
H	7.020050000	3.119858000	2.040779000
H	8.168989000	3.342012000	0.703985000

5 E(RPBE1PBE) = -2806.76125819

Au	-1.657617000	-0.016577000	0.007172000
C	3.292151000	-3.205576000	-0.359012000
C	3.333787000	1.310506000	2.857463000

C	0.914109000	-2.616542000	1.090399000
C	-0.297937000	-2.349900000	1.898232000
C	-2.659120000	-1.779112000	3.361531000
C	-0.369508000	-0.491425000	-2.992297000
C	0.854218000	0.329387000	-2.844507000
C	3.211726000	1.915344000	-2.666796000
C	-3.927086000	-1.393975000	4.121453000
C	-2.761343000	-2.006780000	-3.175139000
C	4.589320000	-3.528010000	-1.096870000
C	4.478426000	2.752769000	-2.500310000
C	-4.003217000	4.241515000	-0.761153000
C	-2.681949000	3.788133000	-0.143262000
C	0.932593000	2.261823000	1.659246000
C	-0.294944000	2.818779000	1.044658000
C	5.663282000	-3.918224000	-0.070245000
H	6.604988000	-4.152852000	-0.579162000
H	5.851338000	-3.097923000	0.631571000
H	5.369427000	-4.797910000	0.510955000
C	5.105042000	-2.334980000	-1.904230000
H	4.393630000	-2.022597000	-2.676538000
H	5.314609000	-1.471578000	-1.263201000
H	6.038614000	-2.610489000	-2.405490000
C	4.347847000	-4.702077000	-2.056186000
H	3.584737000	-4.448291000	-2.799987000
H	5.272690000	-4.952223000	-2.588186000
H	4.015436000	-5.598730000	-1.523500000
C	4.174844000	3.944339000	-1.578942000
H	3.848151000	3.618131000	-0.586055000
H	3.386814000	4.576326000	-2.002710000

H	5.072361000	4.559745000	-1.450026000
C	4.991680000	3.286531000	-3.837229000
H	5.898510000	3.877456000	-3.672031000
H	5.244445000	2.475229000	-4.528347000
H	4.256190000	3.936008000	-4.323873000
C	5.575986000	1.887116000	-1.862405000
H	5.274333000	1.513507000	-0.878112000
H	6.490944000	2.475355000	-1.729148000
H	5.814786000	1.024842000	-2.494617000
C	2.664693000	-1.965246000	-0.461383000
H	3.089064000	-1.207883000	-1.109325000
C	1.492365000	-1.655250000	0.240853000
C	1.522304000	-3.876086000	1.162562000
C	2.682855000	-4.165555000	0.459534000
C	2.618908000	1.350513000	-1.534079000
H	3.066214000	1.537127000	-0.563736000
C	2.586999000	1.687135000	-3.894359000
C	1.433170000	0.911520000	-3.974840000
C	1.460322000	0.574543000	-1.594147000
C	-0.401732000	-1.833929000	-2.595629000
C	-1.574733000	-2.571674000	-2.688082000
H	-1.562730000	-3.607366000	-2.359565000
C	-2.708438000	-0.679588000	-3.609600000
C	-1.534854000	0.065844000	-3.523832000
C	-1.455507000	-3.109986000	1.718299000
H	-1.459882000	-3.909002000	0.981399000
C	-2.615126000	-2.825403000	2.436268000
H	-3.501451000	-3.419526000	2.241989000
C	-1.477896000	-1.054695000	3.571716000

H	-1.461130000	-0.246901000	4.298278000
C	-0.318845000	-1.331120000	2.858743000
H	0.578721000	-0.748752000	3.043555000
C	2.730313000	2.500153000	3.285349000
C	2.691866000	0.599160000	1.846180000
C	1.558678000	2.959804000	2.700206000
C	1.507629000	1.046068000	1.245591000
C	-0.342097000	3.144258000	-0.312513000
H	0.545729000	3.024488000	-0.925612000
C	-1.515609000	3.619118000	-0.893379000
H	-1.507373000	3.846271000	-1.953964000
C	-2.613846000	3.498039000	1.226959000
C	-1.446578000	3.027747000	1.813755000
H	-1.433051000	2.782894000	2.872683000
C	-4.331587000	-3.368323000	-1.791300000
H	-5.260616000	-3.950122000	-1.790445000
H	-4.440672000	-2.542657000	-1.080290000
H	-3.530553000	-4.019020000	-1.427000000
C	-5.249506000	-2.024932000	-3.668642000
H	-6.144179000	-2.656339000	-3.653414000
H	-5.125386000	-1.653211000	-4.691559000
H	-5.433993000	-1.168932000	-3.010504000
C	-3.686701000	-1.551874000	5.629945000
H	-3.447973000	-2.590813000	5.882890000
H	-2.860794000	-0.922090000	5.975747000
H	-4.584293000	-1.263296000	6.189012000
C	-5.123557000	-2.259856000	3.725769000
H	-5.353874000	-2.167121000	2.658985000
H	-4.954389000	-3.318368000	3.951394000

H	-6.007844000	-1.939075000	4.286531000
C	-4.268166000	0.071314000	3.809484000
H	-3.470506000	0.750102000	4.127099000
H	-4.420023000	0.215169000	2.734533000
H	-5.186132000	0.364716000	4.332103000
C	-3.870360000	4.537360000	-2.255728000
H	-3.559095000	3.650414000	-2.818273000
H	-4.839679000	4.857689000	-2.652179000
H	-3.150062000	5.339459000	-2.451141000
C	-4.494765000	5.513444000	-0.055040000
H	-4.653964000	5.348083000	1.015150000
H	-3.772846000	6.330043000	-0.167262000
H	-5.447662000	5.840732000	-0.486648000
C	4.414837000	0.596102000	4.990840000
H	5.344425000	0.260304000	5.464025000
H	4.091878000	1.510448000	5.498590000
H	3.651039000	-0.171068000	5.158377000
H	1.079636000	-4.631781000	1.806160000
H	3.124379000	-5.153302000	0.560543000
H	3.184415000	3.084042000	4.081398000
H	1.119194000	3.894894000	3.037495000
H	3.115008000	-0.339731000	1.511661000
C	4.637841000	0.828808000	3.489684000
C	5.716044000	1.904432000	3.291989000
H	5.893930000	2.088857000	2.226626000
H	5.431235000	2.854195000	3.755448000
H	6.660466000	1.580757000	3.743692000
C	5.135959000	-0.475816000	2.863054000
H	4.418338000	-1.293543000	2.991662000

H	5.335569000	-0.361118000	1.791624000
H	6.071856000	-0.776677000	3.344858000
H	-3.497791000	3.615174000	1.848173000
C	-4.041325000	-2.839803000	-3.204704000
C	-3.850997000	-4.024785000	-4.163361000
H	-3.014987000	-4.661807000	-3.856599000
H	-4.754545000	-4.645190000	-4.186185000
H	-3.652664000	-3.676563000	-5.183174000
H	0.971333000	0.737708000	-4.943315000
H	2.995372000	2.109499000	-4.806513000
H	0.498056000	-2.302254000	-2.207998000
H	-3.598272000	-0.196983000	-3.999273000
H	-1.533859000	1.106526000	-3.838218000
C	-5.041972000	3.123713000	-0.581160000
H	-4.712962000	2.203345000	-1.075078000
H	-5.208009000	2.892548000	0.475789000
H	-6.002173000	3.426003000	-1.015589000
P	0.707616000	-0.014153000	-0.024277000
H	-3.277915000	-0.021916000	0.025006000

TS3 E(RPBE1PBE) = -3634.86951118

Au	0.779638000	0.042678000	-0.098289000
C	-3.938128000	3.221381000	0.812522000
C	-4.063458000	-2.136164000	2.321247000
C	-1.633207000	2.035841000	1.987155000
C	-0.488718000	1.392434000	2.677292000
C	1.690170000	0.103913000	3.950943000
C	-0.497118000	1.685822000	-2.553875000
C	-1.641282000	0.759292000	-2.739418000
C	-3.933735000	-0.873805000	-3.171834000

C	2.841240000	-0.634665000	4.631327000
C	1.665568000	3.490723000	-2.204931000
C	-5.193094000	3.853997000	0.216252000
C	-5.178806000	-1.743479000	-3.332300000
C	2.714699000	-4.721795000	-2.424452000
C	1.593658000	-4.105741000	-1.587284000
C	-1.691594000	-2.687094000	0.835189000
C	-0.528021000	-3.096875000	0.014437000
C	-6.280180000	3.930333000	1.298118000
H	-7.189600000	4.383317000	0.887773000
H	-6.534598000	2.931494000	1.669815000
H	-5.961363000	4.536347000	2.151954000
C	-5.739479000	3.046752000	-0.963247000
H	-5.021736000	2.988828000	-1.788952000
H	-6.005566000	2.025286000	-0.668787000
H	-6.645159000	3.528112000	-1.346366000
C	-4.856647000	5.269649000	-0.273725000
H	-4.088092000	5.240809000	-1.053978000
H	-5.749346000	5.747979000	-0.692241000
H	-4.487430000	5.901450000	0.540203000
C	-4.850302000	-3.179162000	-2.894732000
H	-4.533055000	-3.222571000	-1.847732000
H	-4.046978000	-3.601415000	-3.508258000
H	-5.733745000	-3.818280000	-3.003680000
C	-5.674343000	-1.772653000	-4.777778000
H	-6.570540000	-2.397972000	-4.845065000
H	-5.938838000	-0.771849000	-5.135732000
H	-4.924233000	-2.193257000	-5.456141000
C	-6.298773000	-1.184680000	-2.441028000

H	-6.010125000	-1.177904000	-1.384589000
H	-7.200076000	-1.800405000	-2.536787000
H	-6.554793000	-0.159157000	-2.729044000
C	-3.358170000	2.072975000	0.276456000
H	-3.791475000	1.617465000	-0.606284000
C	-2.216250000	1.485094000	0.833823000
C	-2.191446000	3.210304000	2.503091000
C	-3.313670000	3.792872000	1.929015000
C	-3.358595000	-0.738661000	-1.904454000
H	-3.796683000	-1.275153000	-1.069221000
C	-3.315632000	-0.202051000	-4.227660000
C	-2.196249000	0.598768000	-4.010200000
C	-2.227554000	0.044869000	-1.673552000
C	-0.656797000	2.868582000	-1.821624000
C	0.402611000	3.749497000	-1.655917000
H	0.237329000	4.658107000	-1.083283000
C	1.813344000	2.315358000	-2.947317000
C	0.751633000	1.429621000	-3.126962000
C	0.789355000	1.952584000	2.650461000
H	0.958586000	2.875429000	2.102051000
C	1.860738000	1.313547000	3.272058000
H	2.845944000	1.753868000	3.173919000
C	0.397715000	-0.436844000	3.994151000
H	0.217688000	-1.368388000	4.524080000
C	-0.672178000	0.189746000	3.369758000
H	-1.661073000	-0.255970000	3.419480000
C	-3.459891000	-3.399832000	2.358103000
C	-3.428751000	-1.154006000	1.565876000
C	-2.308602000	-3.663597000	1.631168000

C	-2.255726000	-1.397738000	0.838427000
C	-0.531565000	-2.939242000	-1.374467000
H	-1.375129000	-2.459006000	-1.859499000
C	0.510228000	-3.430909000	-2.156295000
H	0.453067000	-3.294190000	-3.230743000
C	1.600148000	-4.244511000	-0.190697000
C	0.563435000	-3.755510000	0.595567000
H	0.595840000	-3.886558000	1.673995000
C	3.012418000	4.698976000	-0.480810000
H	3.813729000	5.429912000	-0.317148000
H	3.297564000	3.757521000	0.001660000
H	2.110686000	5.083193000	0.007606000
C	4.118516000	4.003142000	-2.589442000
H	4.905611000	4.736854000	-2.383543000
H	4.052432000	3.890733000	-3.677931000
H	4.423783000	3.044983000	-2.156859000
C	2.561867000	-0.726745000	6.138458000
H	2.490438000	0.271001000	6.585551000
H	1.626919000	-1.257080000	6.347227000
H	3.372802000	-1.266271000	6.641422000
C	4.181028000	0.072513000	4.420210000
H	4.434281000	0.135104000	3.356950000
H	4.174665000	1.082002000	4.846180000
H	4.973280000	-0.491118000	4.925745000
C	2.940401000	-2.049638000	4.042425000
H	2.028416000	-2.628480000	4.220331000
H	3.108576000	-2.001647000	2.962573000
H	3.774606000	-2.594226000	4.499807000
C	2.565409000	-4.402954000	-3.912421000

H	2.580117000	-3.323082000	-4.096842000
H	3.398799000	-4.847805000	-4.466055000
H	1.637693000	-4.810375000	-4.327962000
C	2.681098000	-6.246955000	-2.242509000
H	2.828515000	-6.529991000	-1.195265000
H	1.721419000	-6.660111000	-2.571656000
H	3.475405000	-6.717196000	-2.833503000
C	-5.123939000	-2.125525000	4.580683000
H	-6.048371000	-1.951477000	5.142606000
H	-4.807710000	-3.156570000	4.767920000
H	-4.352551000	-1.457247000	4.978940000
H	-1.742109000	3.658663000	3.385219000
H	-3.714938000	4.701536000	2.369774000
H	-3.902132000	-4.198562000	2.947440000
H	-1.882342000	-4.663035000	1.648441000
H	-3.845897000	-0.154849000	1.545478000
C	-5.356533000	-1.865834000	3.085647000
C	-6.448344000	-2.811704000	2.564444000
H	-6.632192000	-2.645990000	1.497039000
H	-6.172050000	-3.861957000	2.700617000
H	-7.387001000	-2.638952000	3.102767000
C	-5.840057000	-0.424536000	2.909894000
H	-5.109394000	0.302151000	3.281937000
H	-6.050971000	-0.191479000	1.859977000
H	-6.767462000	-0.280522000	3.473597000
H	2.422874000	-4.761117000	0.296718000
C	2.800232000	4.490574000	-1.987961000
C	2.420003000	5.826205000	-2.644310000
H	1.503838000	6.243630000	-2.213534000

H	3.221911000	6.559554000	-2.499731000
H	2.261230000	5.705597000	-3.721964000
H	-1.749574000	1.132933000	-4.844692000
H	-3.707589000	-0.284602000	-5.235932000
H	-1.623147000	3.103119000	-1.387026000
H	2.774151000	2.065817000	-3.383101000
H	0.901443000	0.516181000	-3.697180000
C	4.074322000	-4.187909000	-1.955556000
H	4.136569000	-3.104327000	-2.098814000
H	4.258923000	-4.406879000	-0.899515000
H	4.881304000	-4.648817000	-2.536157000
P	-1.475022000	0.031939000	-0.001629000
O	2.803910000	-0.943056000	-0.727789000
H	3.051828000	-1.868010000	-0.625320000
Si	3.794405000	0.389680000	-0.040201000
H	2.372011000	1.068441000	0.060628000
O	4.693970000	0.713348000	-1.430474000
O	4.254254000	1.824207000	0.794883000
O	4.623974000	-0.598875000	1.064953000
C	4.982803000	-1.938908000	0.837579000
H	4.121295000	-2.607798000	1.001106000
H	5.317254000	-2.090937000	-0.199175000
C	6.103346000	-2.330382000	1.777660000
H	5.795010000	-2.212712000	2.820860000
H	6.391364000	-3.375334000	1.618333000
H	6.982423000	-1.700093000	1.608457000
C	4.771362000	-0.119107000	-2.565646000
H	5.199618000	-1.097789000	-2.302834000
H	3.770644000	-0.314836000	-2.973086000

C	5.647035000	0.539464000	-3.610946000
H	6.647603000	0.732854000	-3.210056000
H	5.746589000	-0.107798000	-4.489211000
H	5.222443000	1.494842000	-3.935531000
C	5.603924000	2.161950000	1.000832000
H	6.195620000	1.268046000	1.245701000
H	6.032592000	2.589556000	0.080330000
C	5.713498000	3.168715000	2.129222000
H	5.124303000	4.065493000	1.908572000
H	5.342133000	2.740947000	3.066827000
H	6.755505000	3.473096000	2.281044000

6. References

[1] Sheldrick, G. M. A short history of SHELX. *Acta Cryst*. **2008,** *A64*, 112-122.

[2] Frisch, M. J.; Trucks, G. W.; Schlegel, H. B.; Scuseria, G. E.; Robb, M. A.; Cheeseman, J. R.; Scalmani, G.; Barone, V.; Mennucci, B; Petersson, G. A.; Nakatsuji, H.; Caricato, M.; Li, X.; Hratchian, H. P.; Izmaylov, A. F.; Bloino, J.; Zheng, G.; Sonnenberg, J. L.; Hada, M.; Ehara, M.; Toyota, K.; Fukuda, R.; Hasegawa, J.; Ishida, M.; Nakajima, T.; Honda, Y.; Kitao, O.; Nakai, H.; Vreven, T.; Montgomery, J. A. J.; Peralta, J. E.; Ogliaro, F.; Bearpark, M.; Heyd, J. J.; Brothers, E.; Kudin, K. N.; Staroverov, V. N.; Kobayashi, R.; Normand, J.; Raghavachari, K.; Rendell, A.; Burant, J. C.; Iyengar, S. S.; Tomasi, J.; Cossi, M.; Rega, N.; Millam, J. M.; Klene, M.; Knox, J. E.; Cross, J. B.; Bakken, V.; Adamo, C.; Jaramillo, J.; Gomperts, R.; Stratmann, R. E.; Yazyev, O.; Austin, A. J.; Cammi, R.; Pomelli, C.; Ochterski, J. W.; Martin, R. L.; Morokuma, K.; Zakrzewski, V. G.; Voth, G. A.; Salvador, P.; Dannenberg, J. J.; Dapprich, S.; Daniels, A. D.; Farkas, O.; Foresman, J. B.; Ortiz, J. V.; Cioslowski, J.; Fox, D. J.; Gaussian 09, Revision D.01, Gaussian, Inc.: Wallingford CT, **2013**.

[3] Perdew, J. P.; Burke, K.; Ernzerhof. M. Generalized Gradient Approximation Made Simple. *Phys. Rev. Lett.* **1996**, *77*, 3865.

[4] Grimme, S.; Antony, J.; Ehrlich, S.; Krieg. H. A consistent and accurate ab initio parametrization of density functional dispersion correction (DFT-D) for the 94 elements H-Pu. *J. Chem. Phys*. **2010**, *132*, 154104.

[5] a) Hehre, W. J.; Ditchfield, R.; Pople. J. A.; Self—Consistent Molecular Orbital Methods. XII. Further Extensions of Gaussian—Type Basis Sets for Use in Molecular Orbital Studies of Organic Molecules. *J. Phys. Chem.* **1972**, *56*, 2257; b) Hariharan, P. C.; Pople. J. A. The influence of polarization functions on molecular orbital hydrogenation energies. *Theor. Chim. Acta.* **1973**, *28*, 213-222; c) Francl, M. M.; Pietro, W. J.; Hehre, W. J.; Binkley, J. S.; Gordon, M. S.; Defrees, D. J.; Pople. J. A. Self‐consistent molecular orbital methods. XXIII. A polarization‐type basis set for second‐row elements. *J. Chem. Phys.* **1982**, *77*, 3654.

[6] Andrae, D.; Haeussermann, U.; Dolg, M.; Stoll, H.; Preuss. H. Energy-adjustedab initio pseudopotentials for the second and third row transition elements. *Theor. Chim. Acta* **1990**, *77*, 123-141.

[7] Marenich, A. V.; Cramer, C. J.; Truhlar, D. G. Universal Solvation Model Based on Solute Electron Density and on a Continuum Model of the Solvent Defined by the Bulk Dielectric Constant and Atomic Surface Tensions. *J. Phys. Chem. B* **2009**, *113*, 6378-6396.
